# Supplementary material for: Chloroaluminate Ionic Liquid Immobilized on Magnetic Nanoparticles as a Heterogeneous Lewis Acidic Catalyst for the Friedel–Crafts Sulfonylation of Aromatic Compounds
Source: Molecules. 2022 Mar 2;27(5):1644. doi: 10.3390/molecules27051644 (PMC8911771; doi:10.3390/molecules27051644)
Supplement: Supplementary file 1 [file molecules-27-01644-s001.zip › molecules-1593700-supplementary_final.pdf]

molecules

Supplementary

Chloroaluminate ionic liquid immobilized on magnetic nanoparticle as a heterogeneous Lewis acidic catalyst for the Friedel–Crafts sulfonylation of aromatic compounds

Ngoc-Lan Thi Nguyen[1], Quoc-Anh Nguyen[1], Tien Khoa Le[1], Thi Xuan Thi Luu*[1,2], Kim-Ngan Thi Tran[1] and Phuoc-Bao Pham[1]

[1] Ngoc-Lan Thi Nguyen, Quoc- Anh Nguyen, Tien Khoa Le, Thi Xuan Thi Luu, Kim-Ngan Thi Tran and Phuoc-Bao Pham

Faculty of Chemistry, University of Science

227 Nguyen Van Cu St, Dist. 5, Ho Chi Minh City, Vietnam

[2] Vietnam National University of Hochiminh City, Vietnam

* Correspondence: ltxthi@hcmus.edu.vn

molecules

Contents	Pages
Surface area of $Fe_3O_4@O_2Si[PrMIM]Cl·AlCl_3$ (Figure S1)	2
NMR spectra and HRMS of unknown-products	3-72
*1-((4-chlorophenyl)sulfonyl)-2-methylbenzene (**3c′**) (Figure S2-S4)*	3-5
*1-methyl-2-((4-nitrophenyl)sulfonyl)benzene (**3d′**) (Figure S5-S7)*	6-8
*1-((4-chlorophenyl)sulfonyl)-2-methoxybenzene (**3h′**) (Figure S8-S10)*	9-11
*1-methoxy-2-((4-nitrophenyl)sulfonyl)benzene (**3i′**) (Figure S11-S13)*	12-14
*1-(ethylsulfonyl)-4-methoxybenzene (**3j**) (Figure S14-S16)*	15-17
*1-(Ethylsulfonyl)-2-methoxybenzene (**3j′**) (Figure S17-S19)*	18-20
*1-(Isobutylsulfonyl)-4-methoxybenzene (**3k**) (Figure S20-S22)*	21-23
*1-(Isobutylsulfonyl)-2-methoxybenzene (**3k′**) (Figure S23-S25)*	24-26
*1-Methoxy-4-(octylsulfonyl)benzene (**3l**) (Figure S26-S28)*	27-29
*1-Methoxy-2-(octylsulfonyl)benzene (**3l′**) (Figure S29-S31)*	30-32
*2,3-dimethyl-1-tosylbenzene (**3o′**) (Figure S32-S34)*	33-35
*2,4-dimethoxy-1-tosylbenzene (**3p**) (Figure S35-S37)*	36-38
*1,3-dimethoxy-2-tosylbenzene (**3p′**) (Figure S38-S40)*	39-41
*1,4-dimethoxy-2-tosylbenzene (**3q**) (Figure S41-S43)*	42-44
*4-((4-chlorophenyl)sulfonyl)phenol (**3r**) (Figure S44-S46)*	45-47
*2-((4-chlorophenyl)sulfonyl)phenol (**3r′**) (Figure S47-S49)*	48-50
*2-(phenylsulfonyl)naphthalene (**3s′**) (Figure S50-S52)*	51-53
*4-(phenylsulfonyl)dibenzo[b,d]thiophene (**3t′**) (Figure S53-S55)*	54-56
*4-methoxy-2-methyl-1-tosylbenzene (**3v**) (Figure S56-S58)*	57-59
*2-methoxy-4-methyl-1-tosylbenzene (**3v′**) (Figure S59-S61)*	60-62
*1-methoxy-3-methyl-1-tosylbenzene (**3v′′**) (Figure S62-S64)*	63-65
*1,2-dimethoxy-4-tosylbenzene (**3w**) (Figure S65-S67)*	66-68
*1,2-dimethoxy-3-tosylbenzene (**3w′**) (Figure S68-S70)*	69-71

Surface area of $Fe_3O_4@O_2Si[PrMIM]Cl{\cdot}AlCl_3$

Multi-Point BET Plot

Data Reduction Parameters

**Adsorbate**	**Nitrogen**	**Temperature**	77.350K		
	**Molec. Wt.:** 28.013	**Cross Section:**	16.200 Å²	**Liquid Density:**	0.808 g/cc

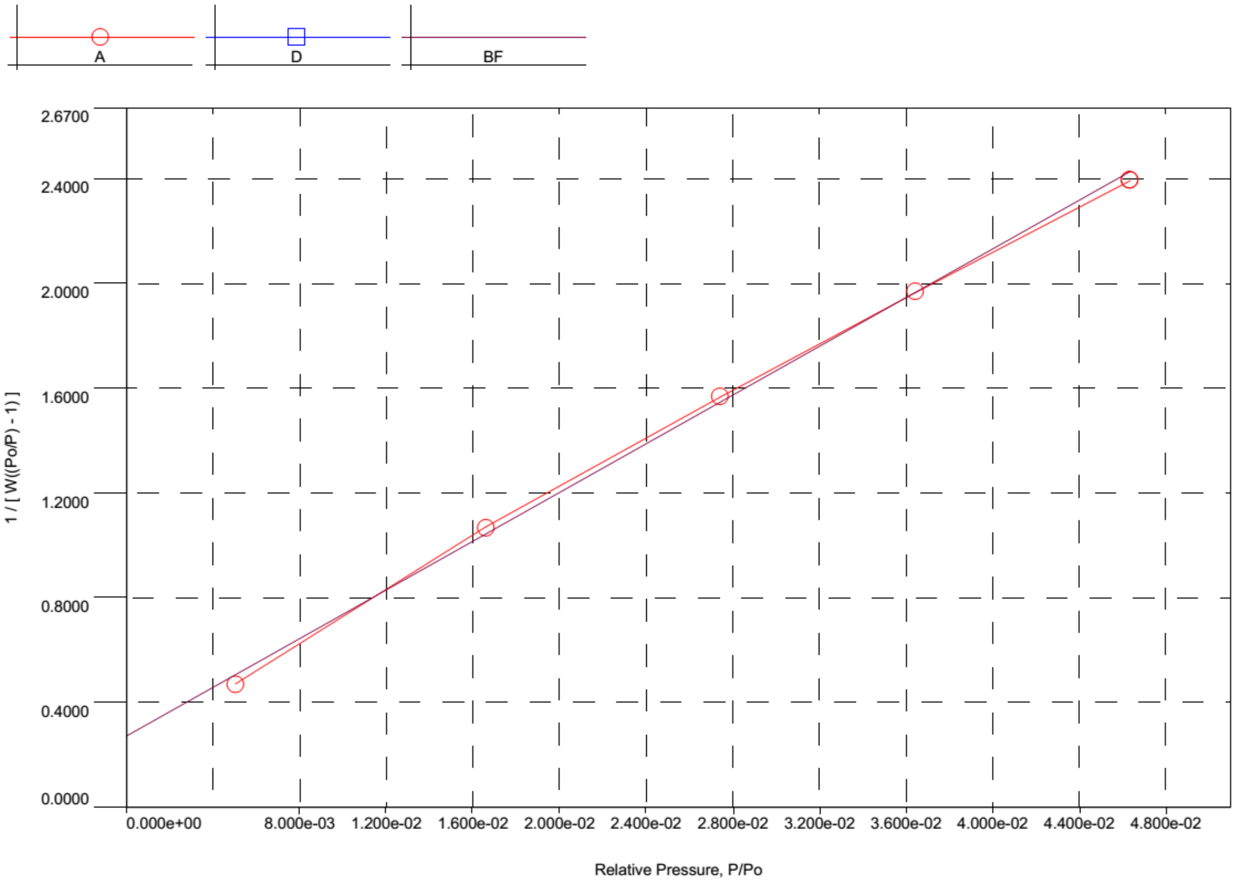

BET summary

Slope = 46.511
Intercept = 2.691e-01
Correlation coefficient, r = 0.999285
C constant= 173.844

Surface Area = 74.444 m²/g

Figure S1. BET surface area of $Fe_3O_4@O_2Si[PrMIM]Cl{\cdot}AlCl_3$

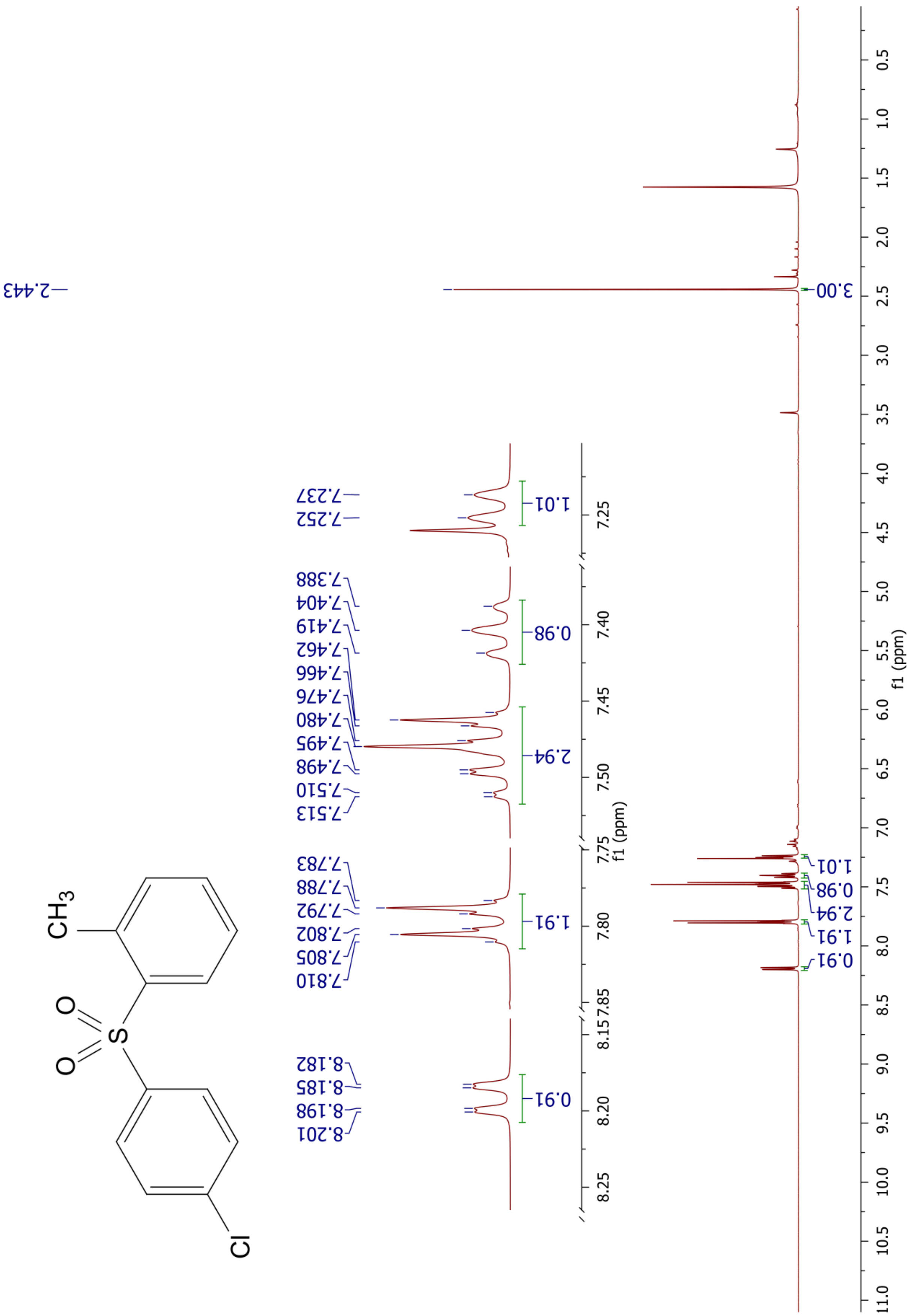

Figure S2. ^{1}H-NMR of 1-((4-chlorophenyl)sulfonyl)-2-methylbenzene (**3c'**)

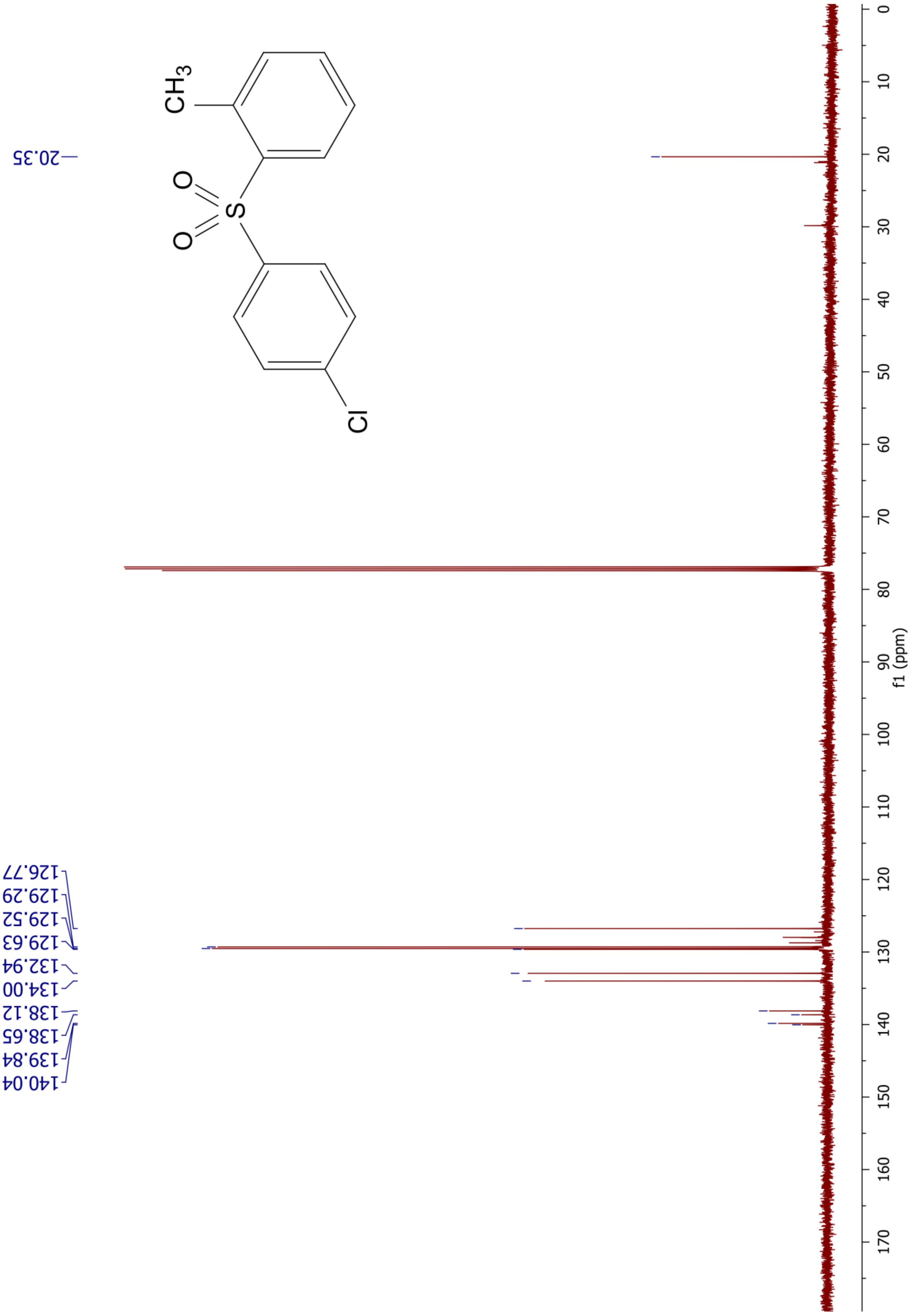

Figure S3. ^{13}C-NMR of 1-((4-chlorophenyl)sulfonyl)-2-methylbenzene (**3c’**)

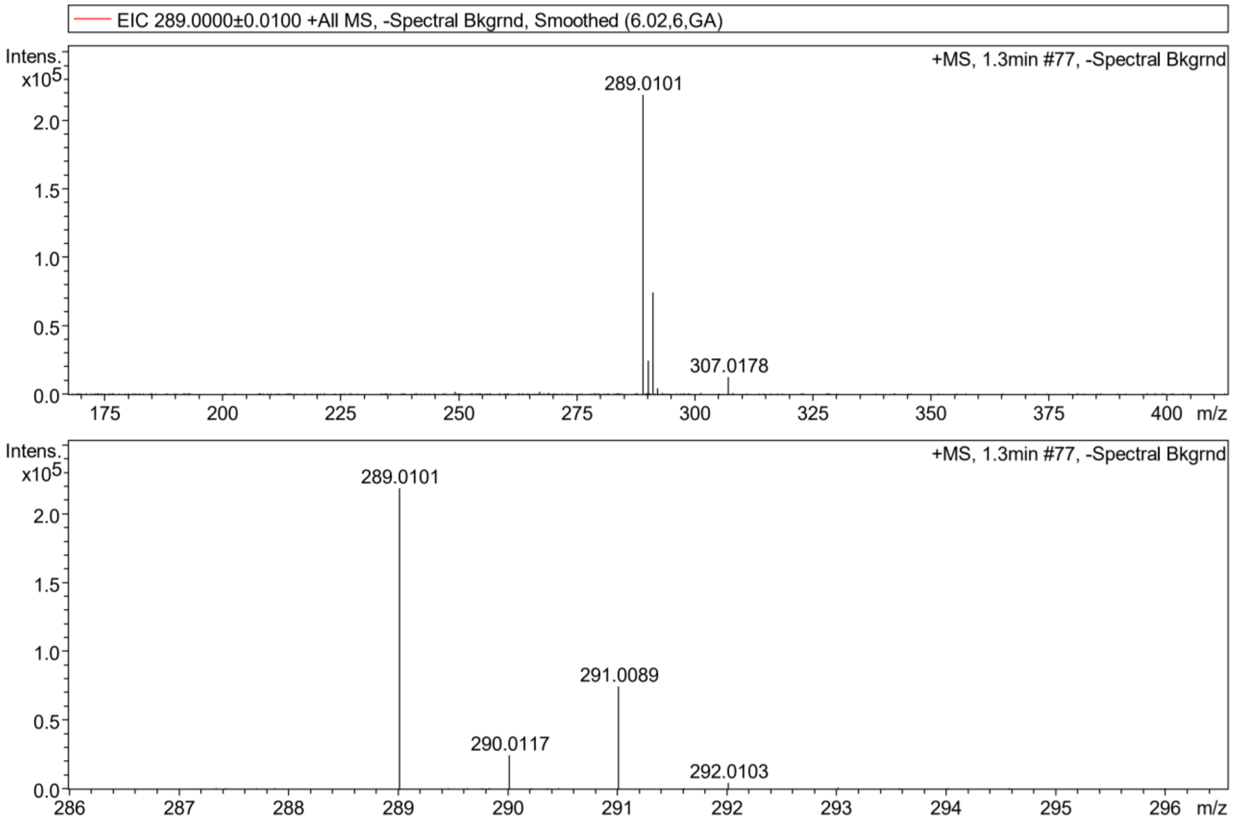

Figure S4. HRMS of 1-((4-chlorophenyl)sulfonyl)-2-methylbenzene (**3c'**)

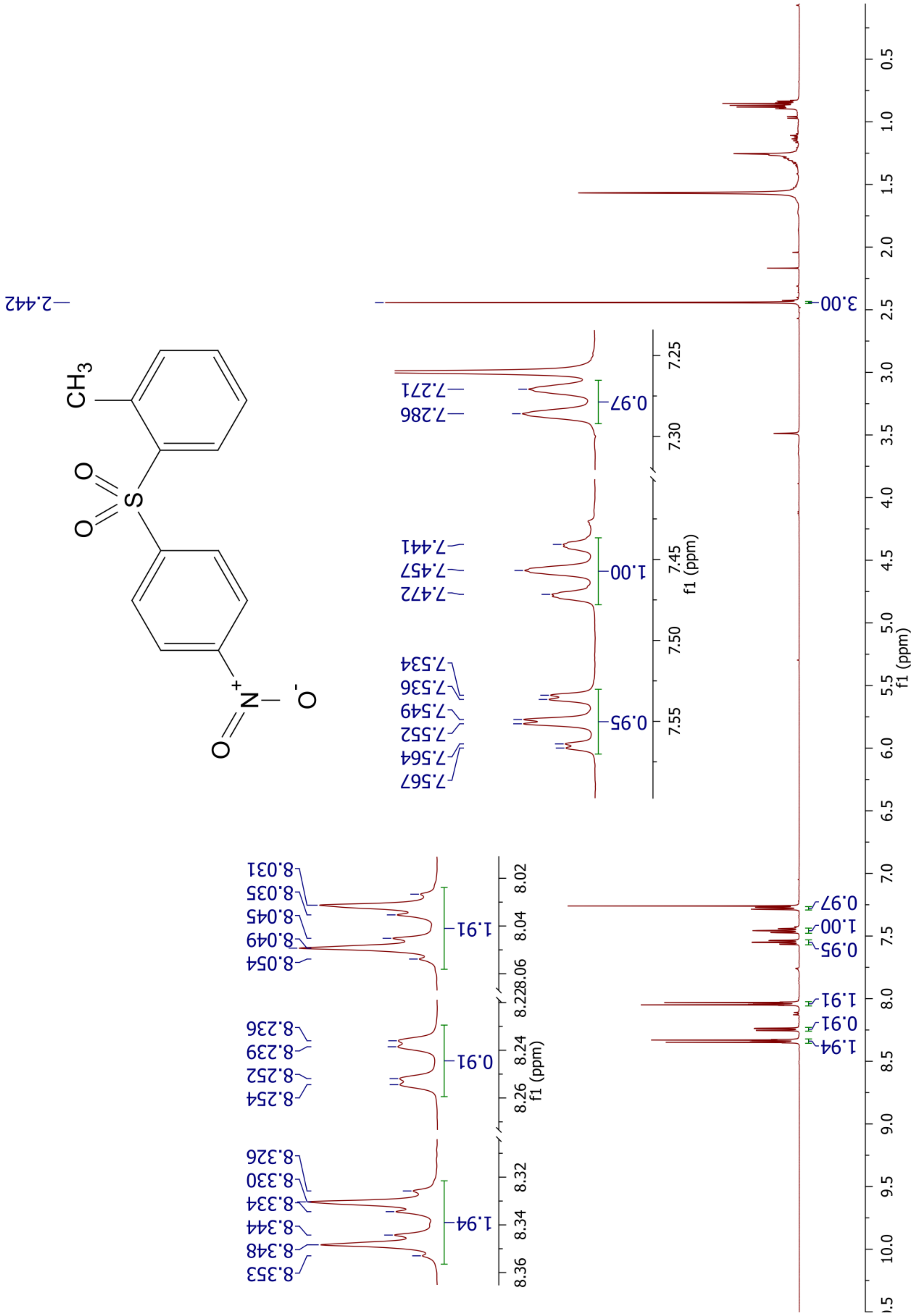

Figure S5. ^{1}H-NMR of 1-methyl-2-((4-nitrophenyl)sulfonyl)benzene (**3d'**)

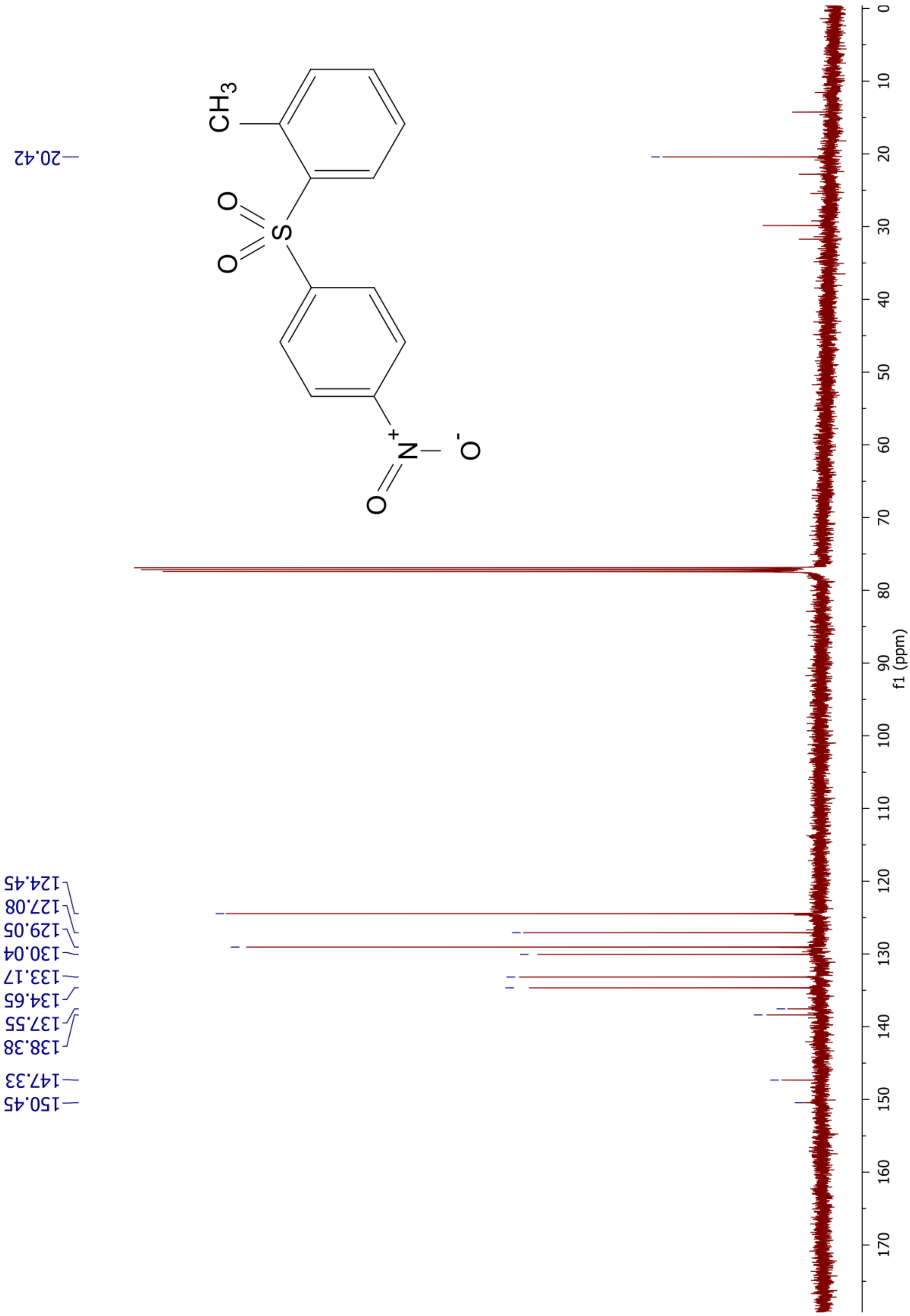

Figure S6. ^{13}C-NMR of 1-methyl-2-((4-nitrophenyl)sulfonyl)benzene (**3d'**)

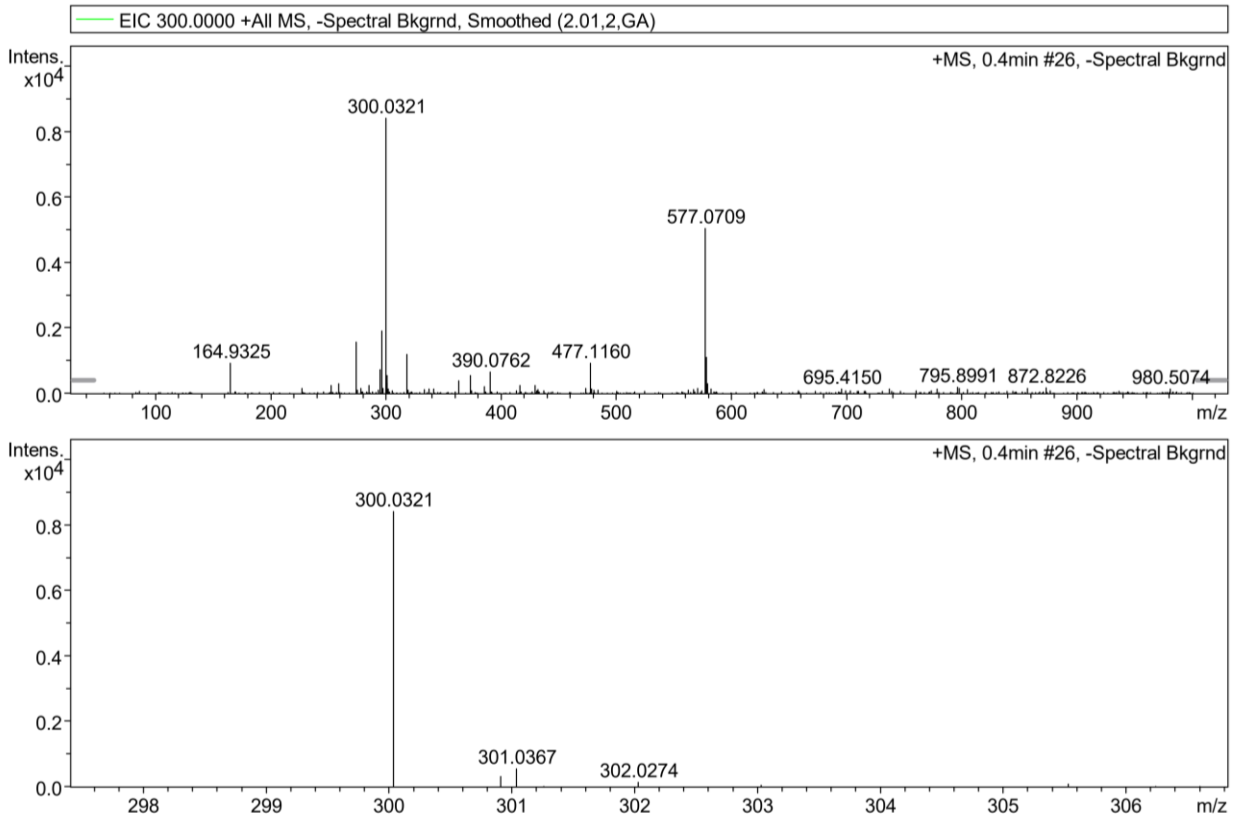

Figure S7. HRMS of 1-methyl-2-((4-nitrophenyl)sulfonyl)benzene (**3d'**)

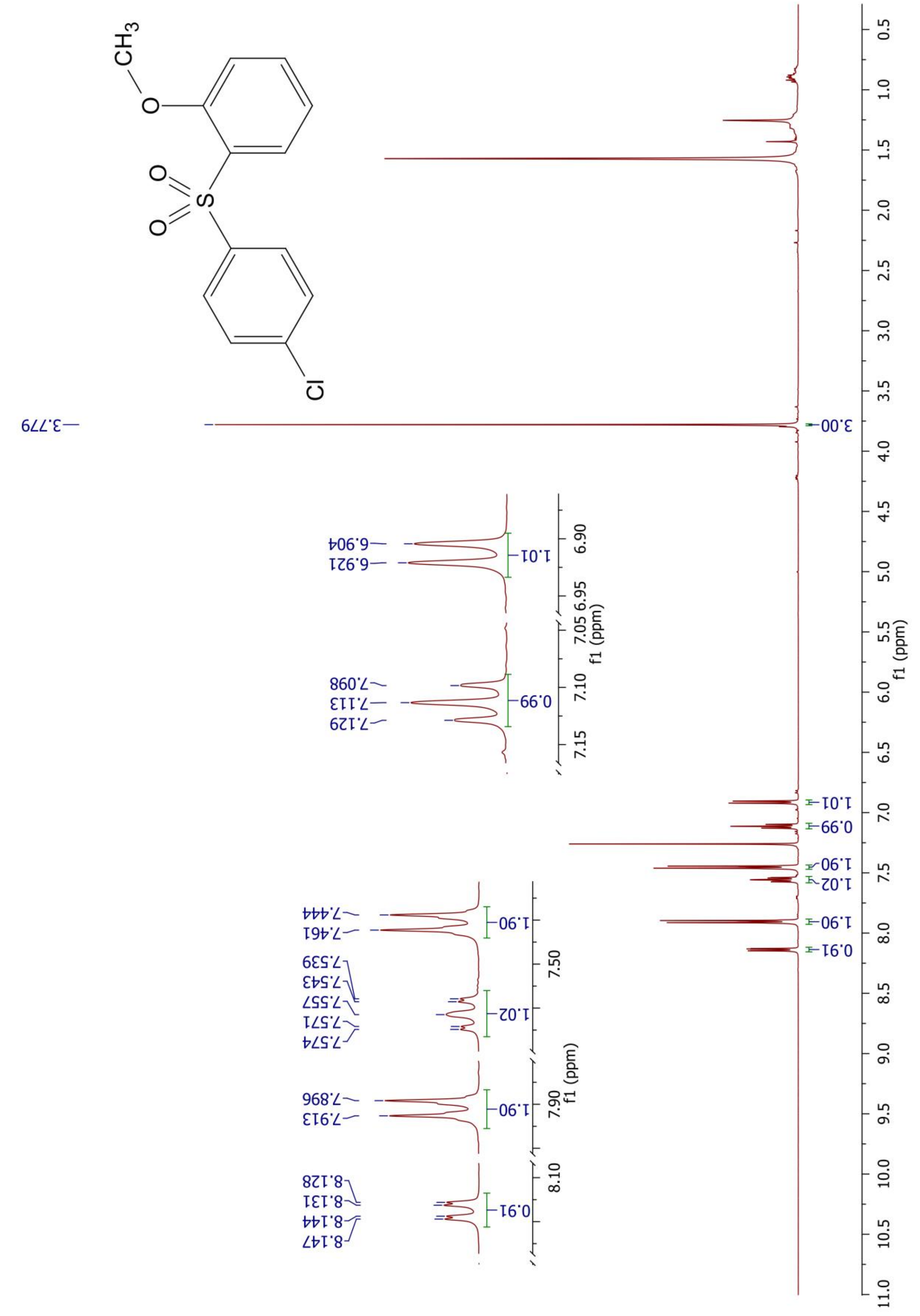

Figure S8. ^{1}H-NMR of 1-((4-chlorophenyl)sulfonyl)-2-methoxybenzene (**3h'**)

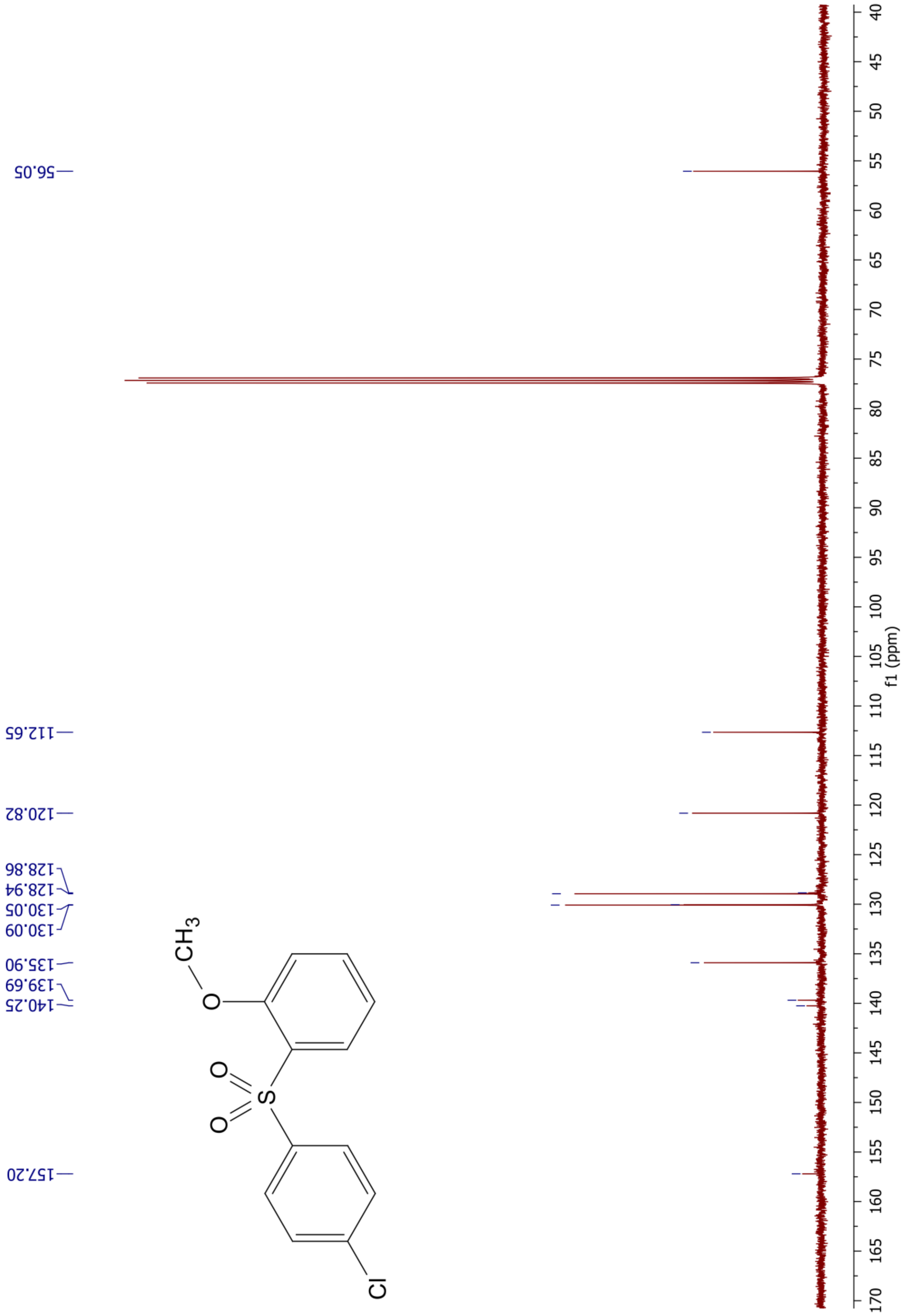

Figure S9. ^{13}C-NMR of 1-((4-chlorophenyl)sulfonyl)-2-methoxybenzene (**3h'**)

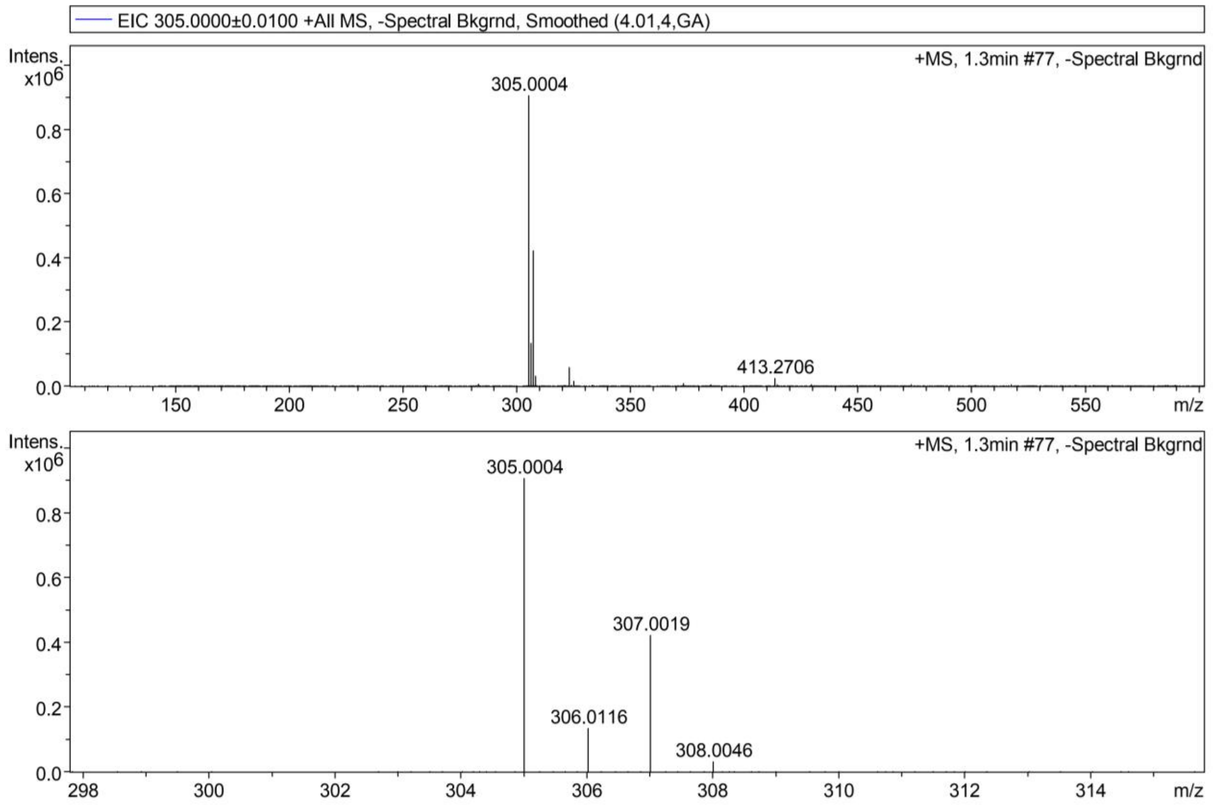

Figure S10. HRMS of 1-((4-chlorophenyl)sulfonyl)-2-methoxybenzene (**3h'**)

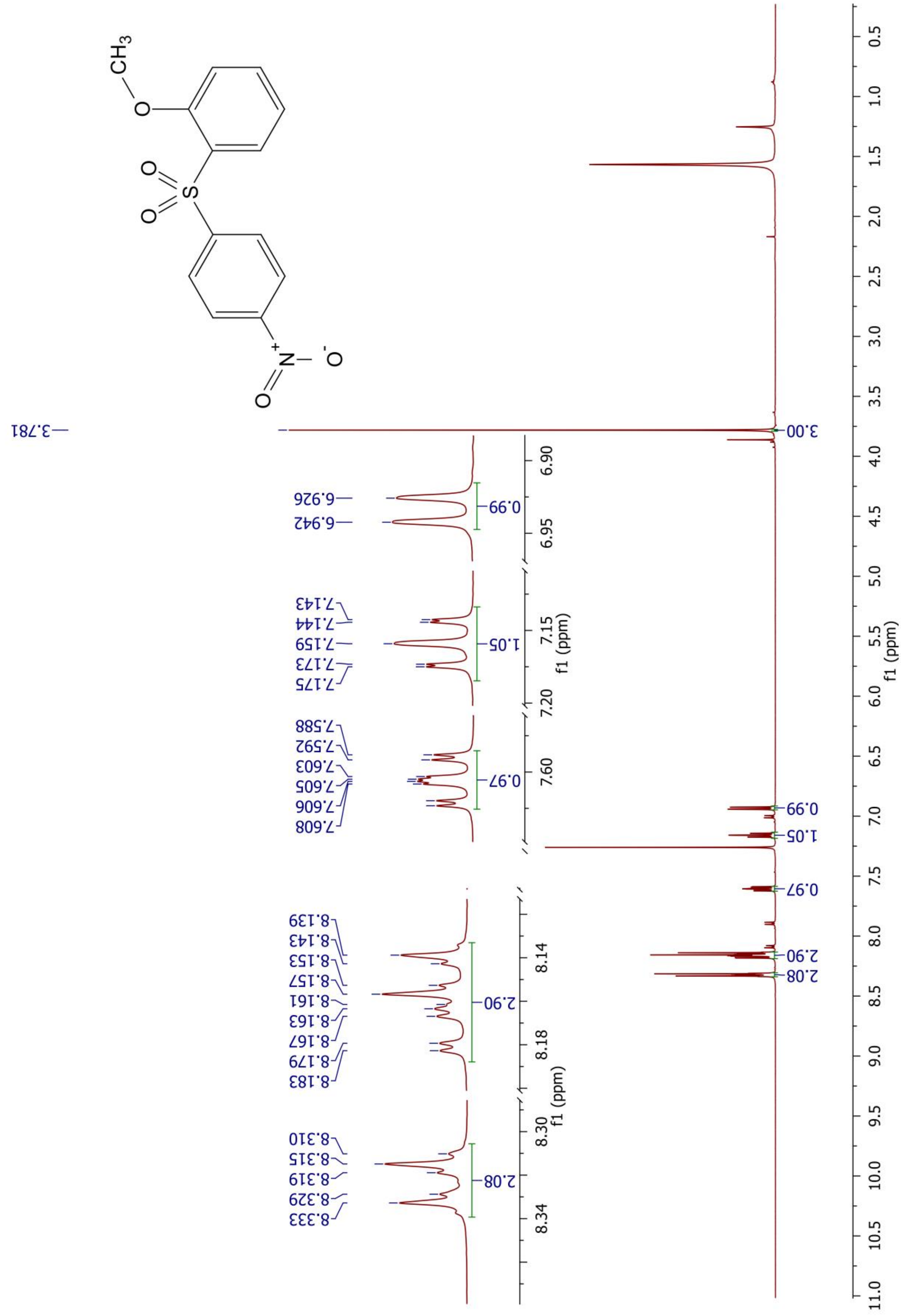

Figure S11. ^{1}H-NMR of 1-methoxy-2-((4-nitrophenyl)sulfonyl)benzene (**3i'**)

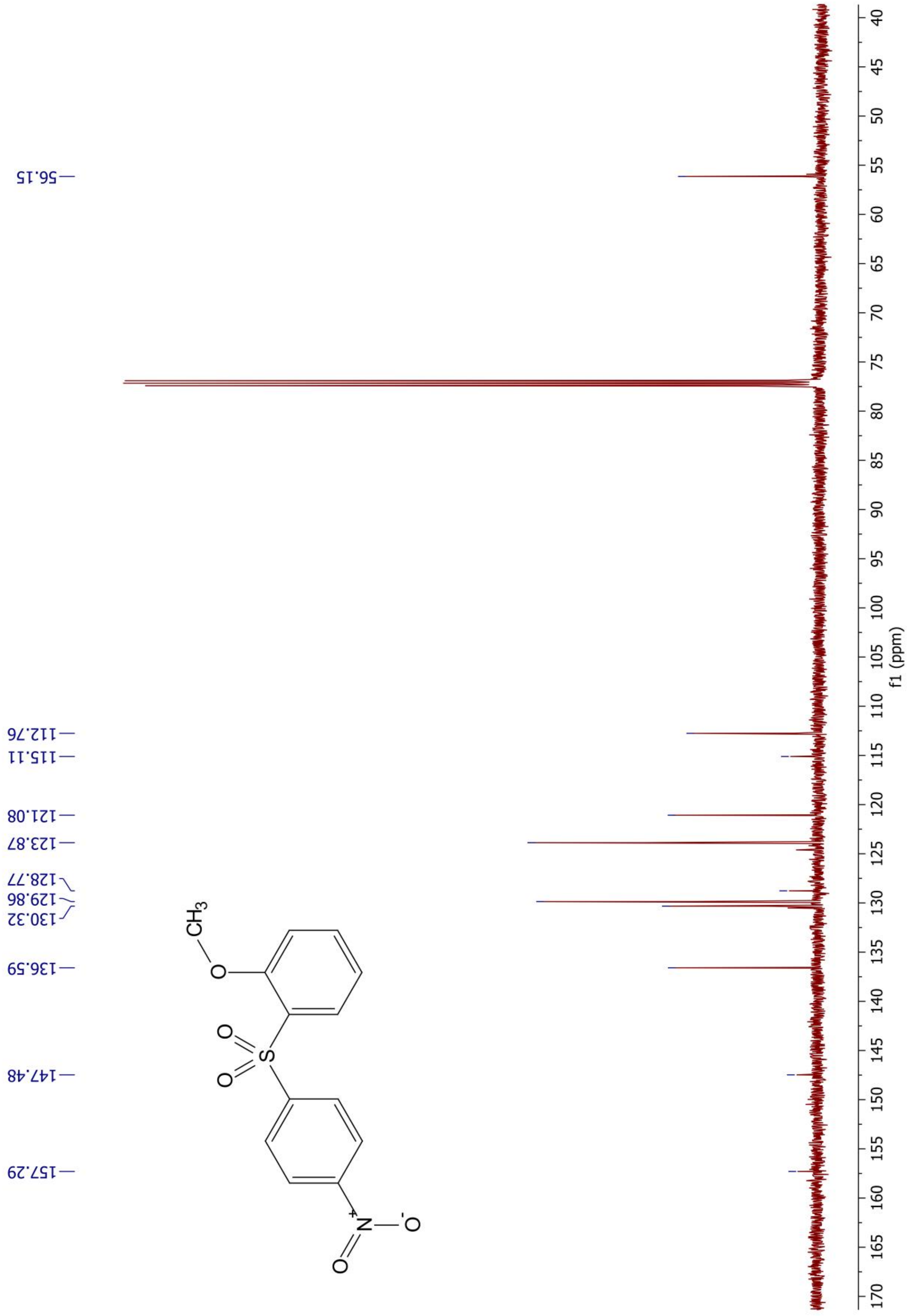

Figure S12. ^{13}C-NMR of 1-methoxy-2-((4-nitrophenyl)sulfonyl)benzene (**3i'**)

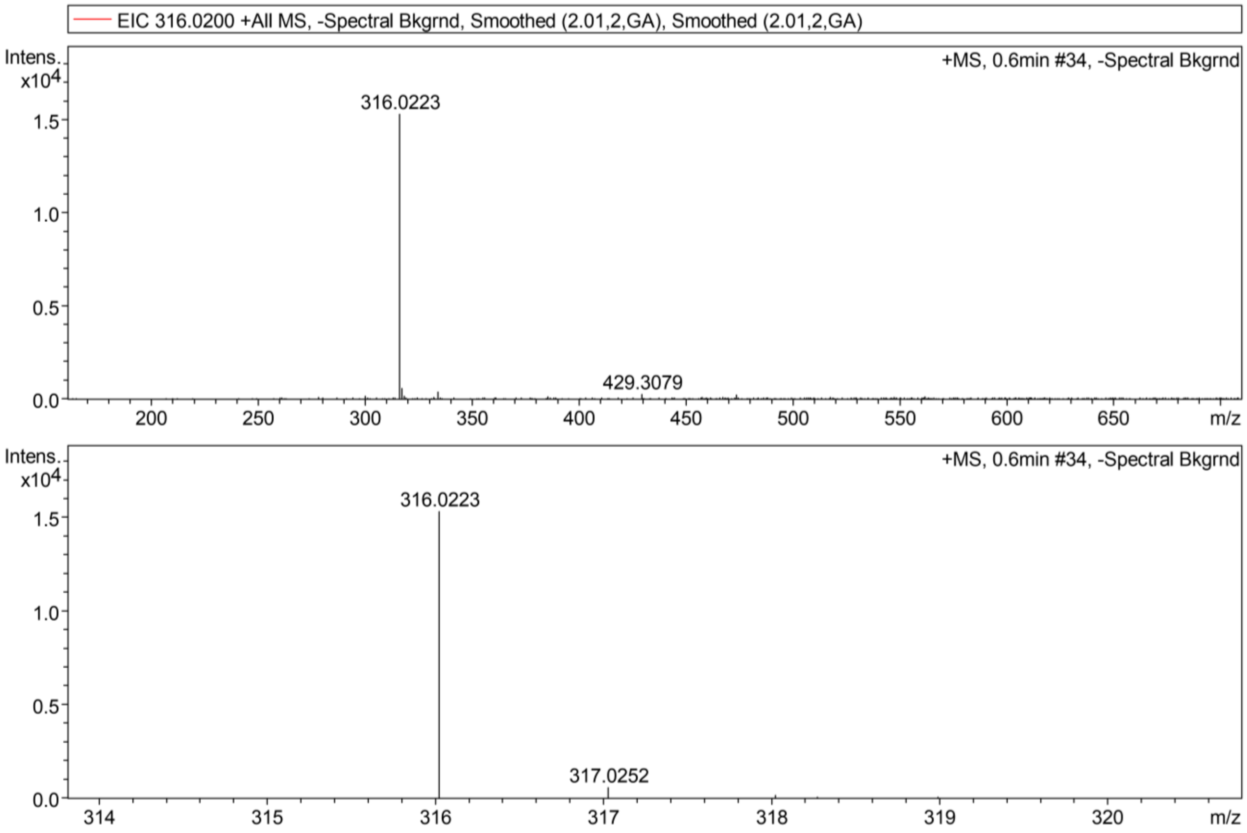

Figure S13. HRMS of 1-methoxy-2-((4-nitrophenyl)sulfonyl)benzene (**3i'**)

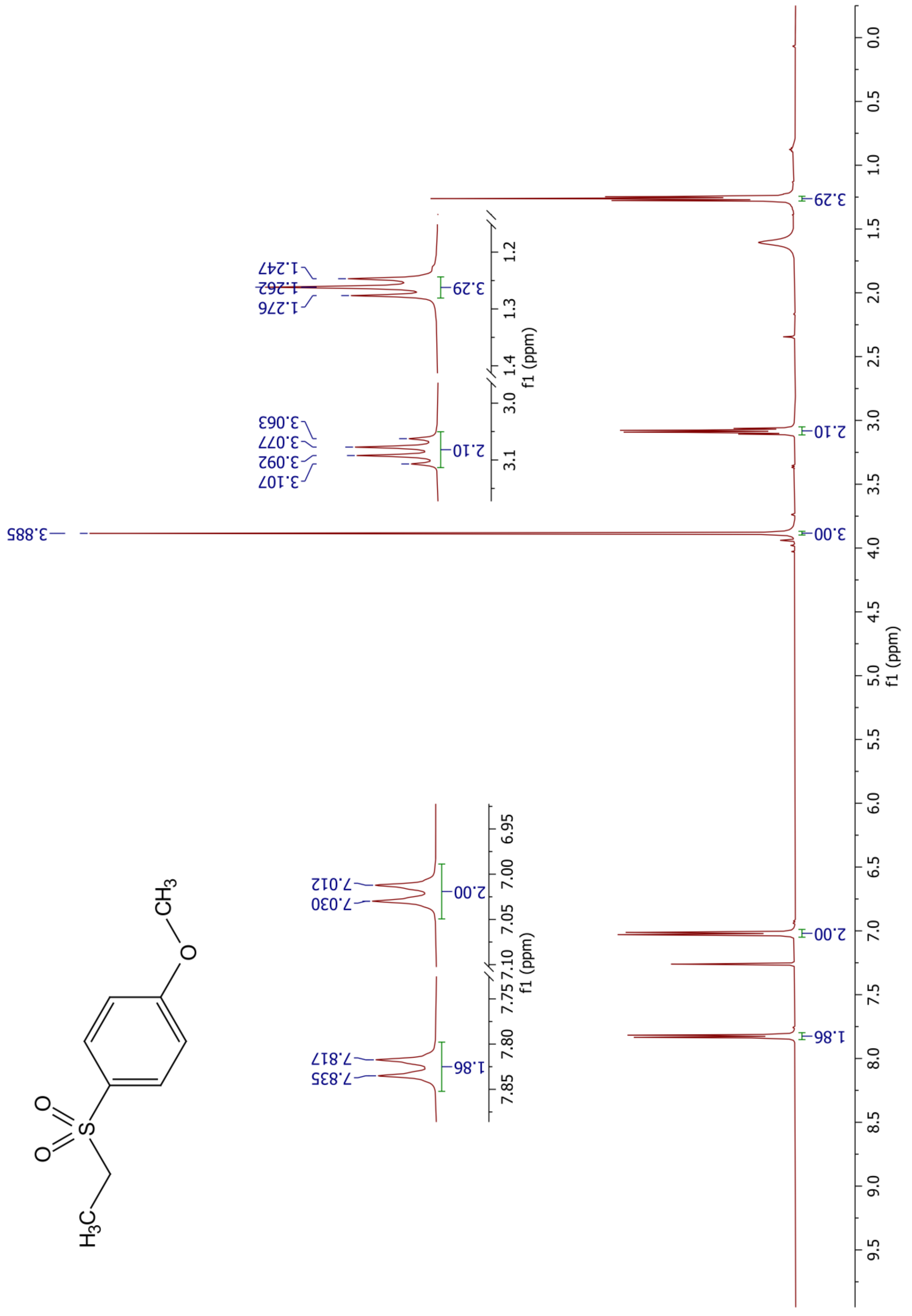

Figure S14. ^{1}H-NMR of 1-(ethylsulfonyl)-4-methoxybenzene (**3j**)

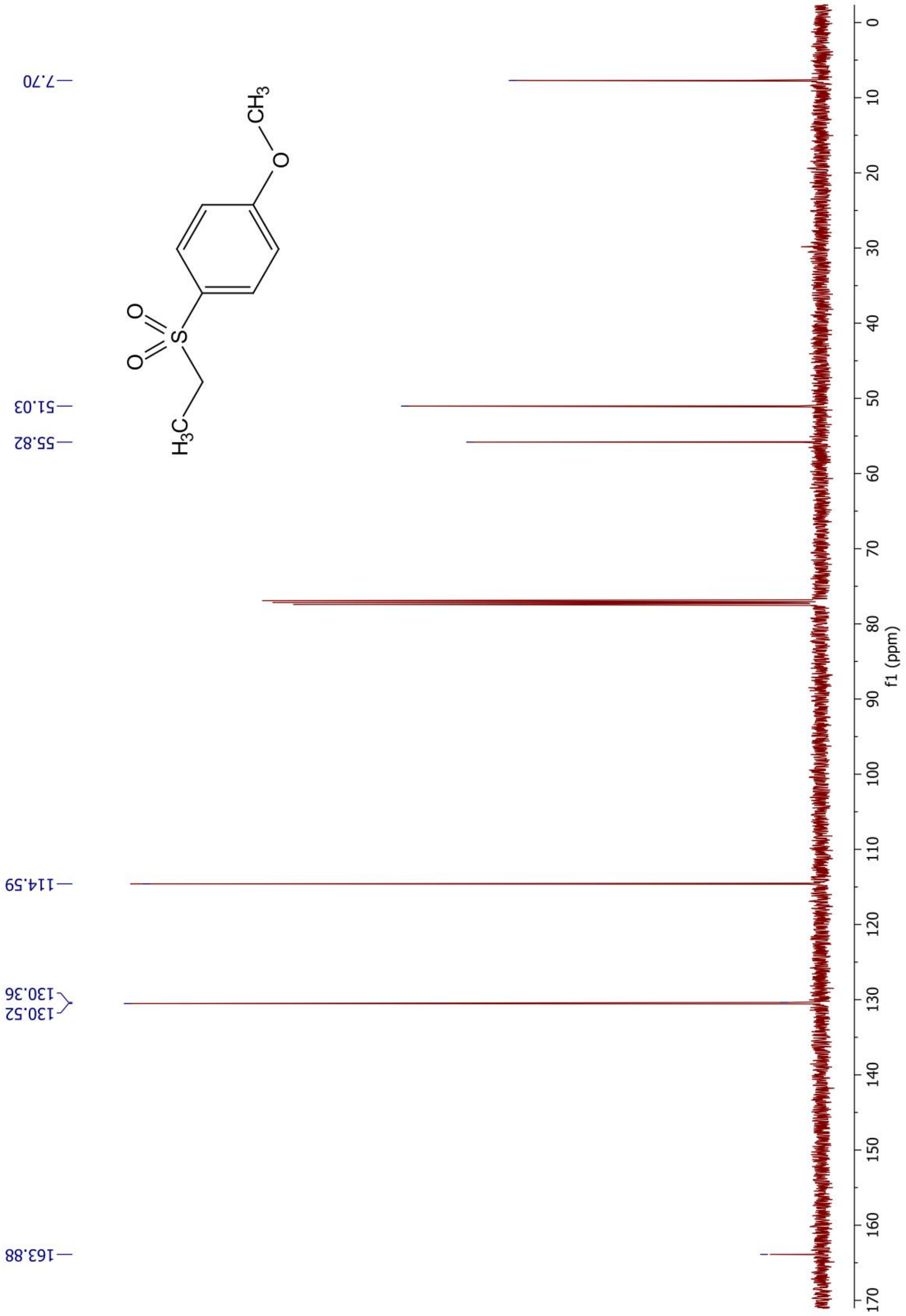

Figure S15. ^{13}C-NMR of 1-(ethylsulfonyl)-4-methoxybenzene (**3j**)

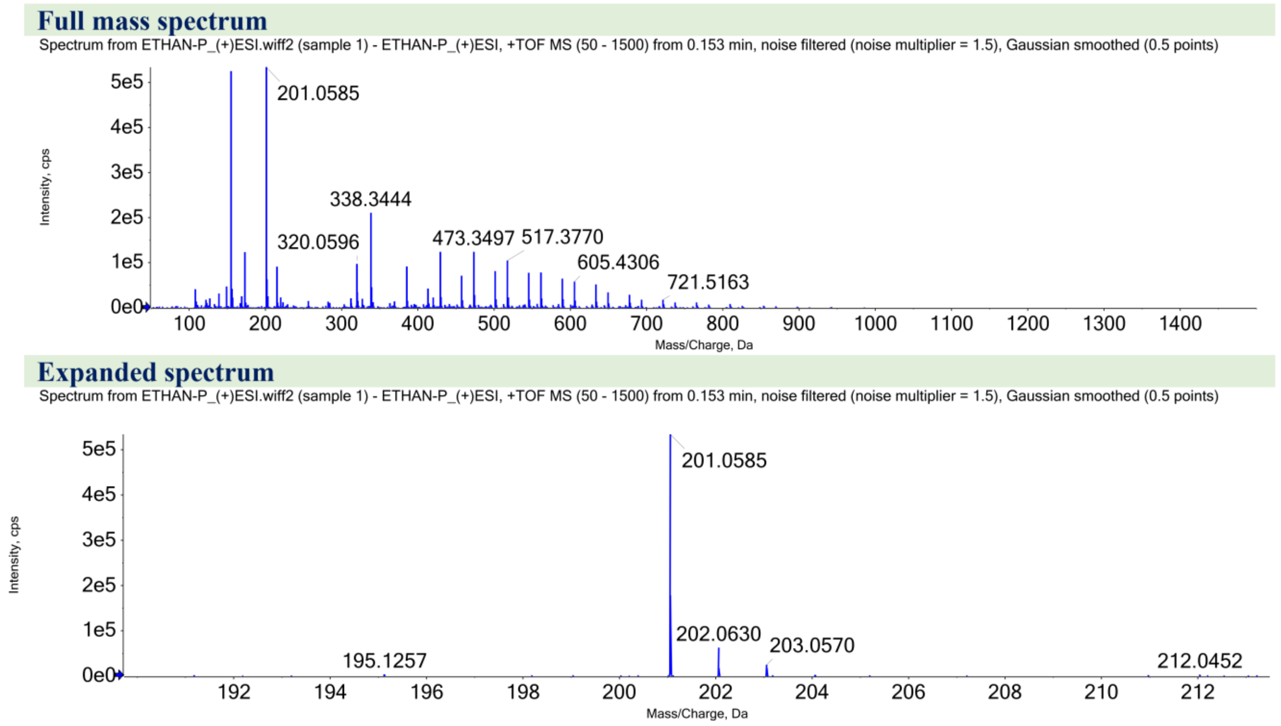

Figure S16. HRMS of 1-(ethylsulfonyl)-4-methoxybenzene (**3j**)

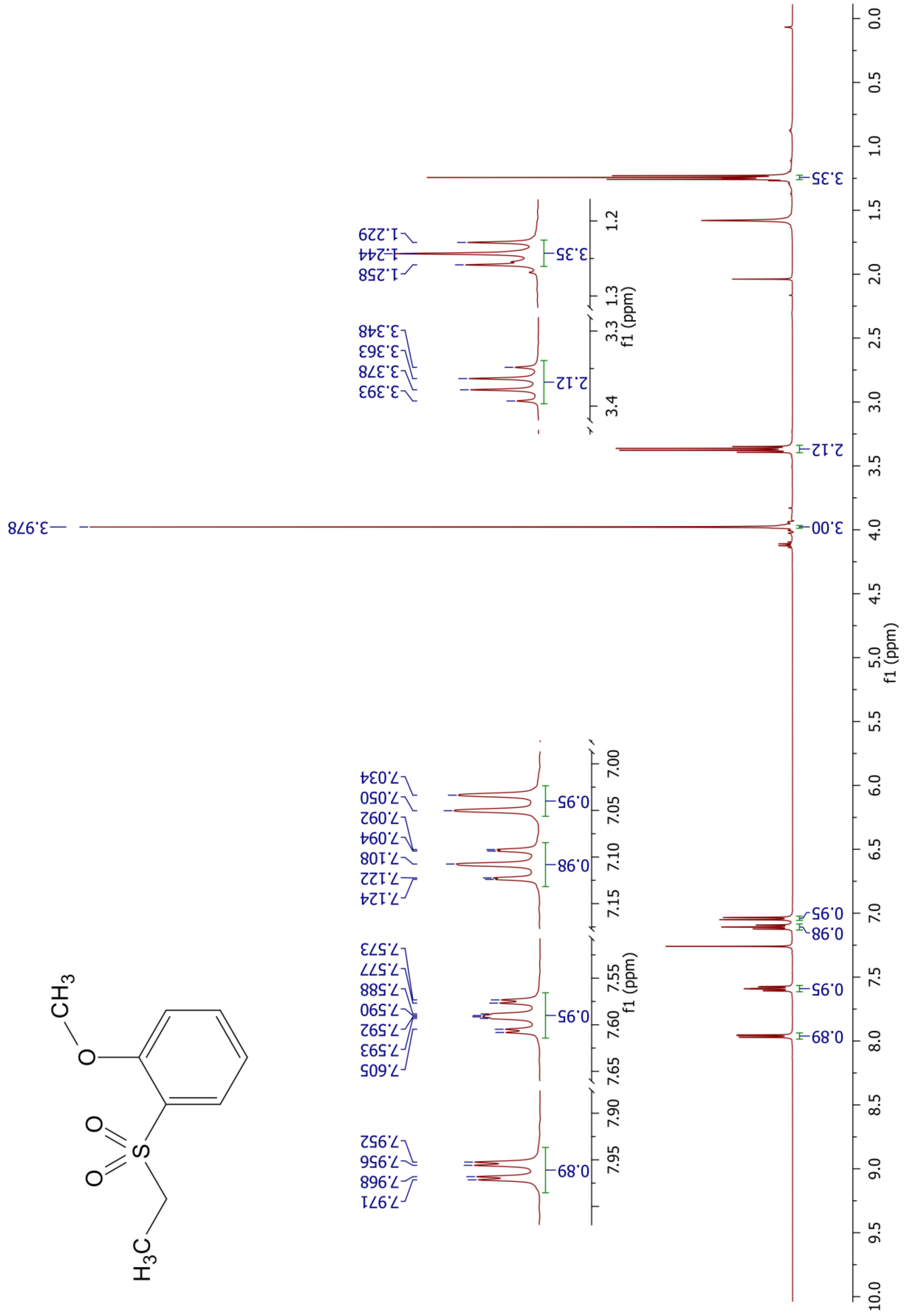

Figure S17. ^{1}H-NMR of 1-(ethylsulfonyl)-2-methoxybenzene (**3j’**)

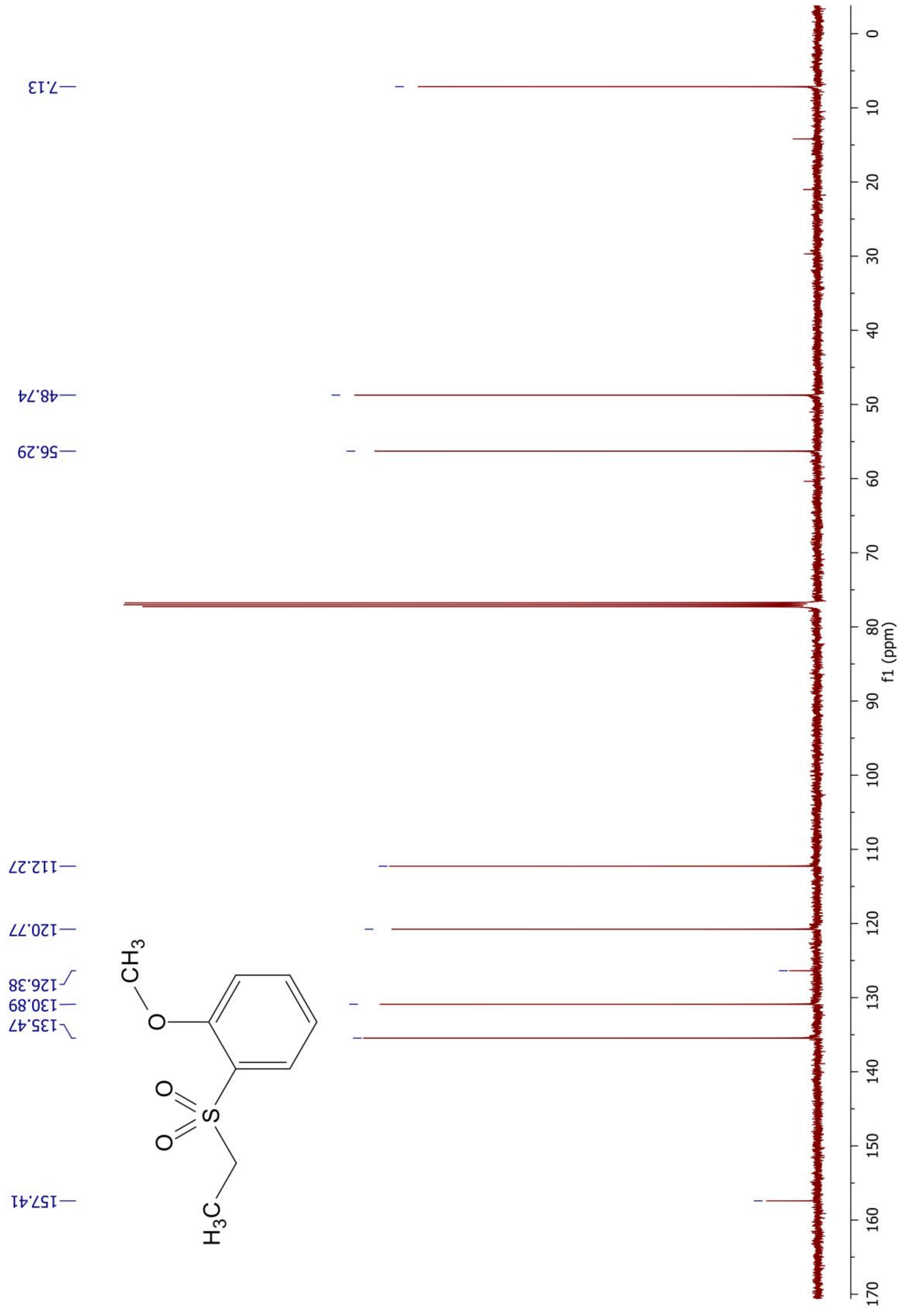

Figure S18. ^{13}C-NMR of 1-(ethylsulfonyl)-2-methoxybenzene (**3j'**)

molecules

MDPI

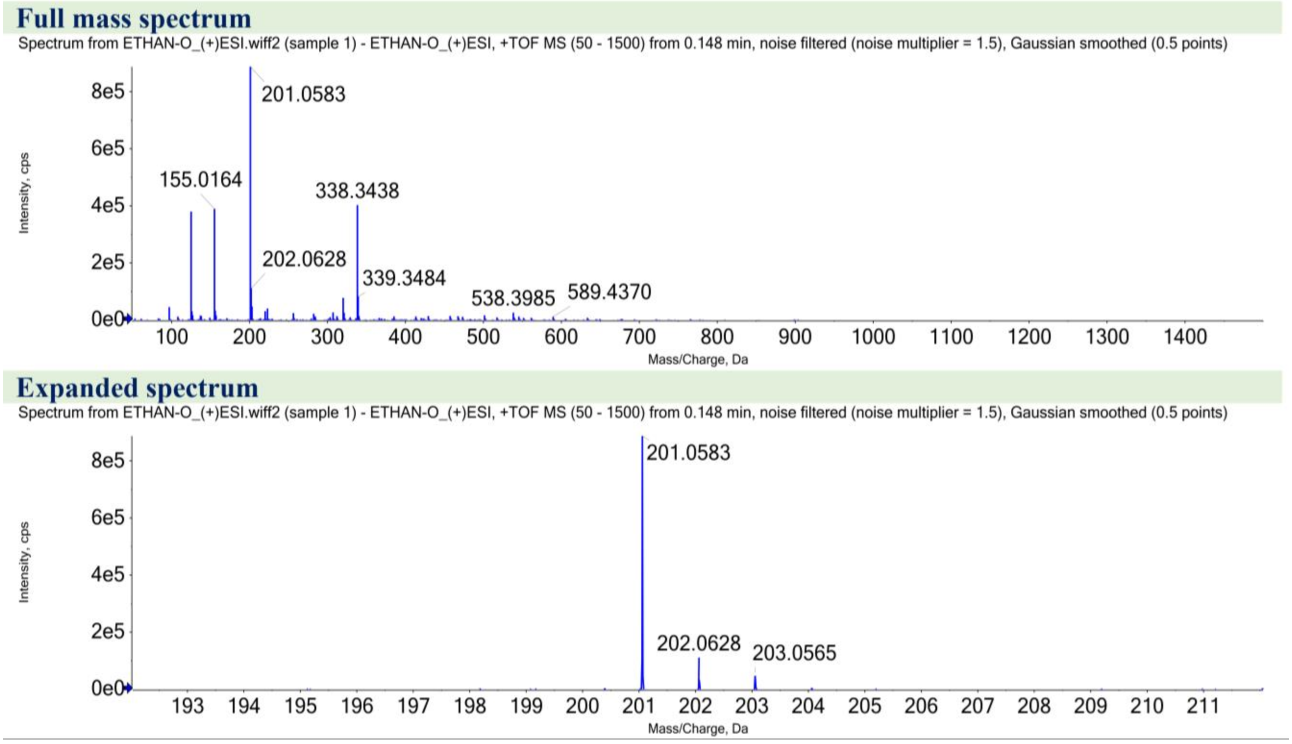

Figure S19. HRMS of 1-(ethylsulfonyl)-2-methoxybenzene (**3j'**)

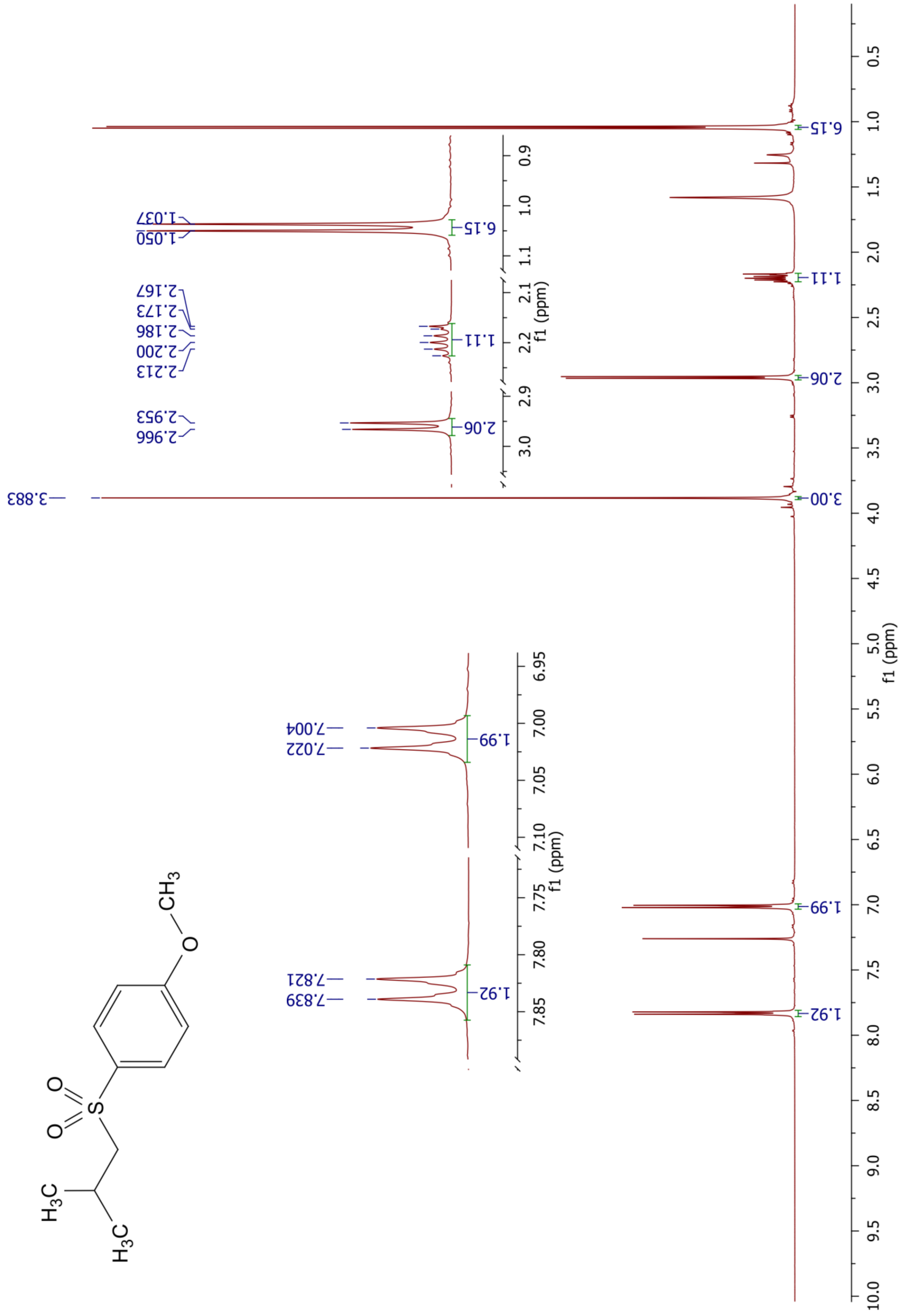

Figure S20. ^{1}H-NMR of 1-(isobutylsulfonyl)-4-methoxybenzene (**3k**)

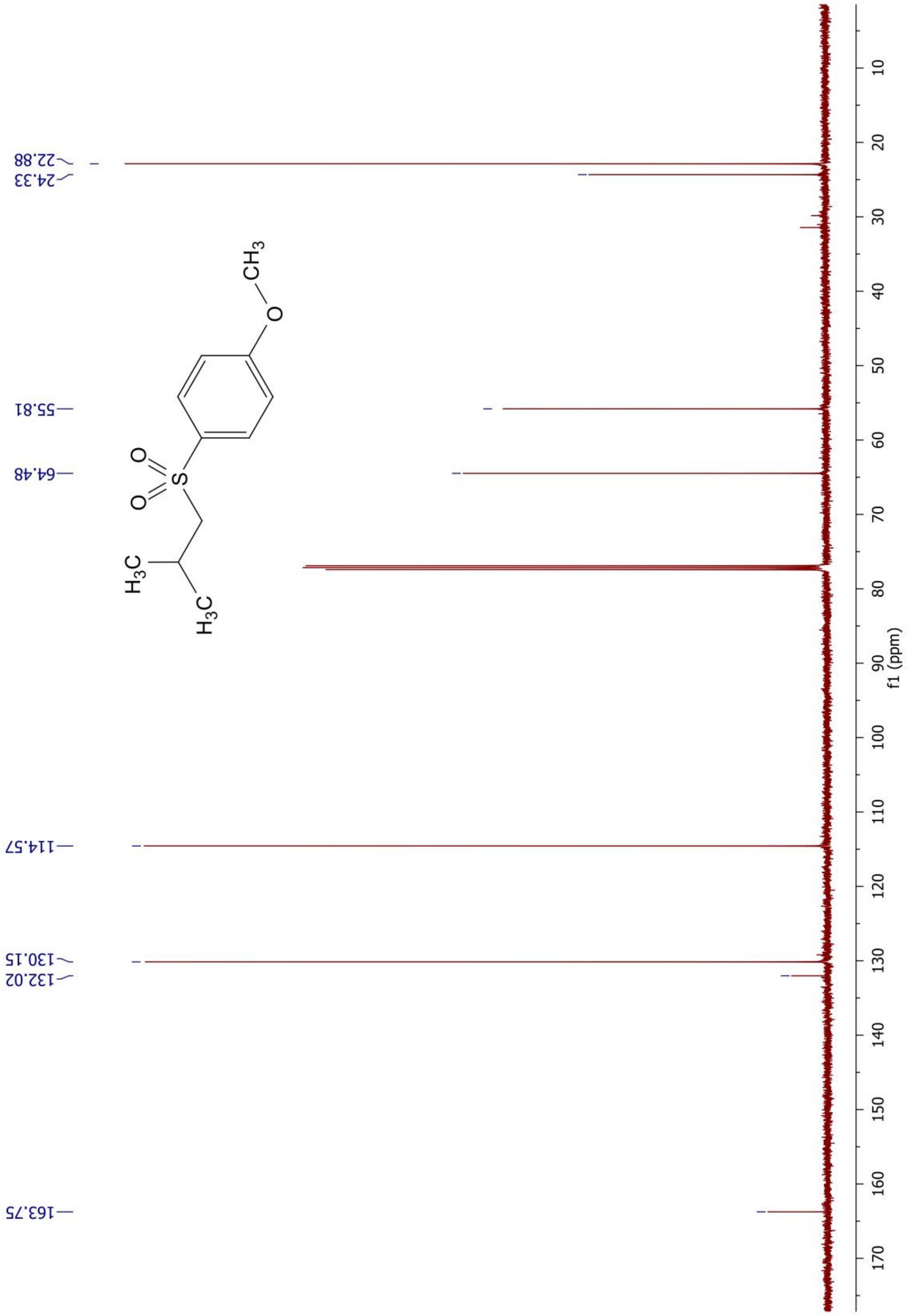

Figure S21. ^{13}C-NMR of 1-(isobutylsulfonyl)-4-methoxybenzene (**3k**)

Full mass spectrum

Spectrum from ISB-P_(+)ESI.wiff2 (sample 1) - ISB-P_(+)ESI, +TOF MS (50 - 1500) from 0.153 min, noise filtered (noise multiplier = 1.5), Gaussian smoothed (0.5 points)

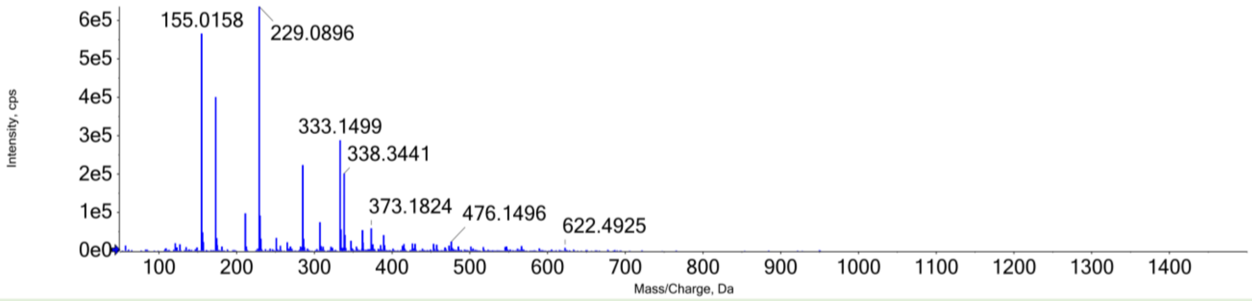

Expanded spectrum

Spectrum from ISB-P_(+)ESI.wiff2 (sample 1) - ISB-P_(+)ESI, +TOF MS (50 - 1500) from 0.153 min, noise filtered (noise multiplier = 1.5), Gaussian smoothed (0.5 points)

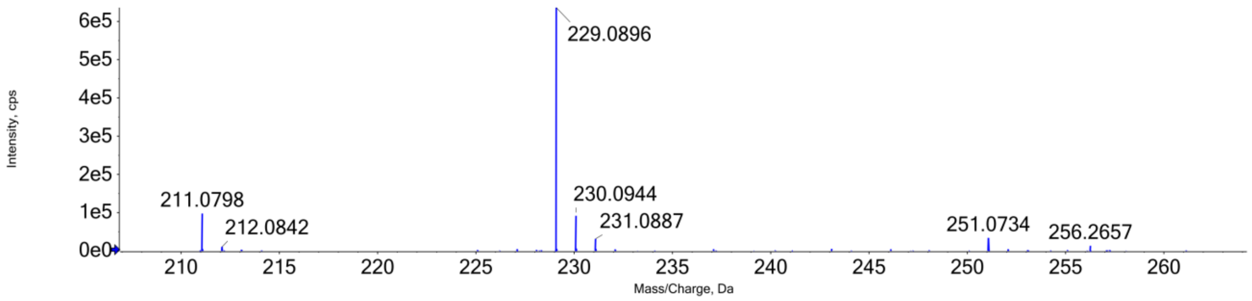

Figure S22. HRMS of 1-(isobutylsulfonyl)-4-methoxybenzene (**3k**)

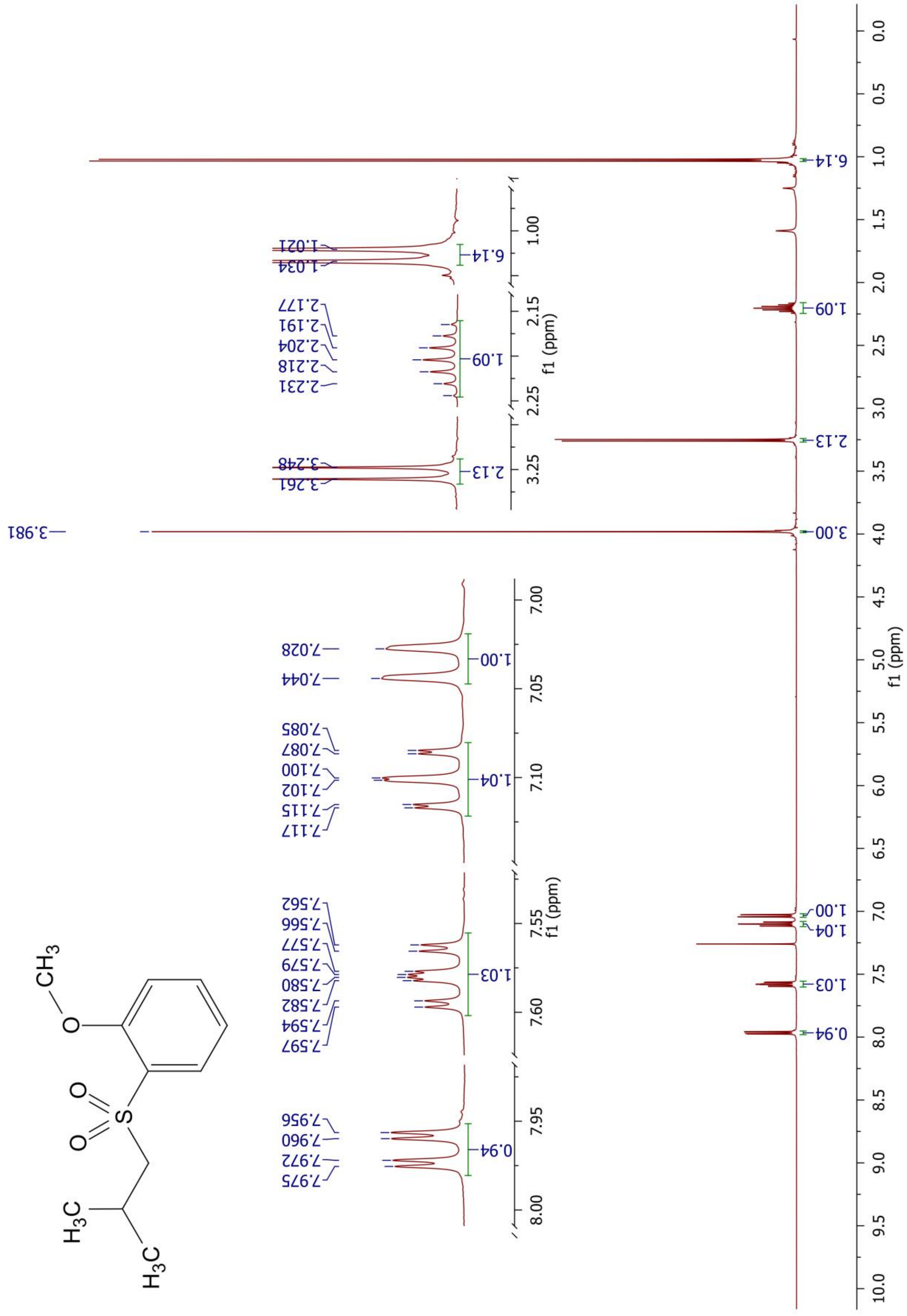

Figure S23. ^{1}H-NMR of 1-(isobutylsulfonyl)-2-methoxybenzene (**3k'**)

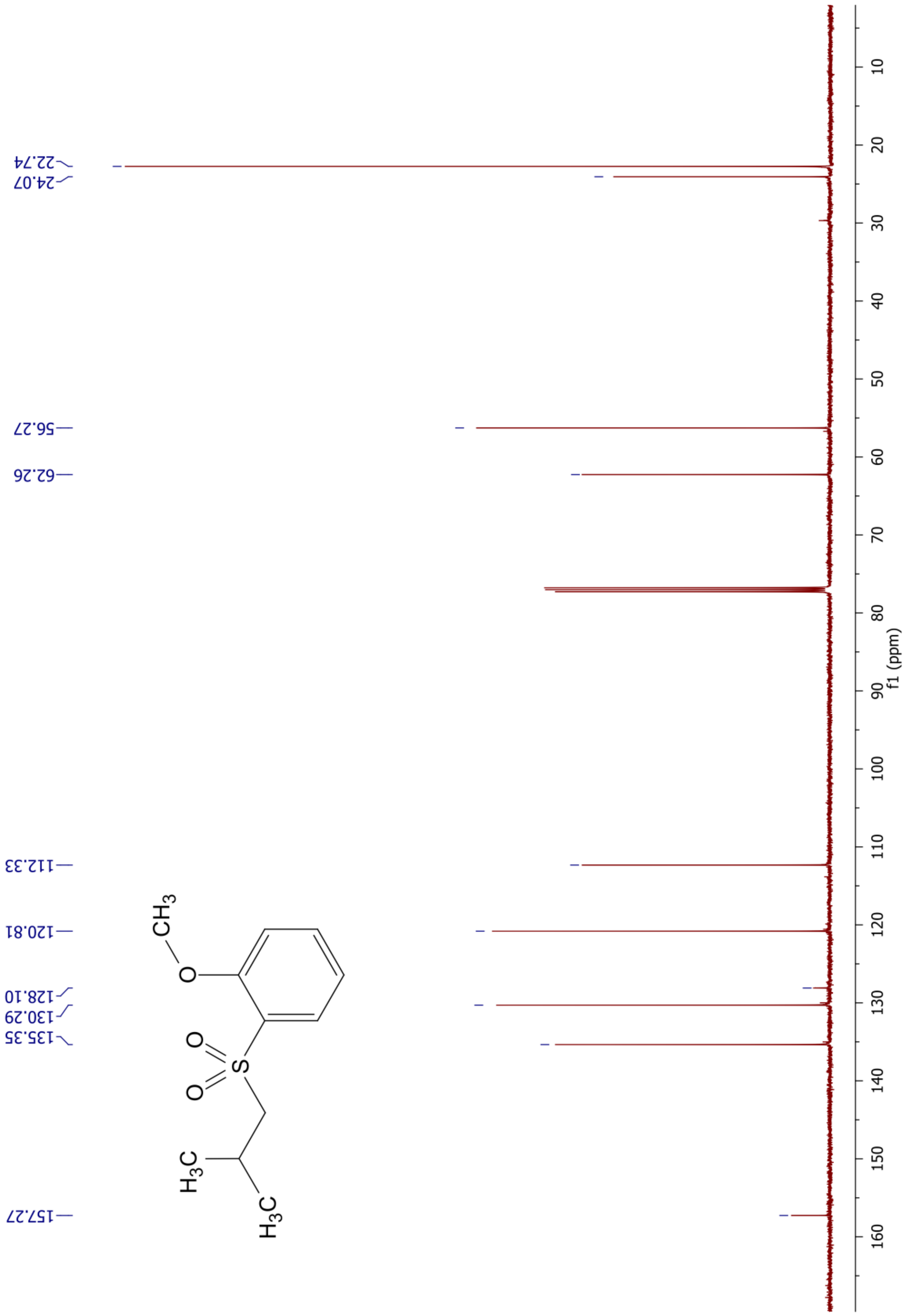

Figure S24. ^{13}C-NMR of 1-(isobutylsulfonyl)-2-methoxybenzene (**3k'**)

Full mass spectrum

Spectrum from ISB-O_(+)ESI.wiff2 (sample 1) - ISB-O_(+)ESI, +TOF MS (50 - 1500) from 0.143 min, noise filtered (noise multiplier = 1.5), Gaussian smoothed (0.5 points)

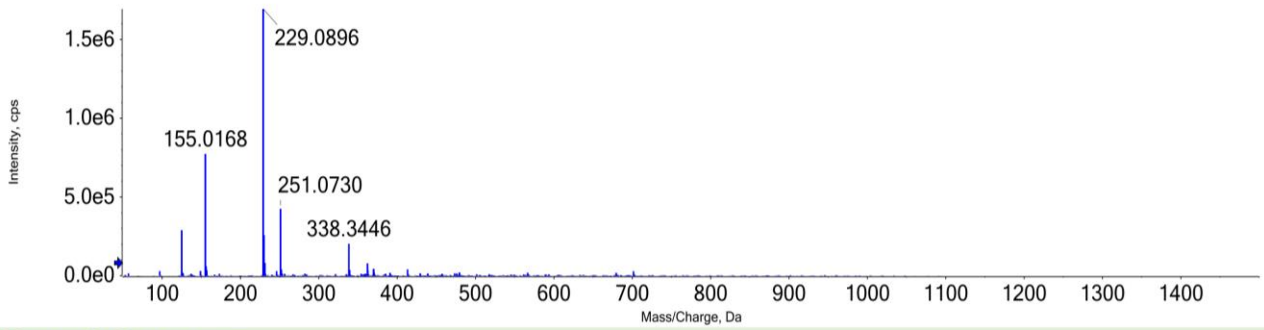

Expanded spectrum

Spectrum from ISB-O_(+)ESI.wiff2 (sample 1) - ISB-O_(+)ESI, +TOF MS (50 - 1500) from 0.143 min, noise filtered (noise multiplier = 1.5), Gaussian smoothed (0.5 points)

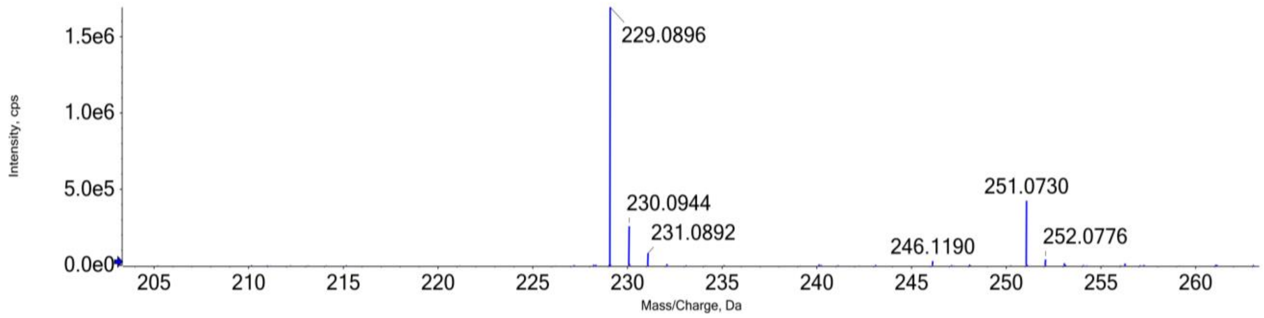

Figure S25. HRMS of 1-(isobutylsulfonyl)-2-methoxybenzene (**3k'**)

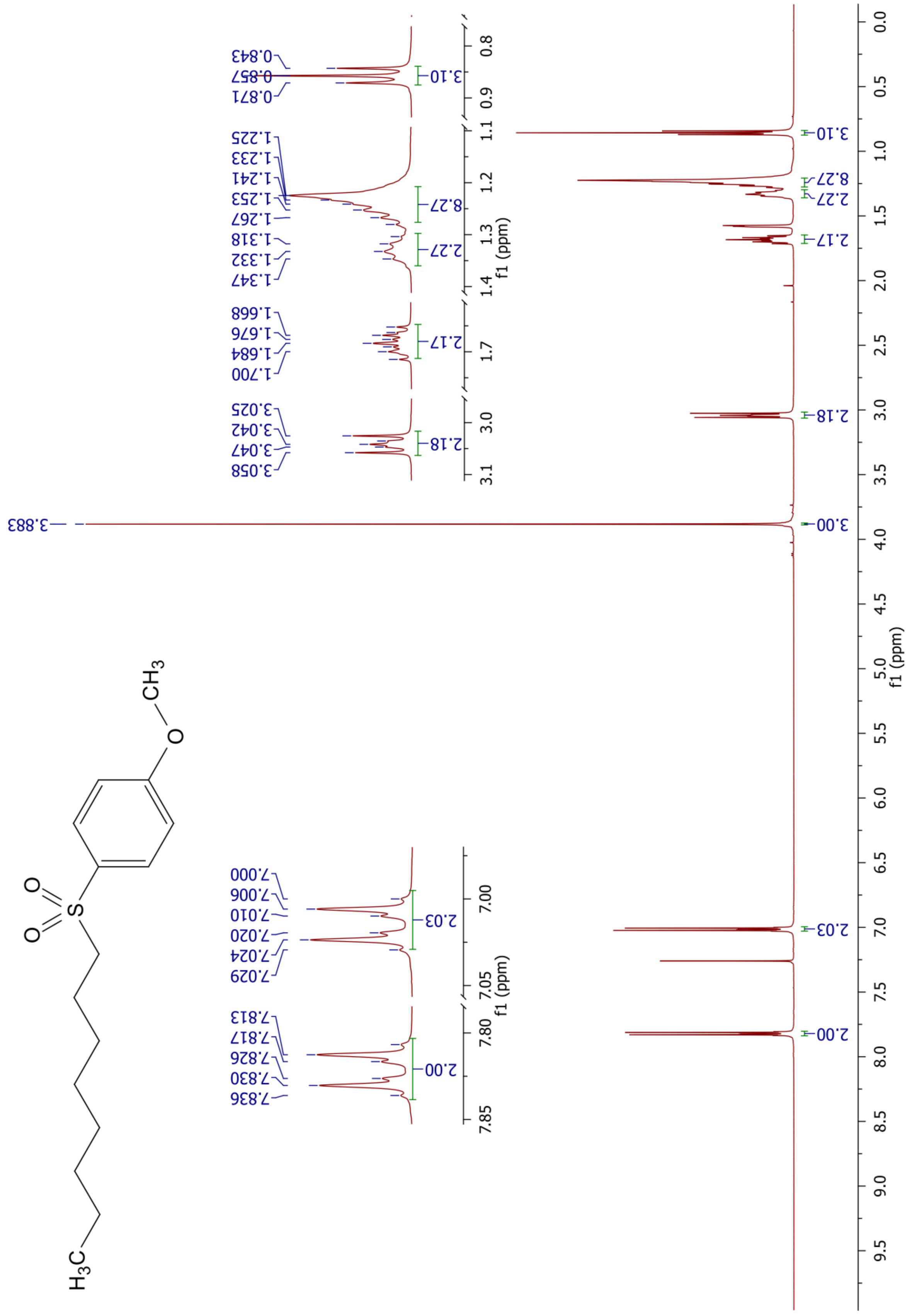

Figure S26. ^{1}H-NMR of 1-methoxy-4-(octylsulfonyl)benzene (**3l**)

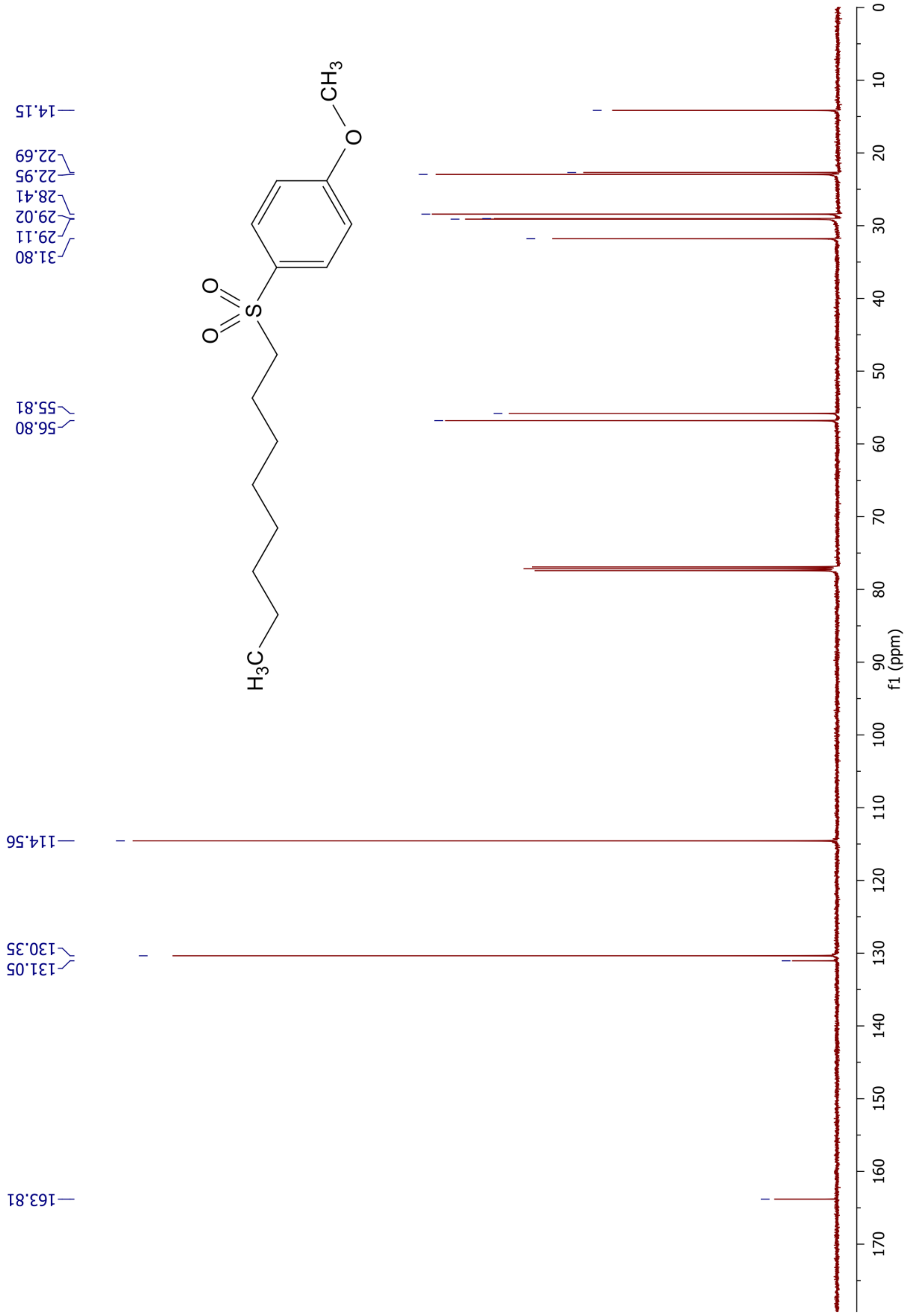

Figure S27. ^{13}C-NMR of 1-methoxy-4-(octylsulfonyl)benzene (**3l**)

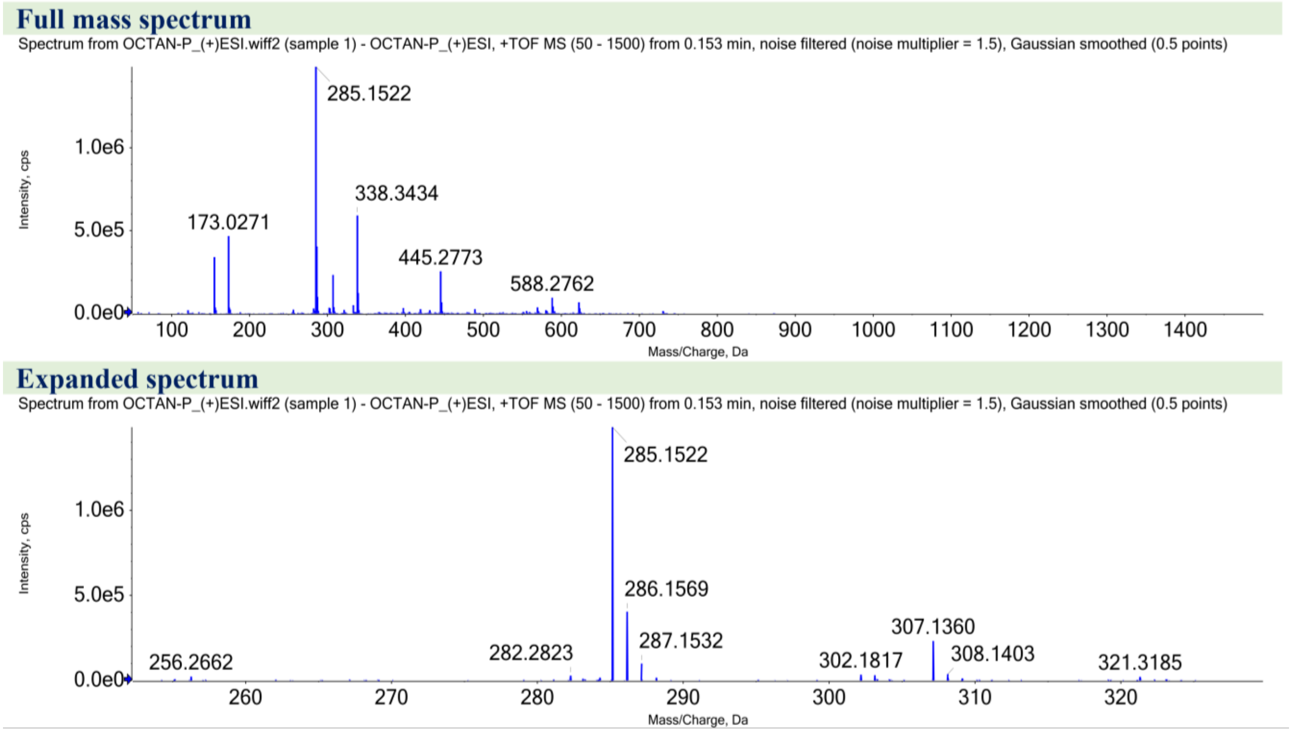

Figure S28. HRMS of 1-methoxy-4-(octylsulfonyl)benzene (**3l**)

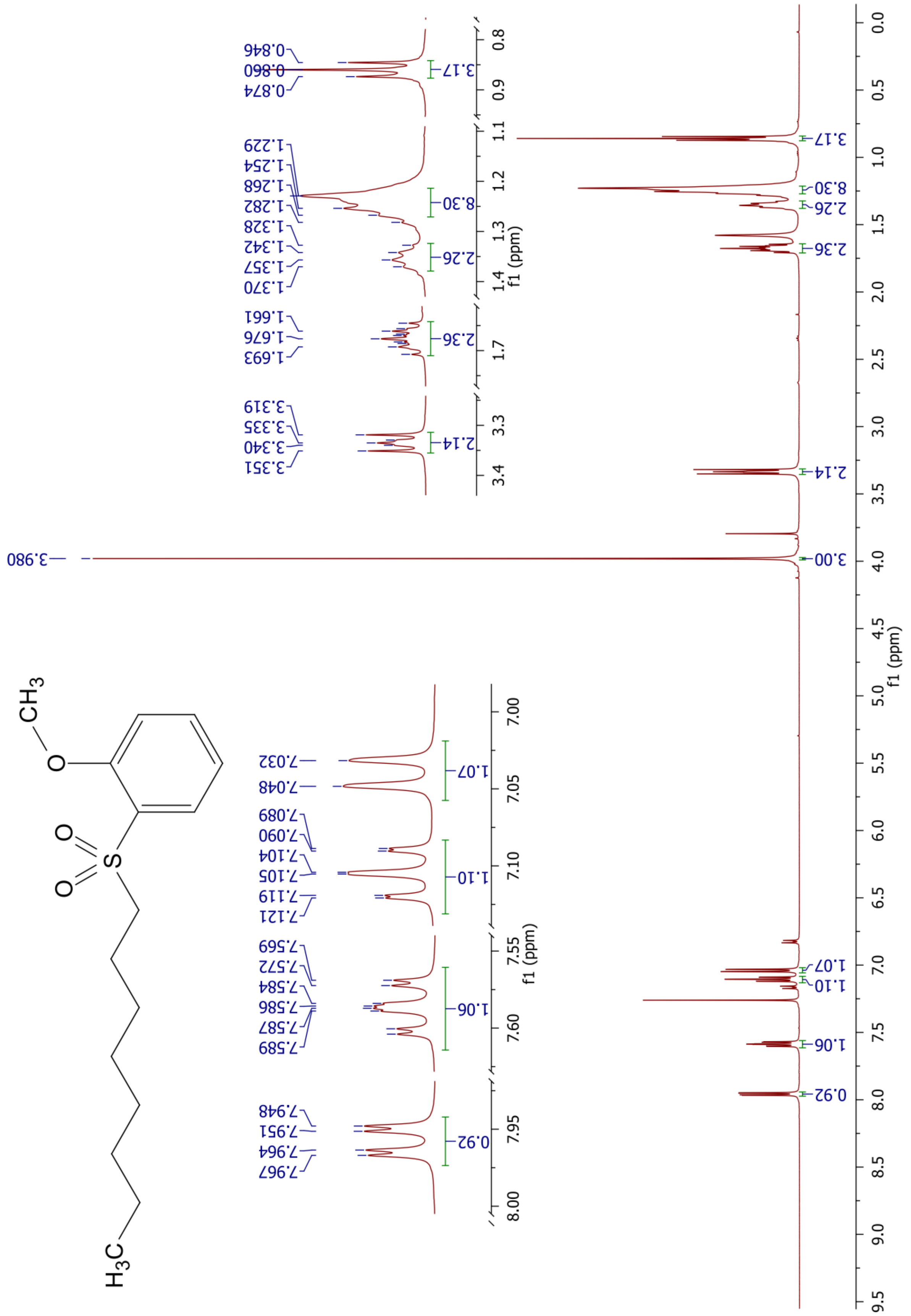

Figure S29. ^{1}H-NMR of 1-methoxy-2-(octylsulfonyl)benzene (**3l'**)

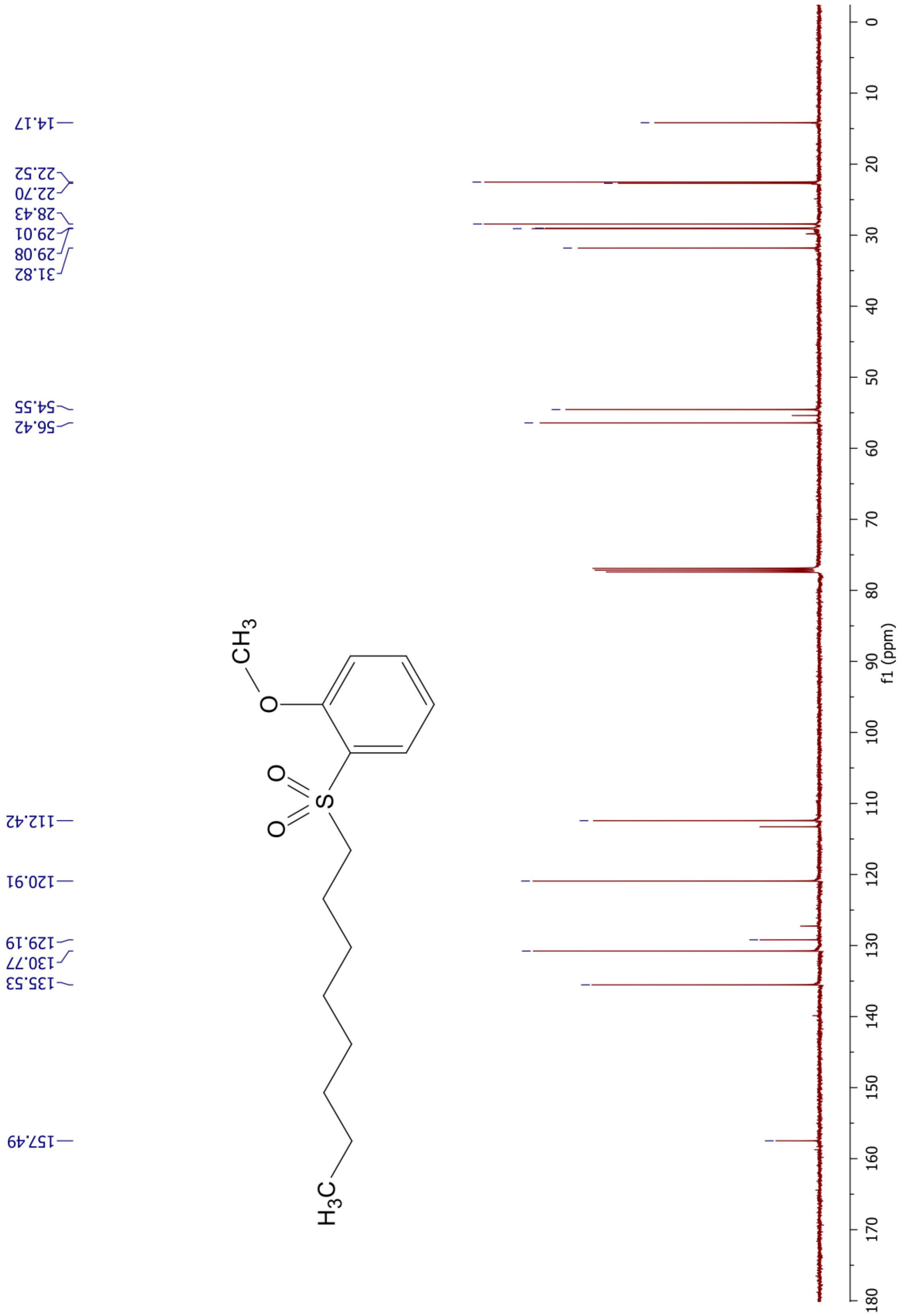

Figure S30. ^{13}C-NMR of 1-methoxy-2-(octylsulfonyl)benzene (**3l'**)

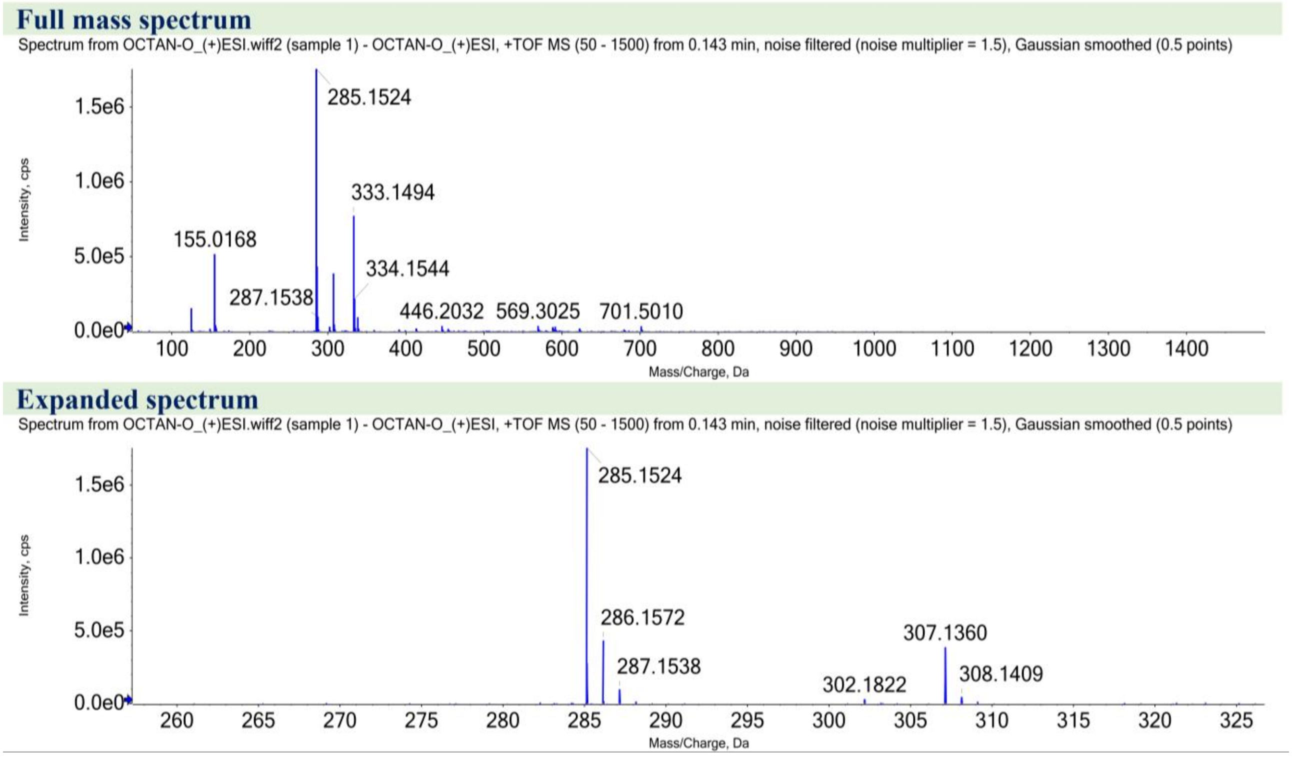

Figure S31. HRMS of 1-methoxy-2-(octylsulfonyl)benzene (**3l'**)

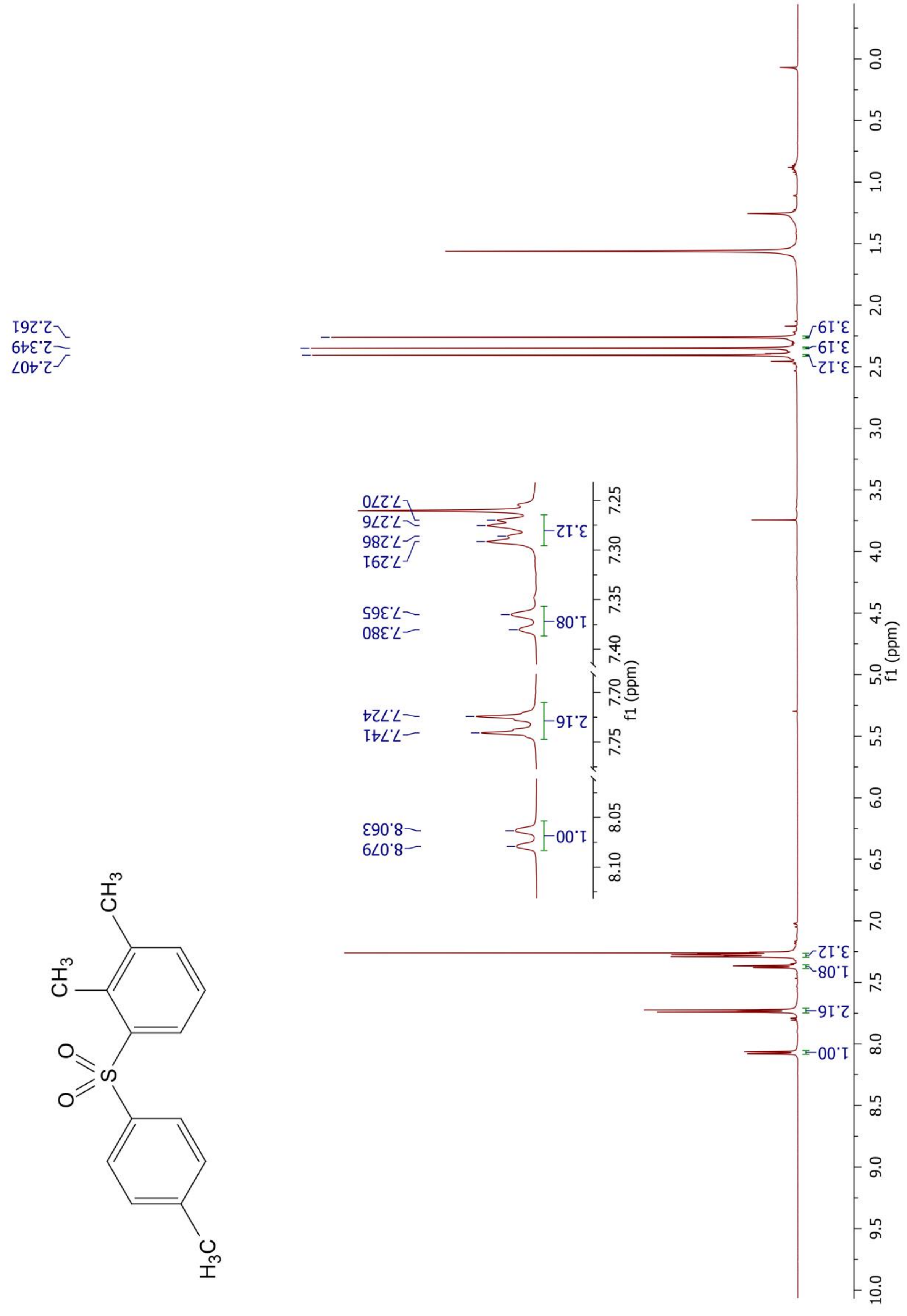

Figure S32: ^{1}H-NMR of 2,3-dimethyl-1-tosylbenzene (**3o’**)

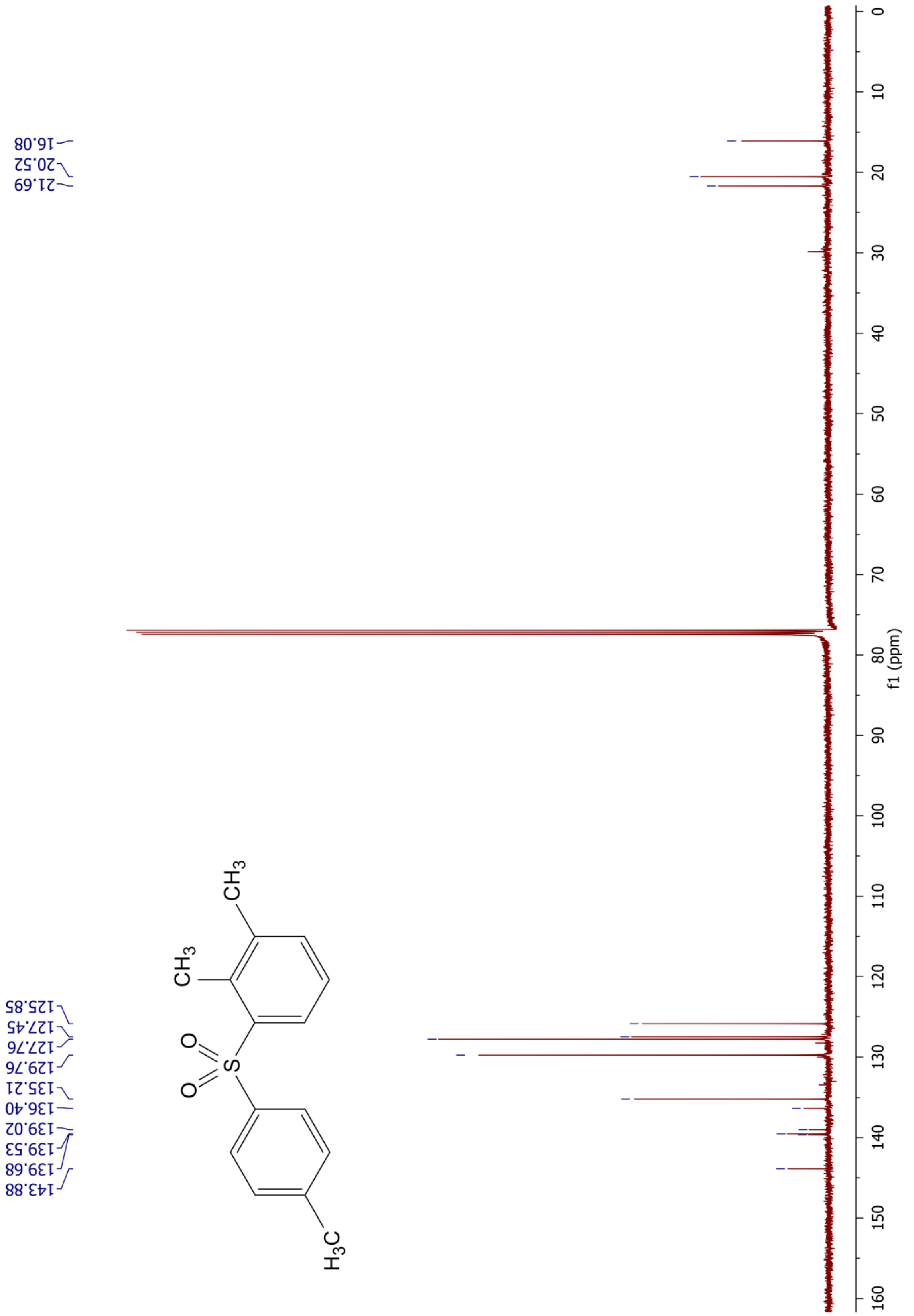

Figure S33: ^{13}C-NMR of 2,3-dimethyl-1-tosylbenzene (**3o'**)

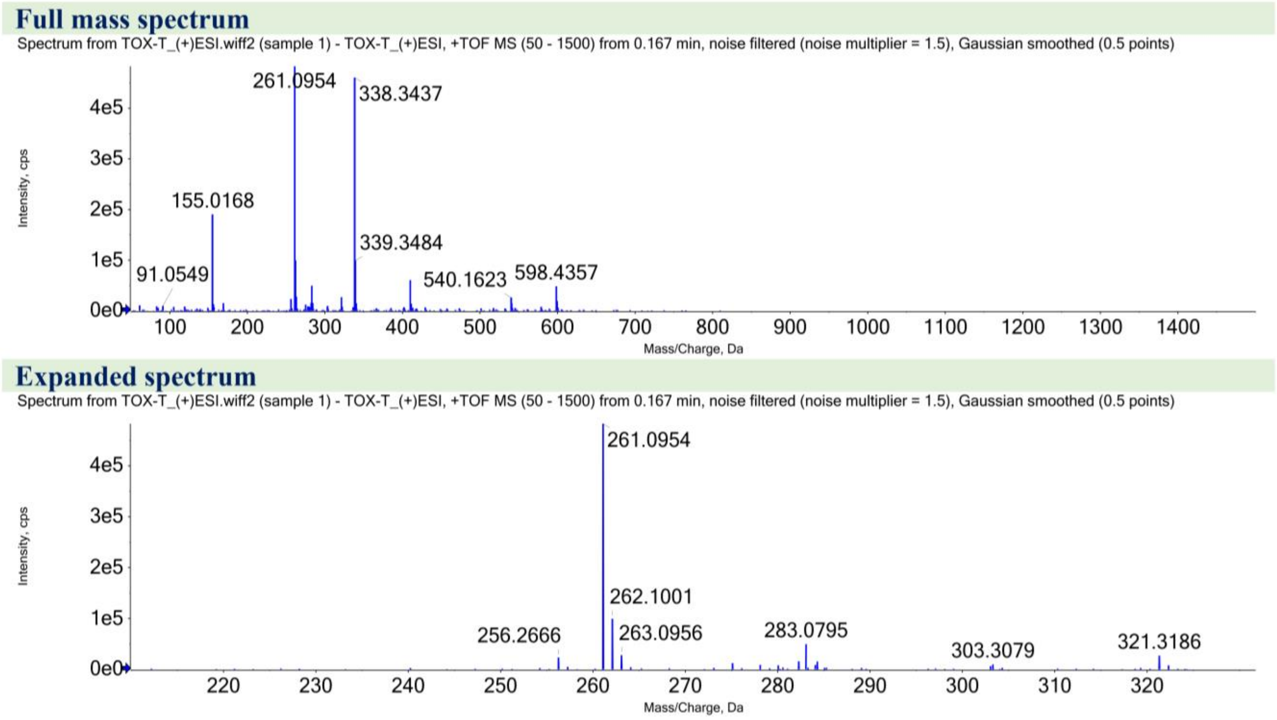

Figure S34: HRMS of 2,3-dimethyl-1-tosylbenzene (**3o'**)

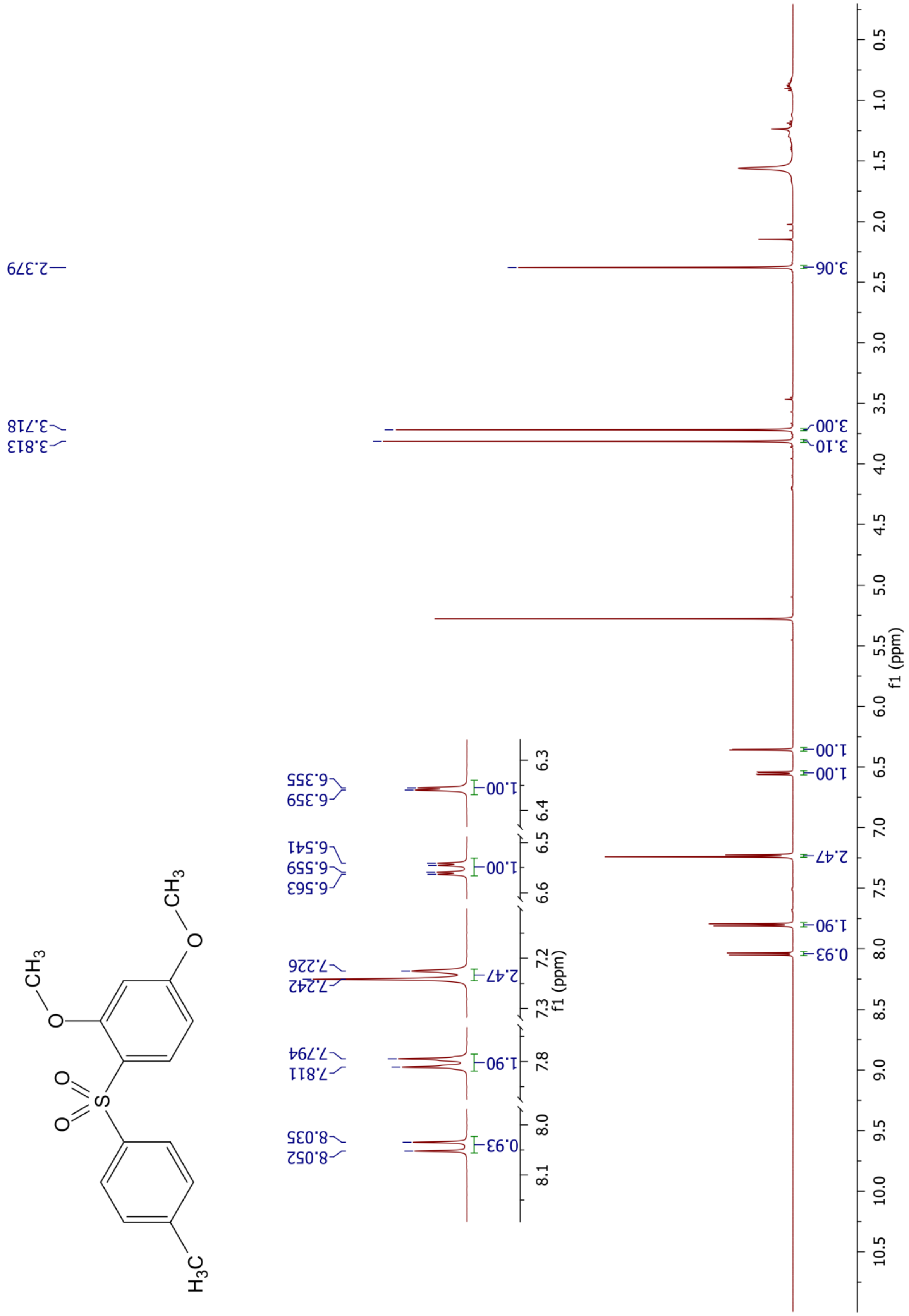

Figure S35. ^{1}H-NMR of 2,4-dimethoxy-1-tosylbenzene (**3p**)

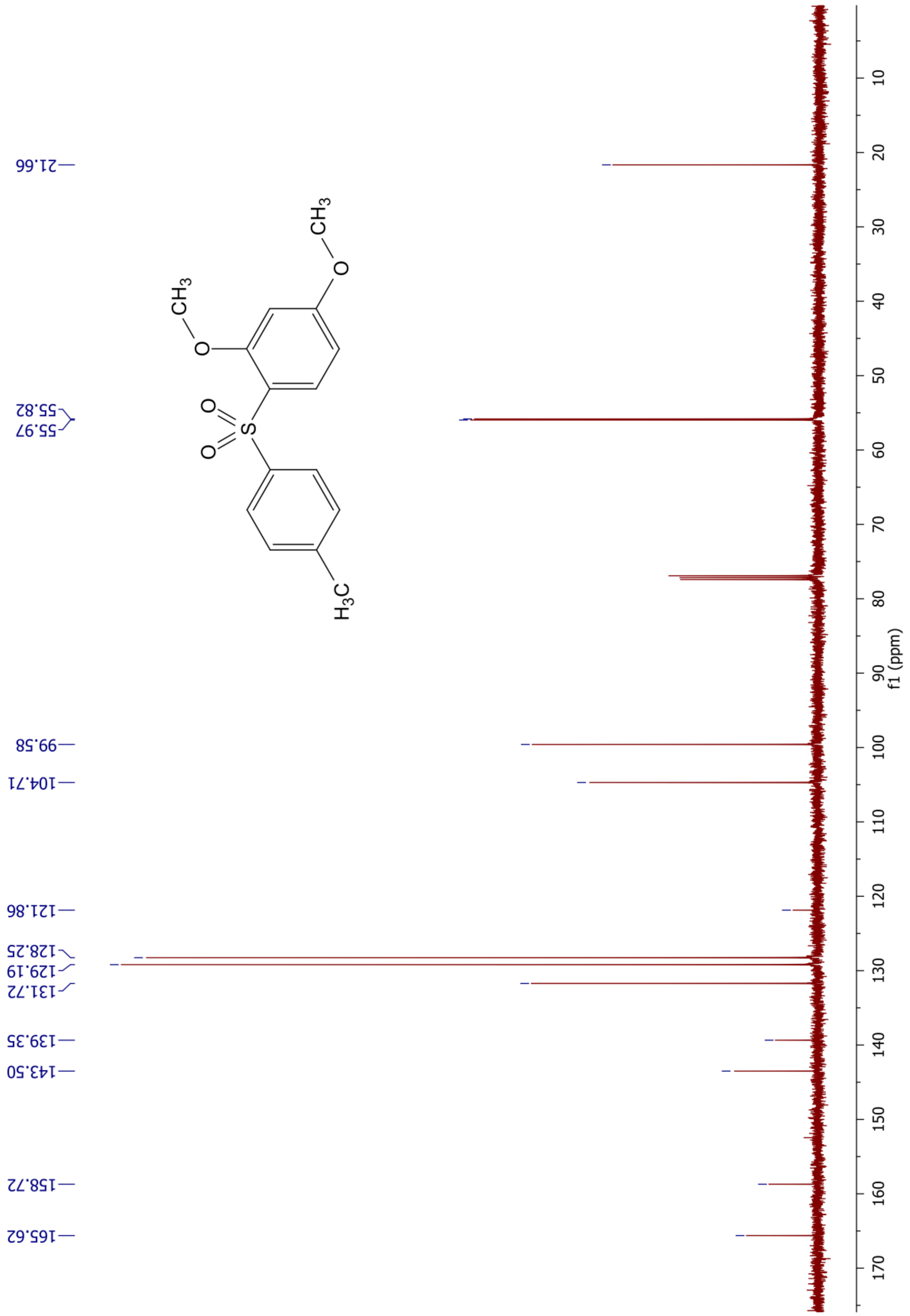

Figure S36. ^{13}C-NMR of 2,4-dimethoxy-1-tosylbenzene (**3p**)

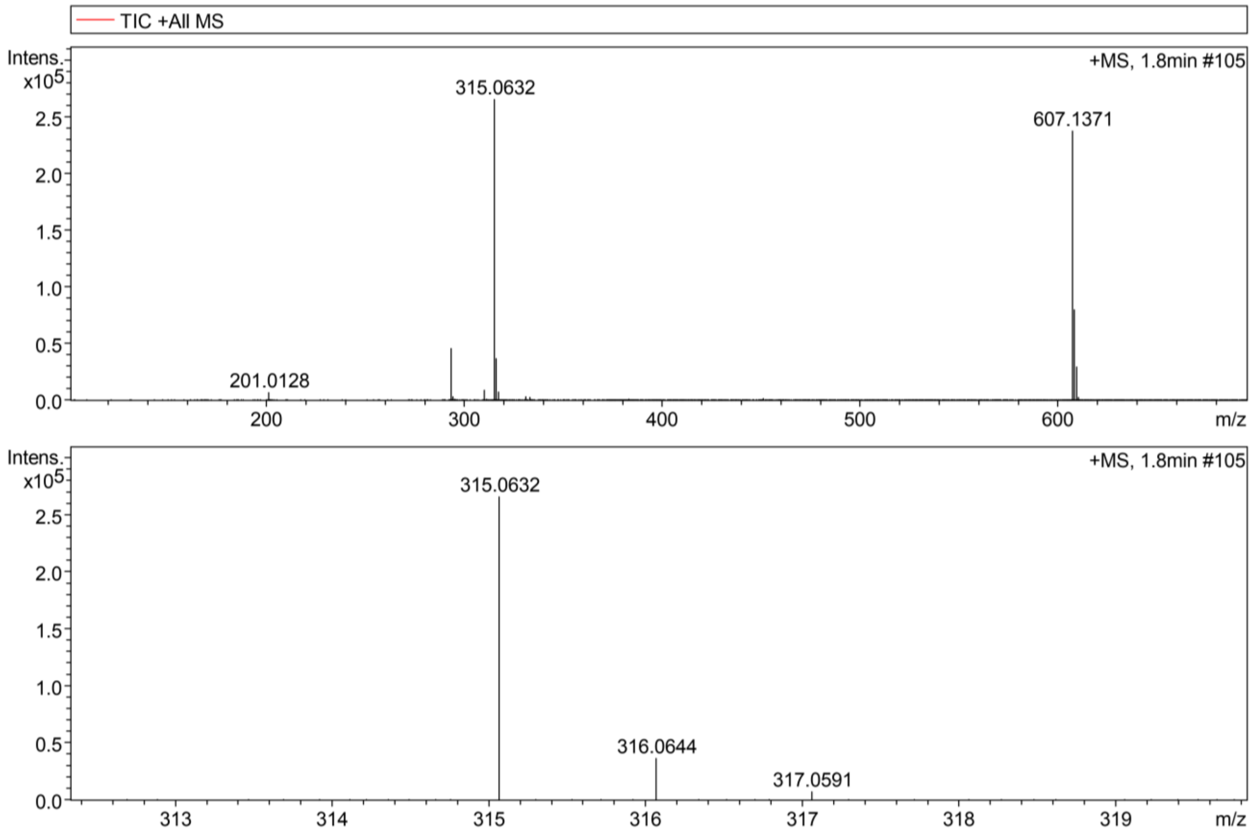

Figure S37. HRMS of 2,4-dimethoxy-1-tosylbenzene (**3p**)

molecules

MDPI

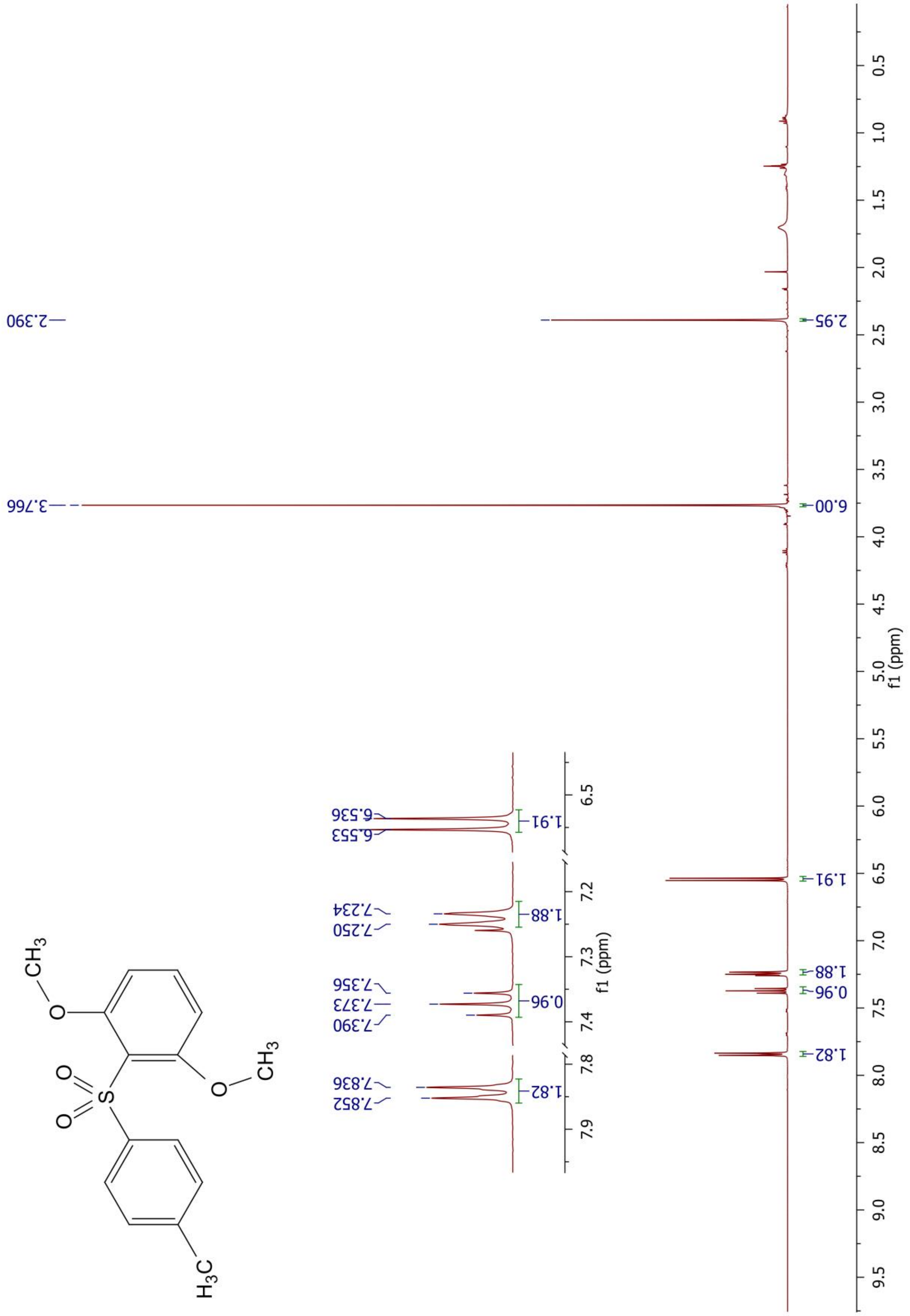

Figure S38. ^{1}H-NMR of 1,3-dimethoxy-2-tosylbenzene (**3p'**)

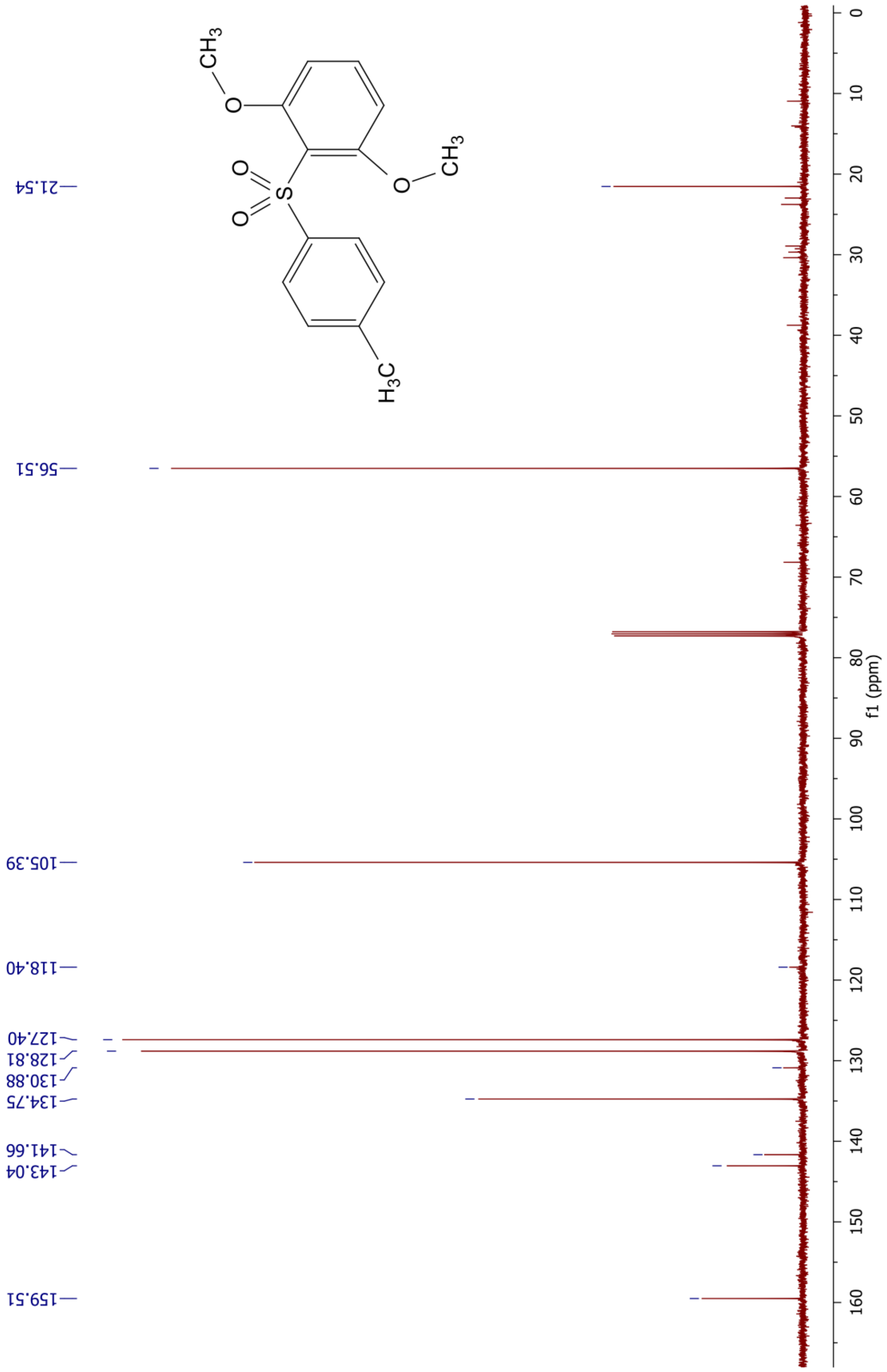

Figure S39. ^{13}C-NMR of 1,3-dimethoxy-2-tosylbenzene (**3p’**)

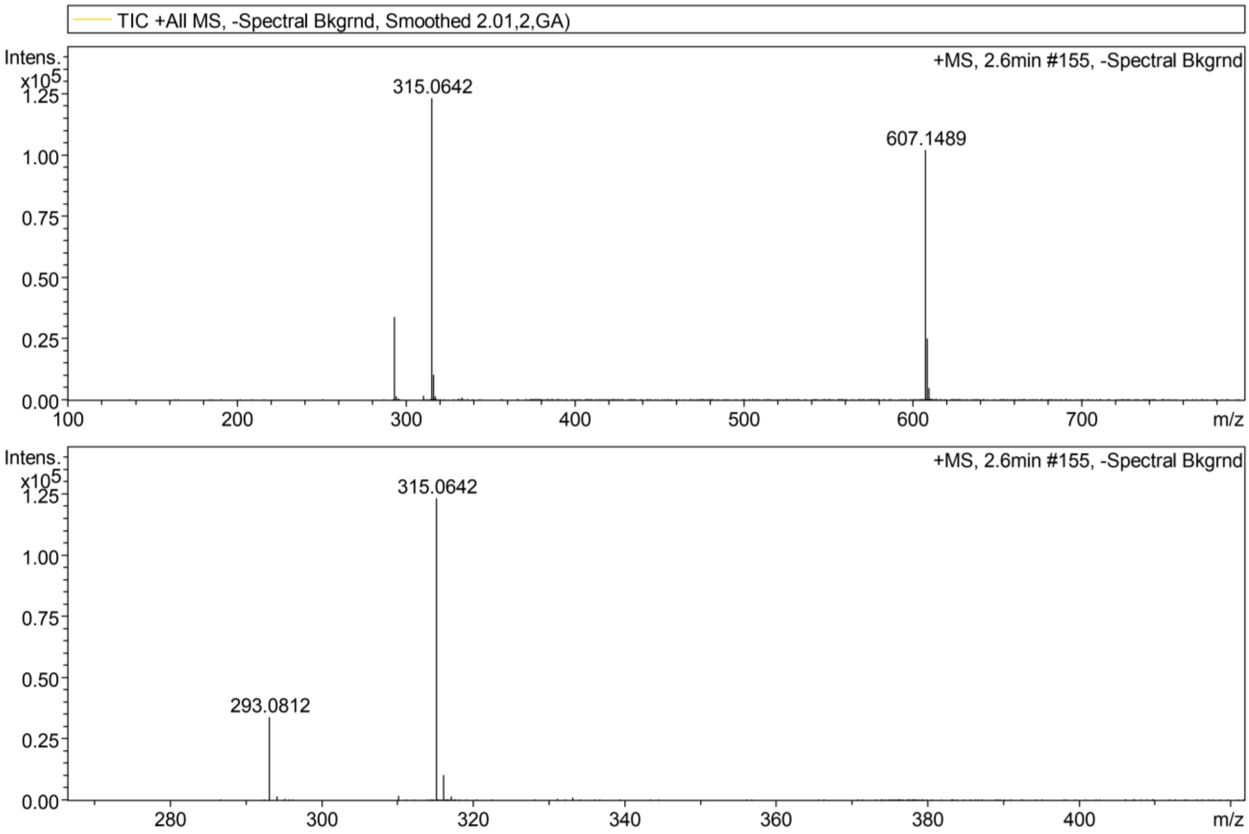

Figure S40. HRMS of 1,3-dimethoxy-2-tosylbenzene (**3p'**)

molecules

MDPI

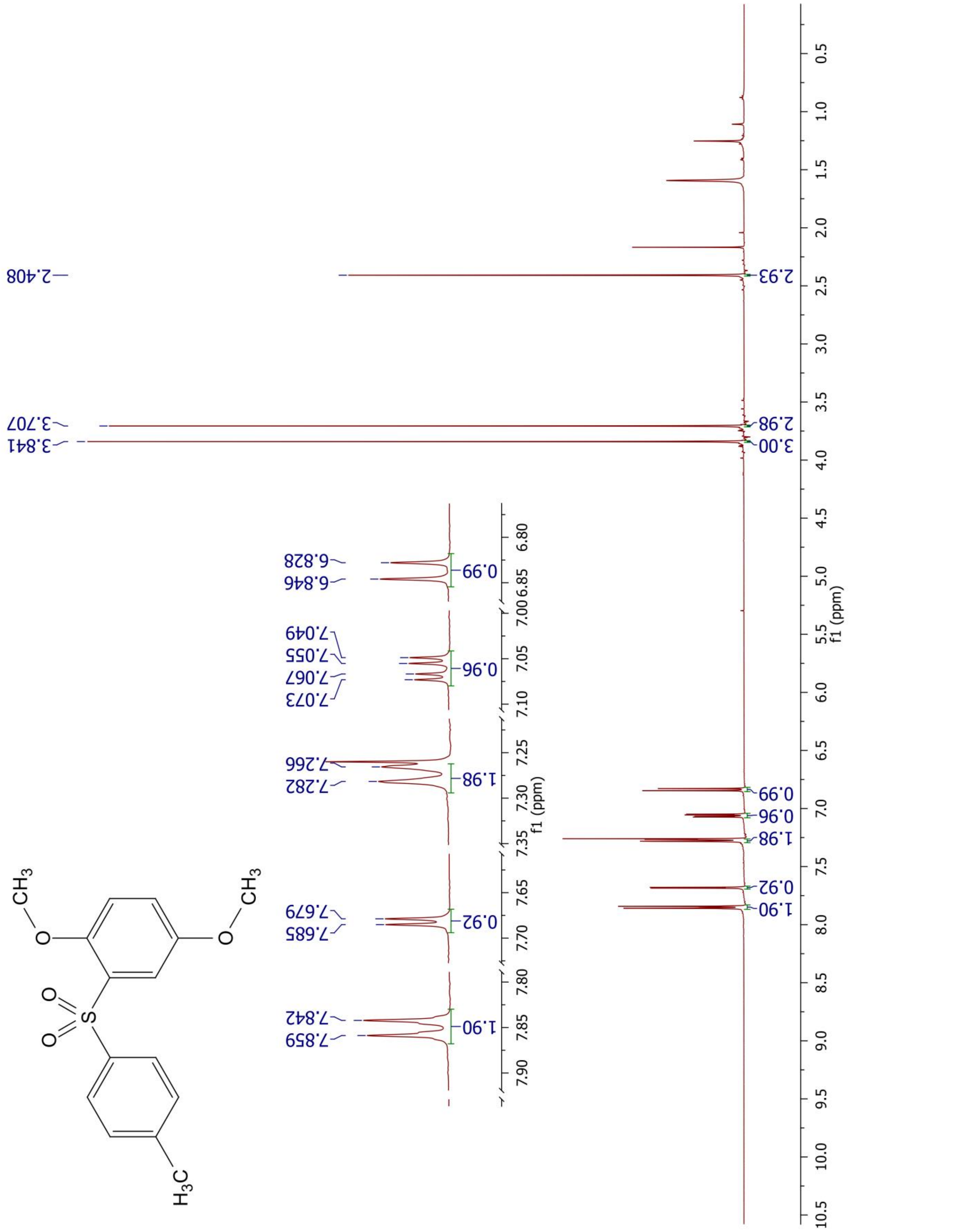

Figure S41. ^{1}H-NMR of 1,4-dimethoxy-2-tosylbenzene (**3q**)

molecules

MDPI

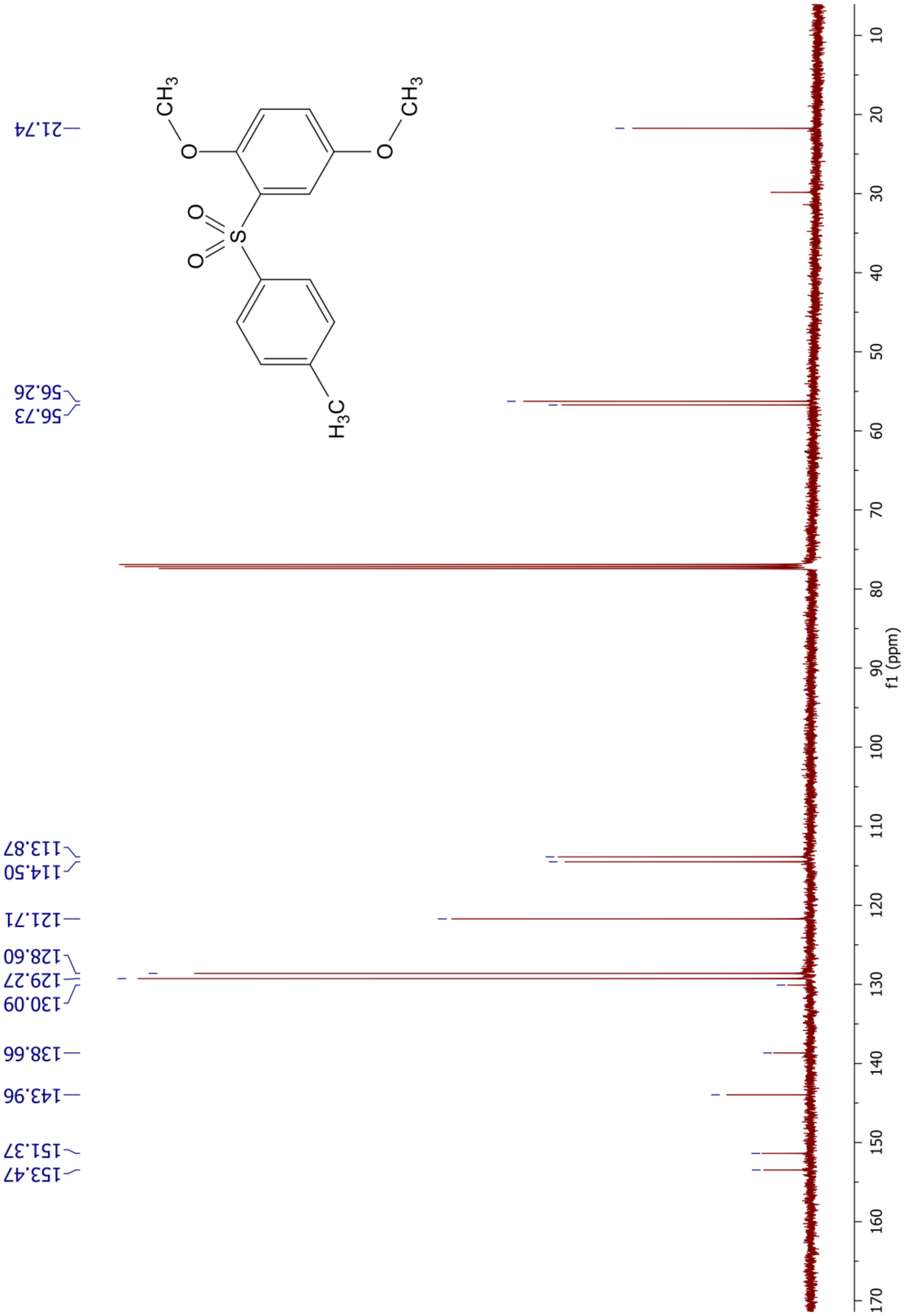

Figure S42. ^{13}C-NMR of 1,4-dimethoxy-2-tosylbenzene (**3q**)

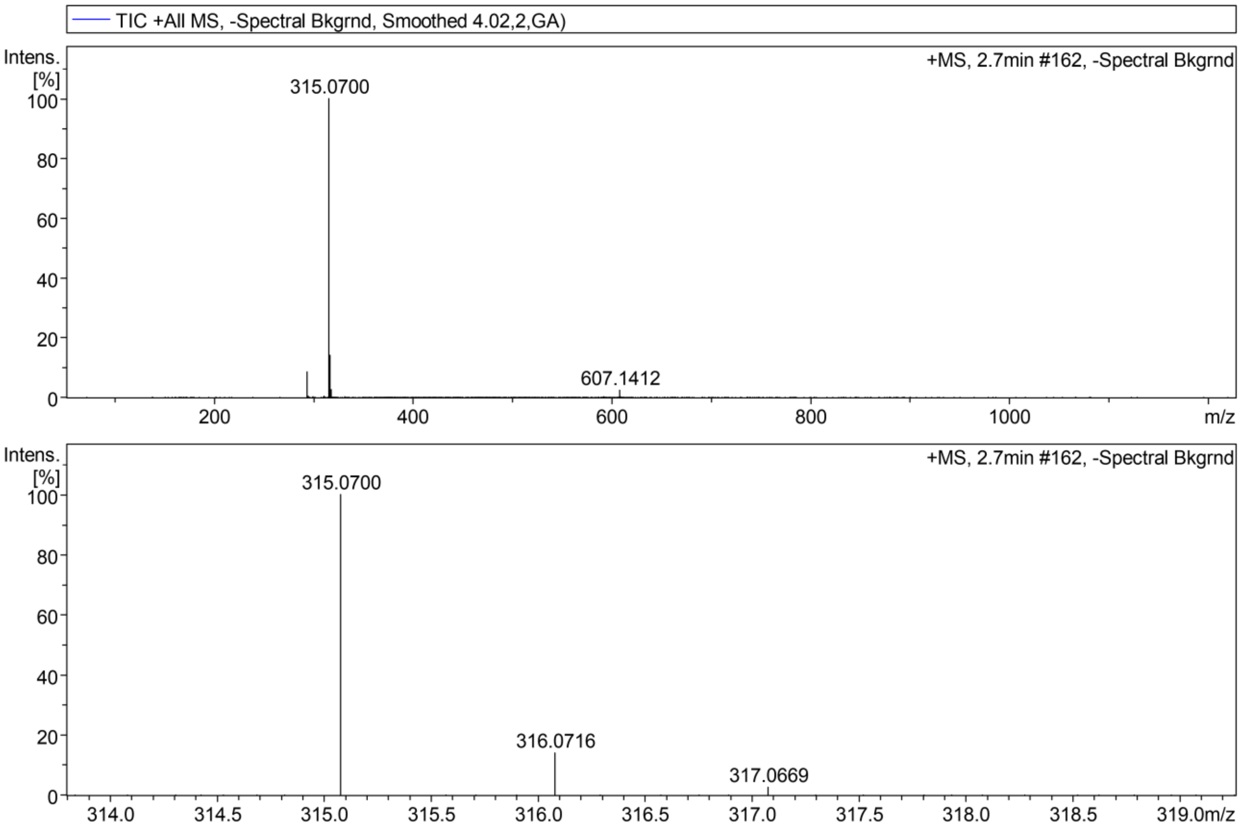

Figure S43. HRMS of 1,4-dimethoxy-2-tosylbenzene (**3q**)

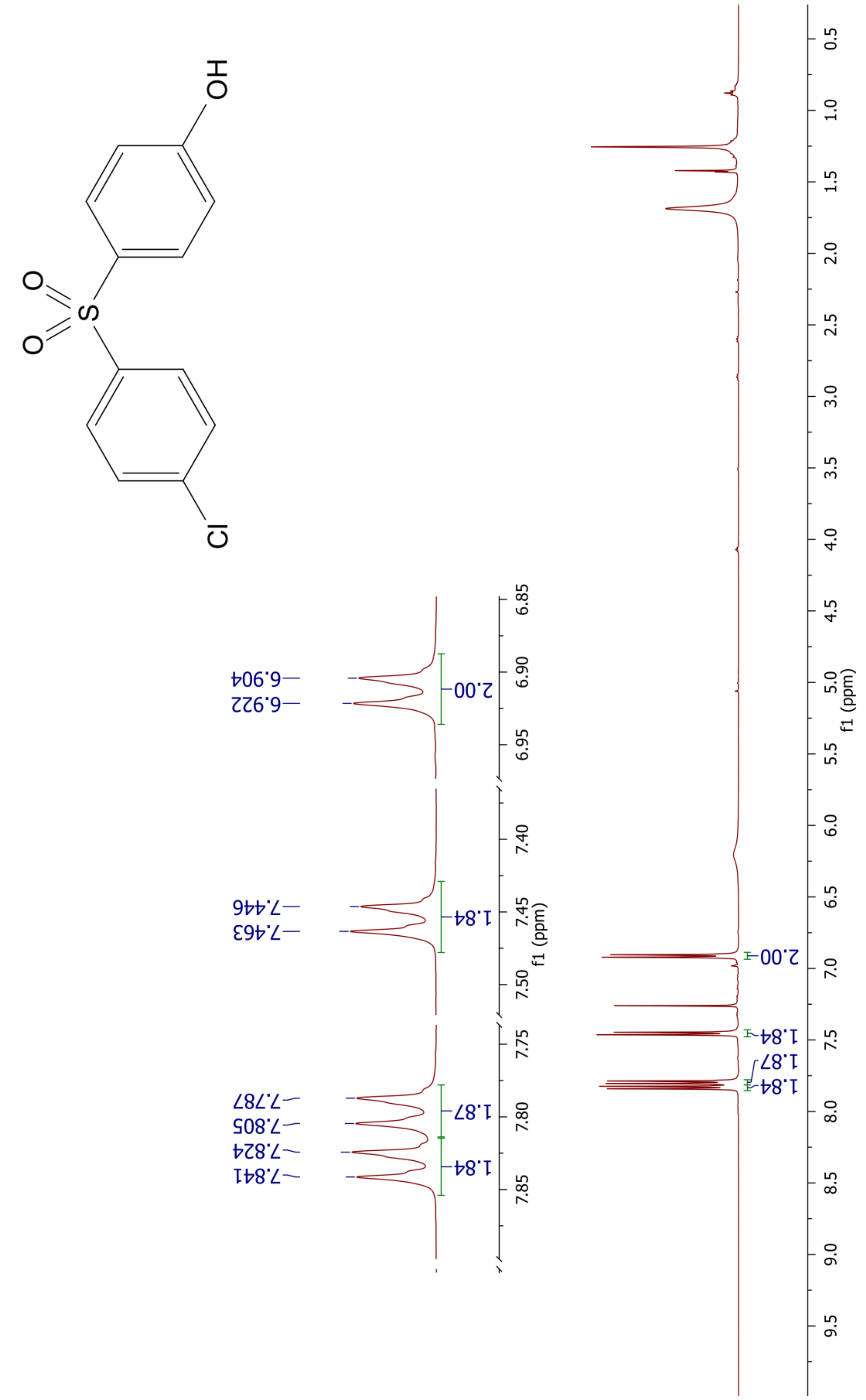

Figure S44. ^{1}H-NMR of 4-((4-chlorophenyl)sulfonyl)phenol (**3r**)

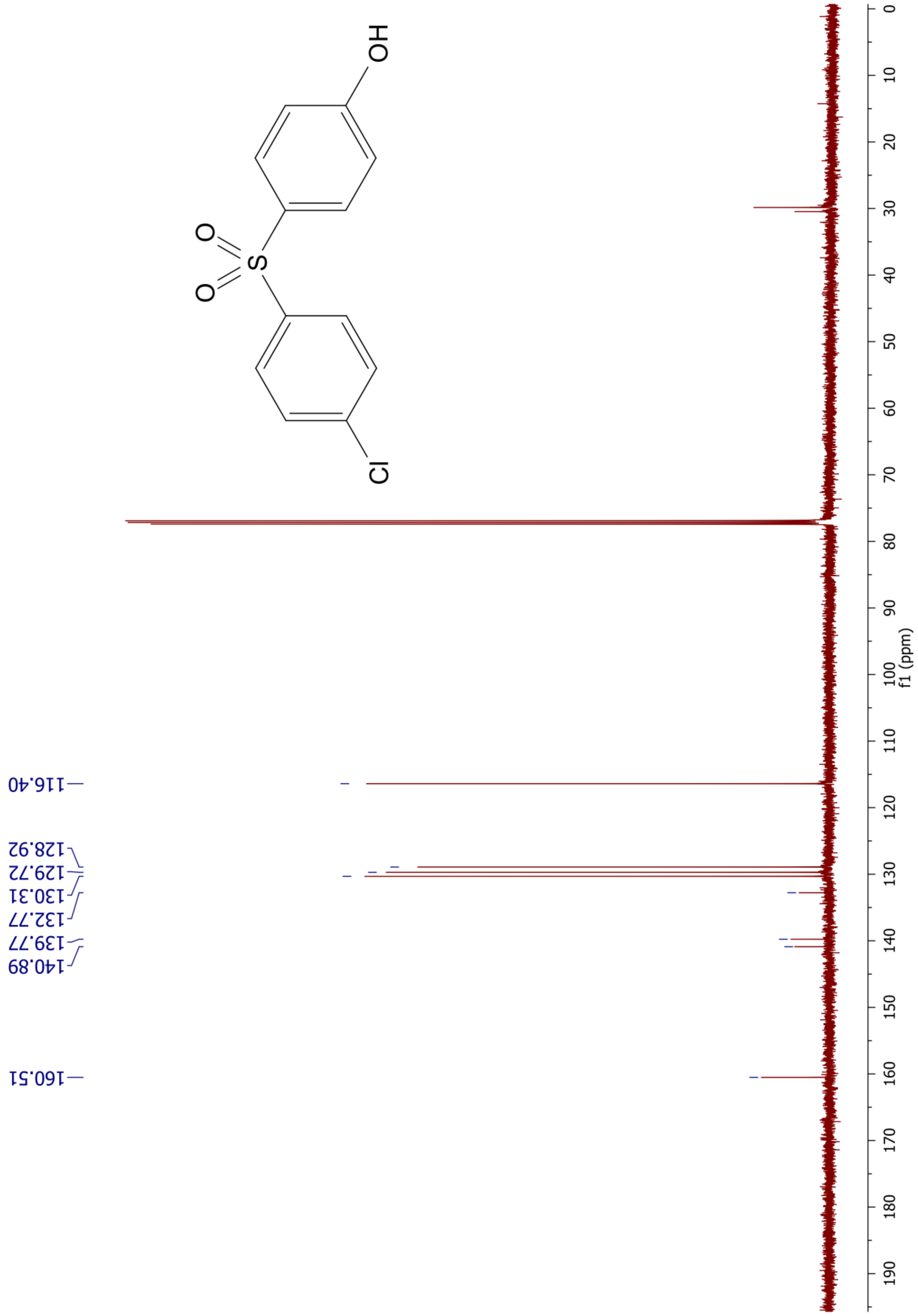

Figure S45. ^{13}C-NMR of 4-((4-chlorophenyl)sulfonyl)phenol (**3r**)

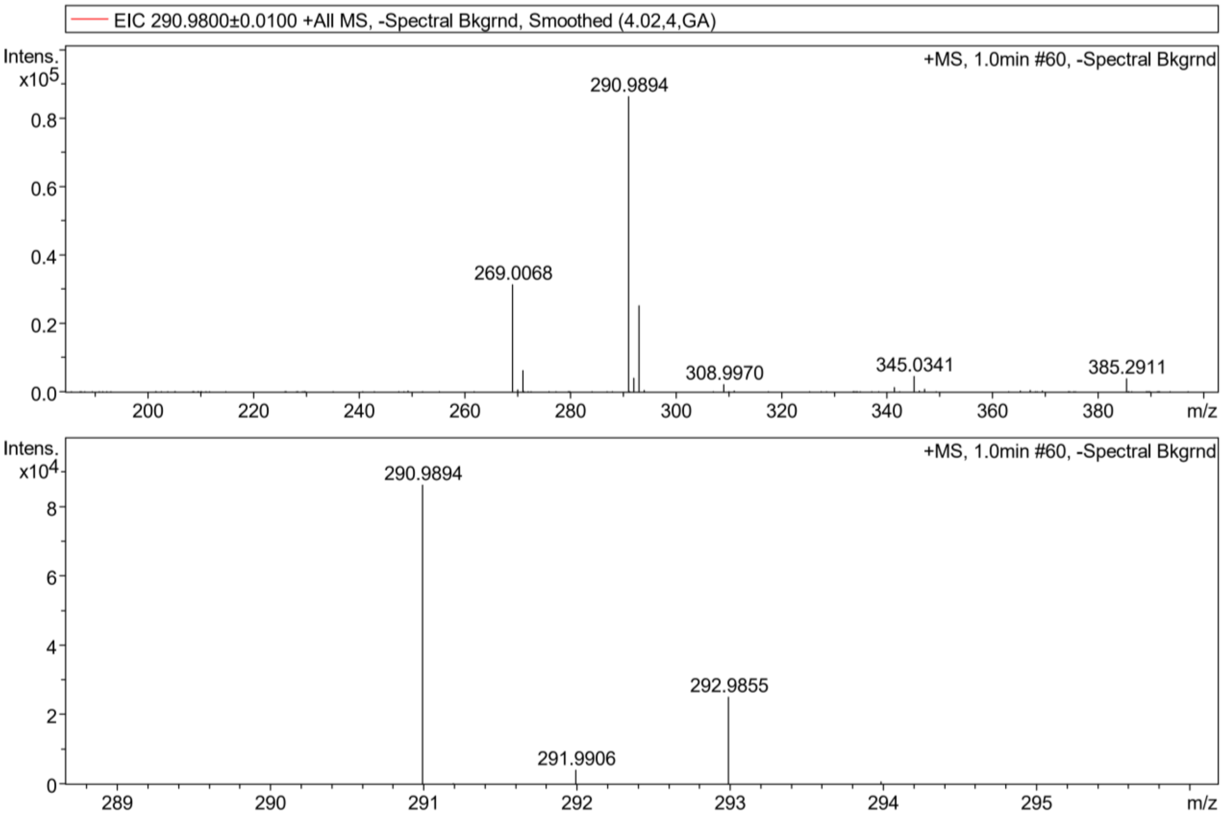

Figure S46. HRMS of 4-((4-chlorophenyl)sulfonyl)phenol (**3r**)

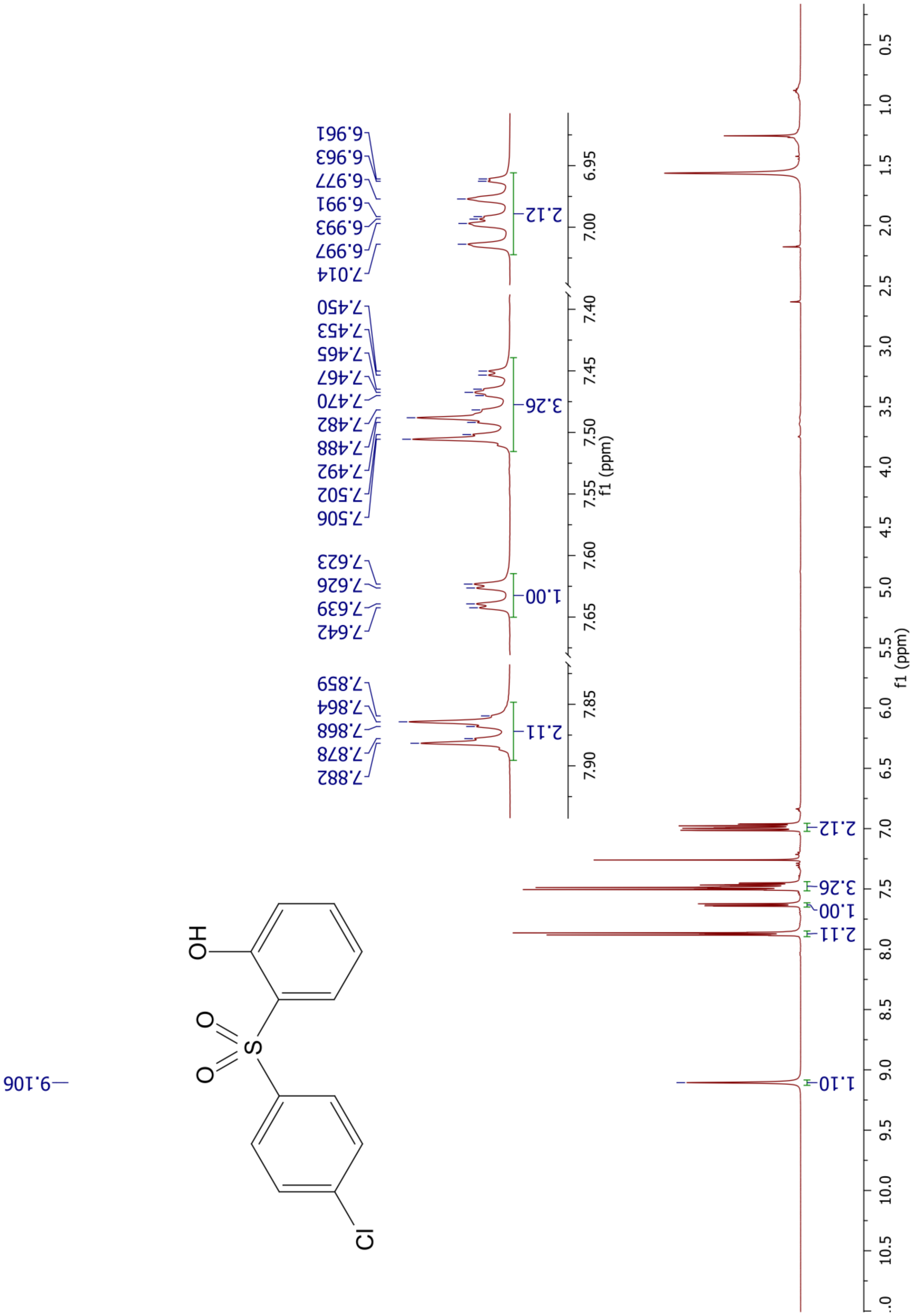

Figure S47. ^{1}H-NMR of 2-((4-chlorophenyl)sulfonyl)phenol (**3r'**)

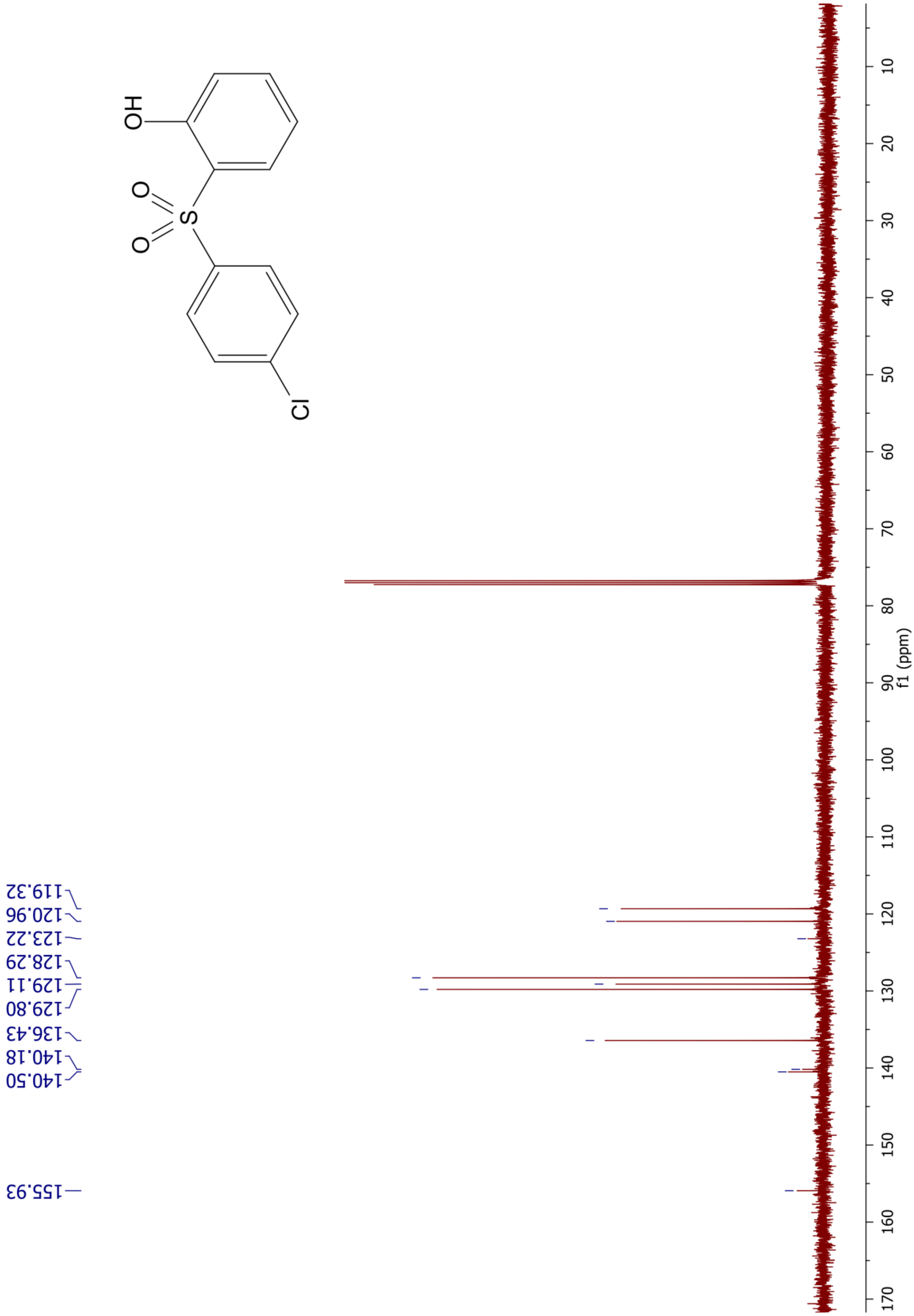

Figure S48. ^{13}C-NMR of 2-((4-chlorophenyl)sulfonyl)phenol (**3r’**)

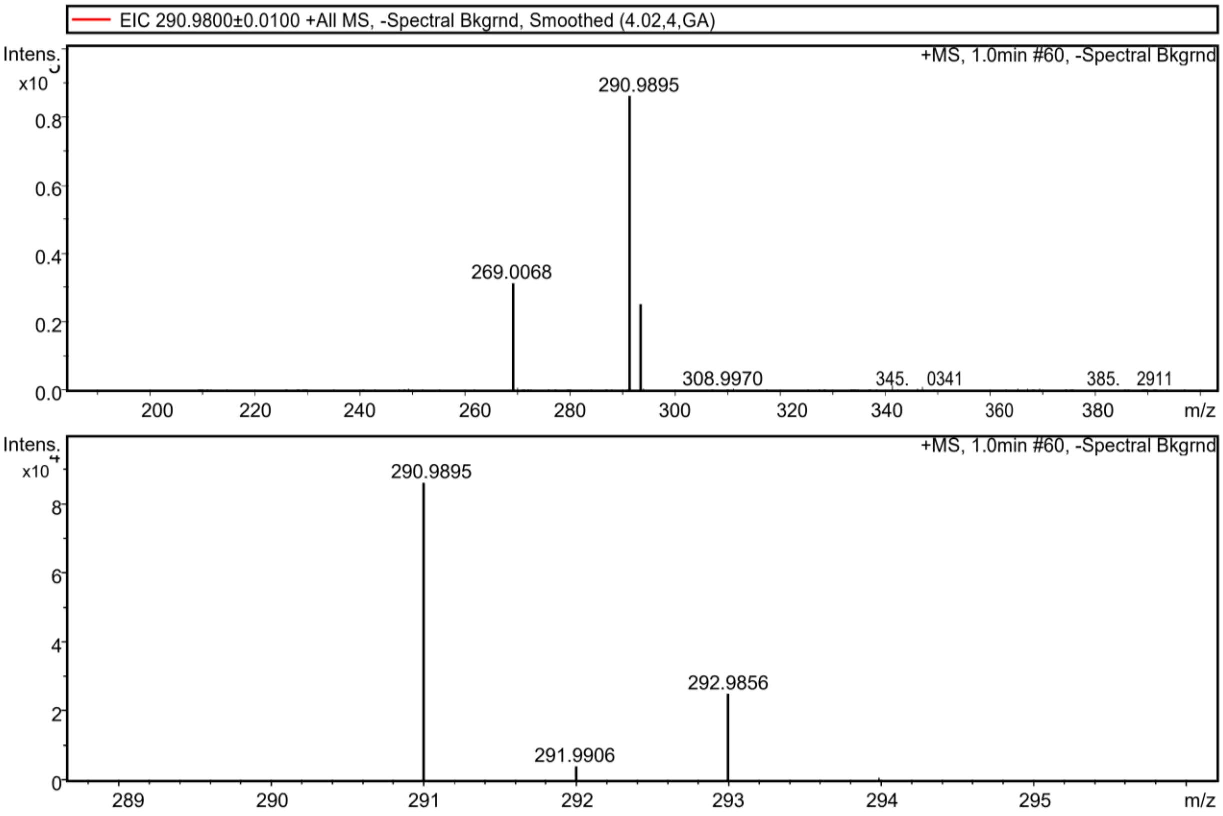

Figure S49. HRMS of 2-((4-chlorophenyl)sulfonyl)phenol (**3r'**)

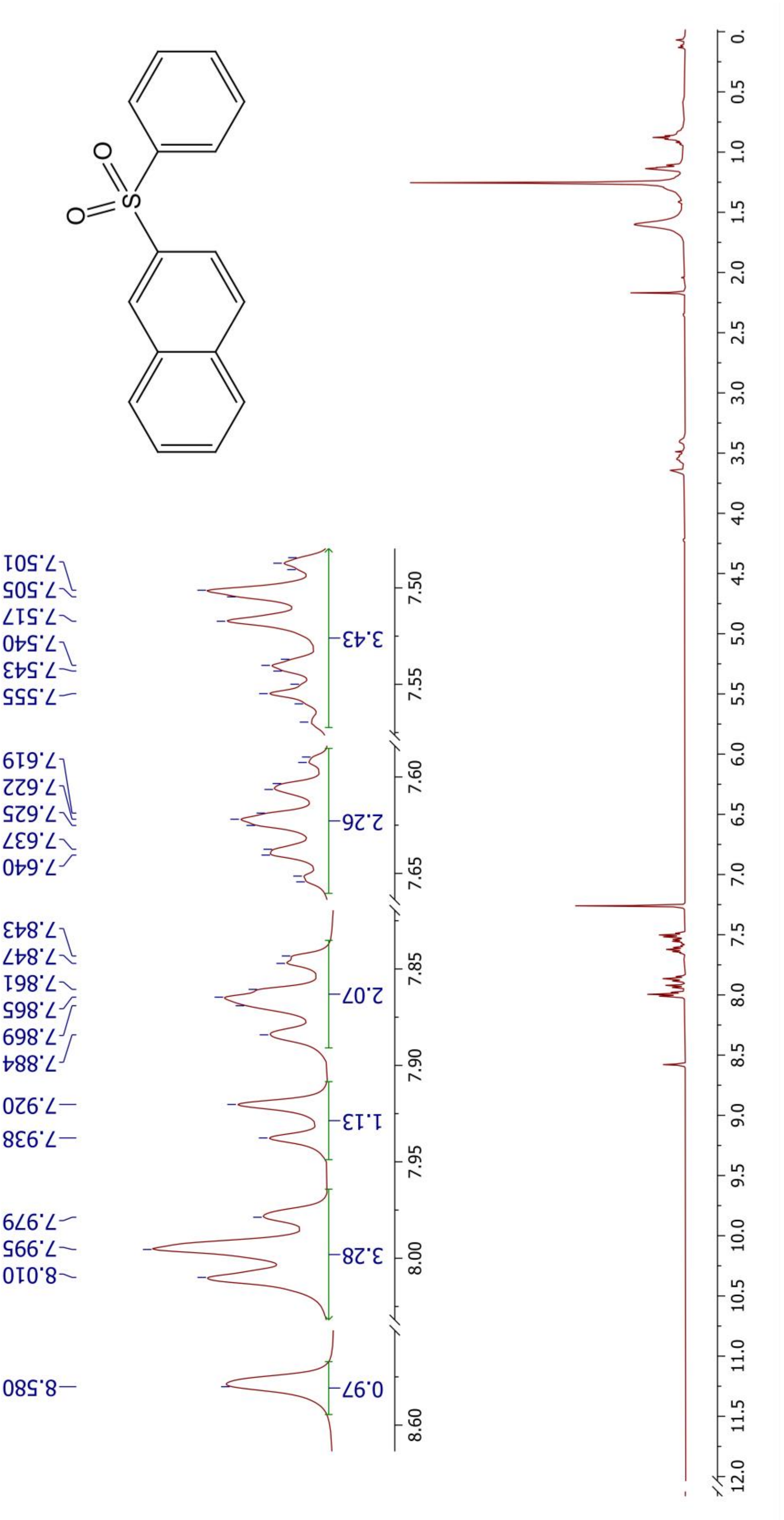

Figure S50. ^{1}H-NMR of 2-(phenylsulfonyl)naphthalene (**3s'**)

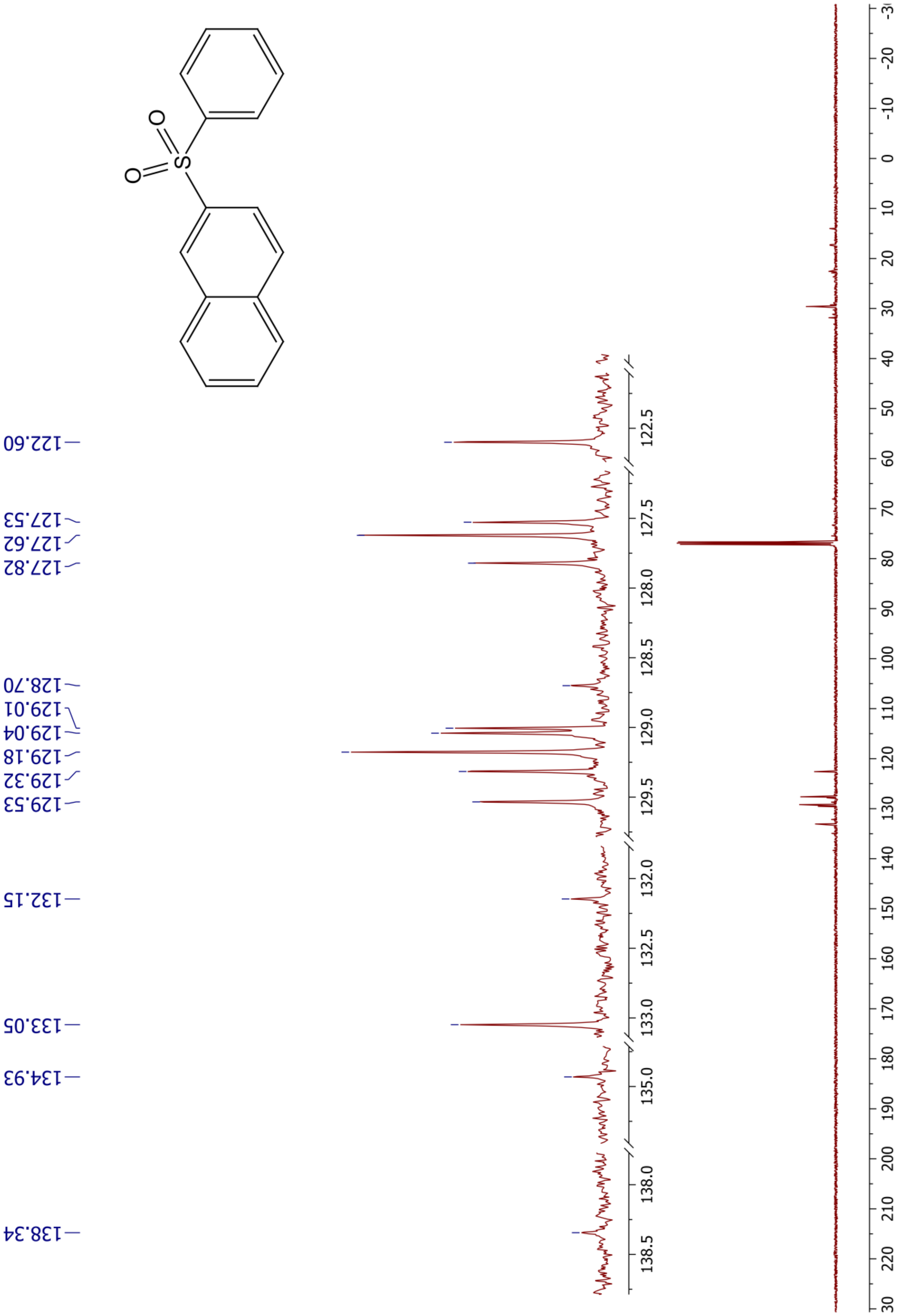

Figure S51. ^{13}C-NMR of 2-(phenylsulfonyl)naphthalene (**3s'**)

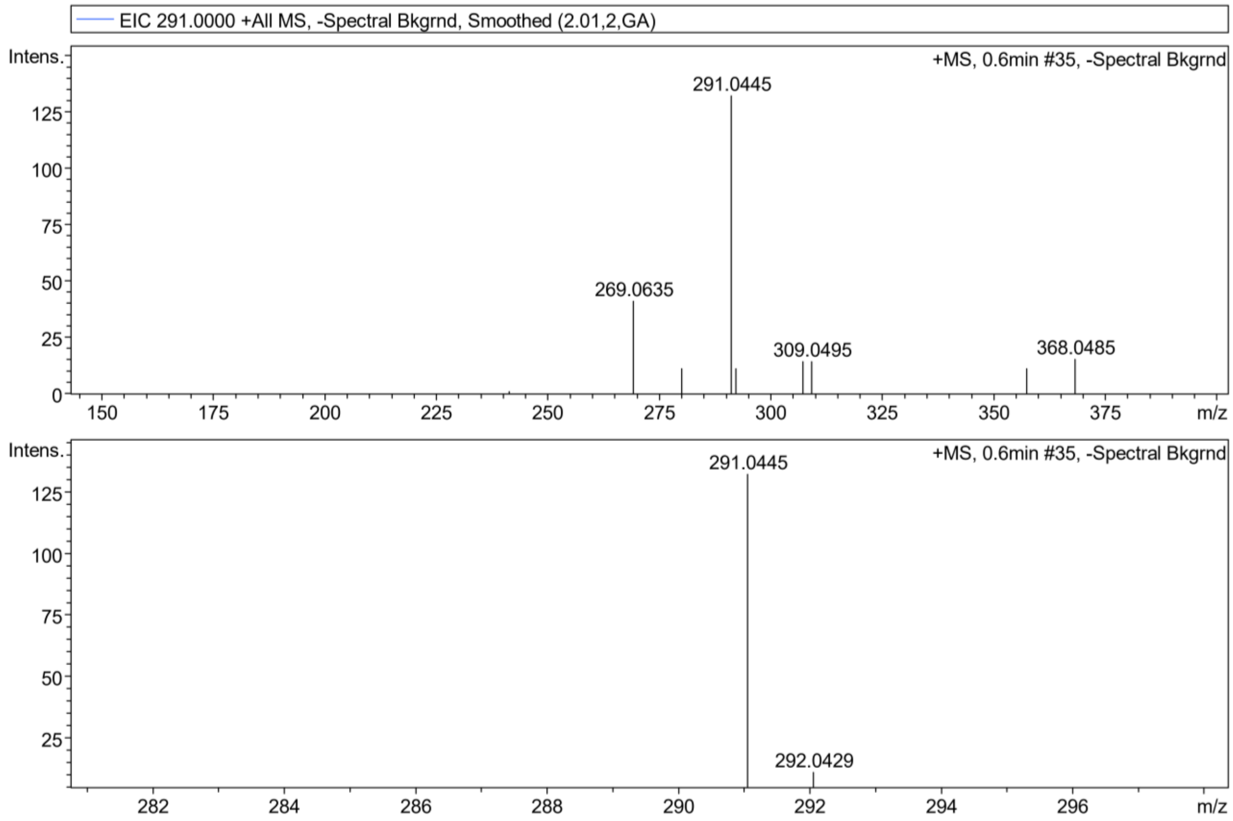

Figure S52. HRMS of 2-(phenylsulfonyl)naphthalene (**3s'**)

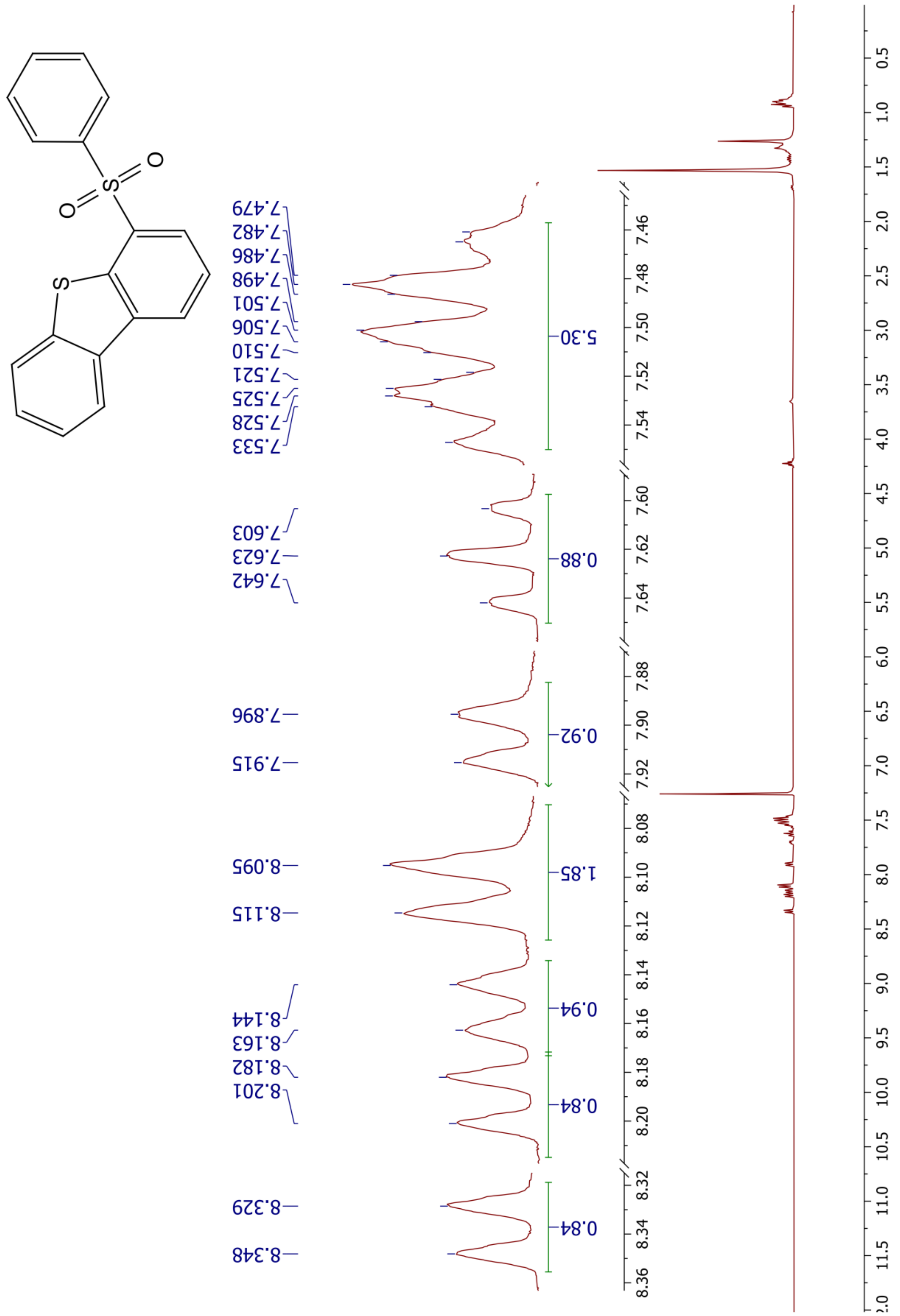

Figure S53. ^{1}H-NMR of 4-(phenylsulfonyl)dibenzo[b,d]thiophene (**3t'**)

molecules

MDPI

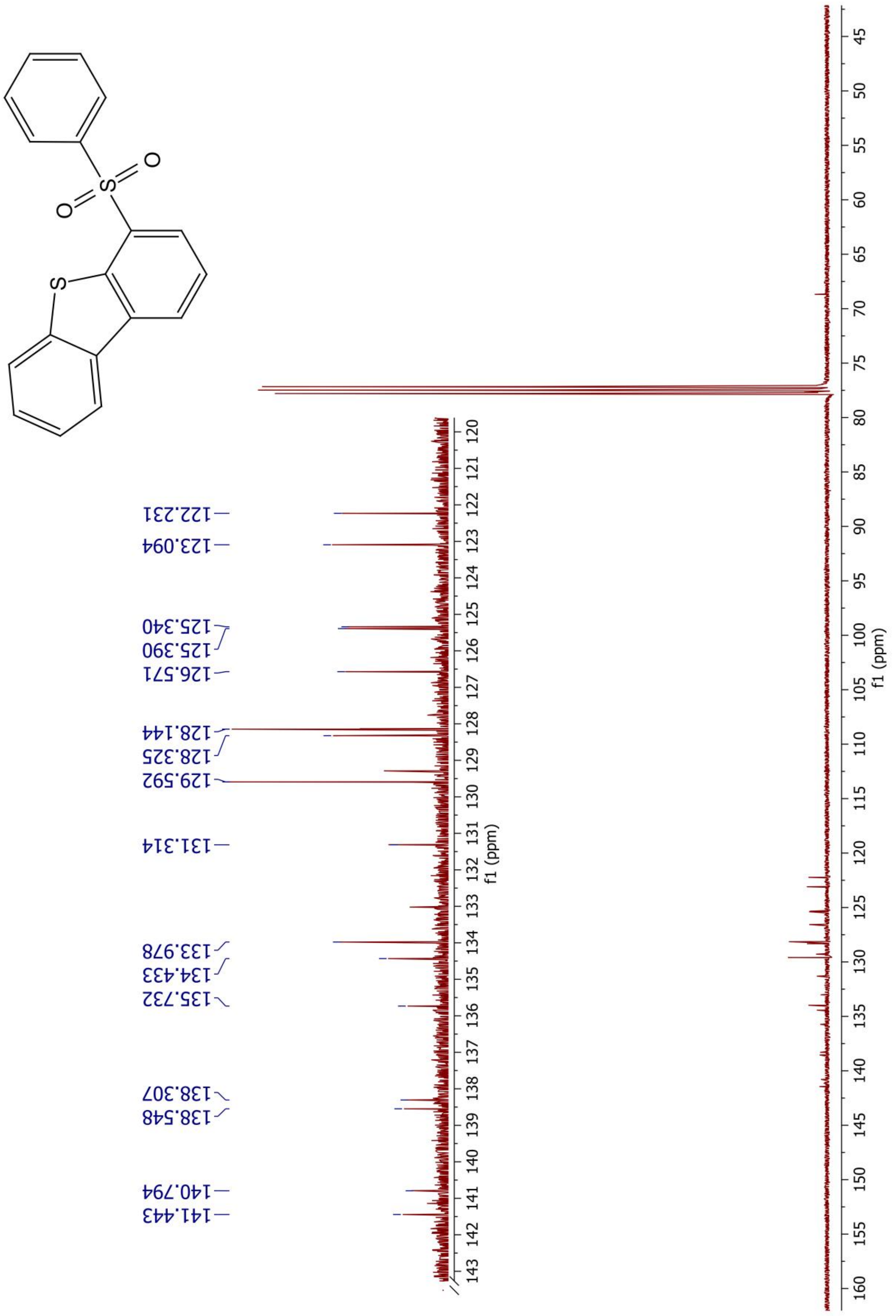

Figure S54. ^{13}C-NMR of 4-(phenylsulfonyl)dibenzo[b,d]thiophene (**3t’**)

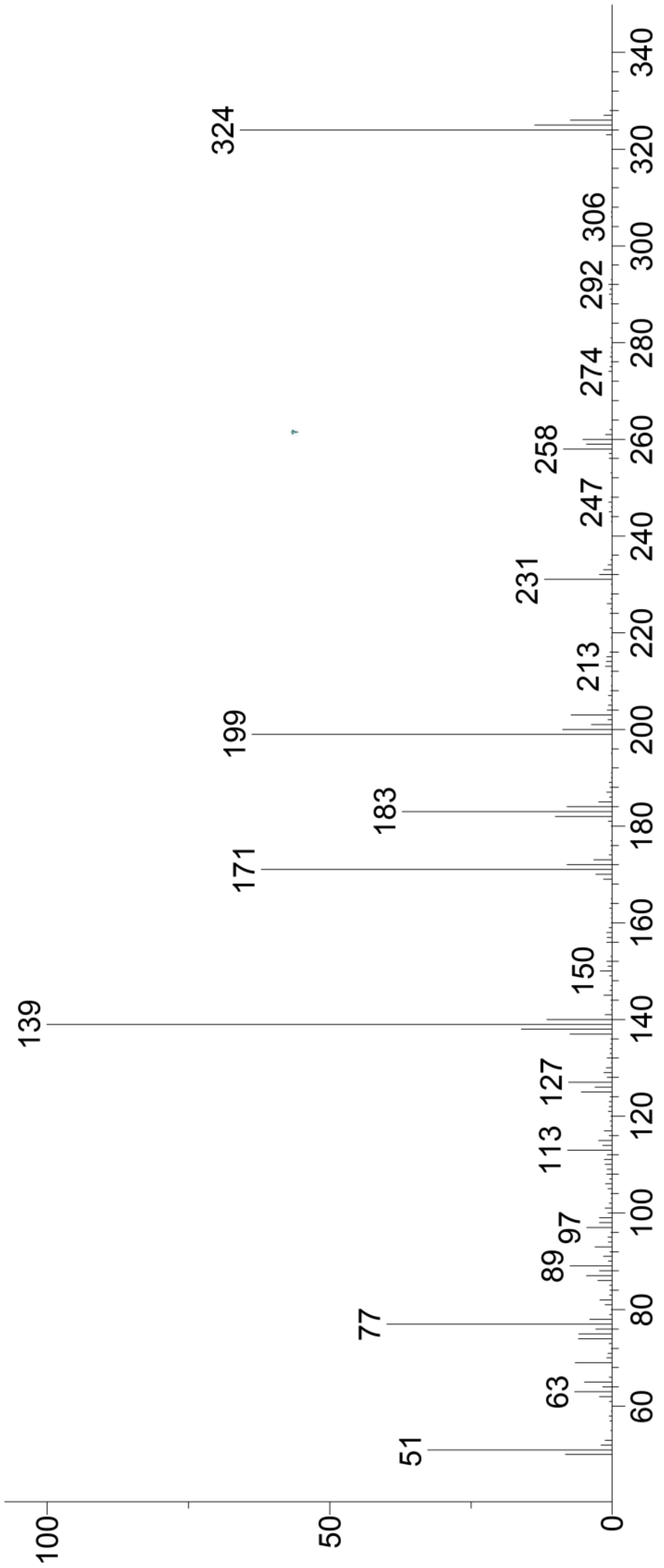

Figure S55. GC-MS of 4-(phenylsulfonyl)dibenzo[b,d]thiophene (**3t'**)

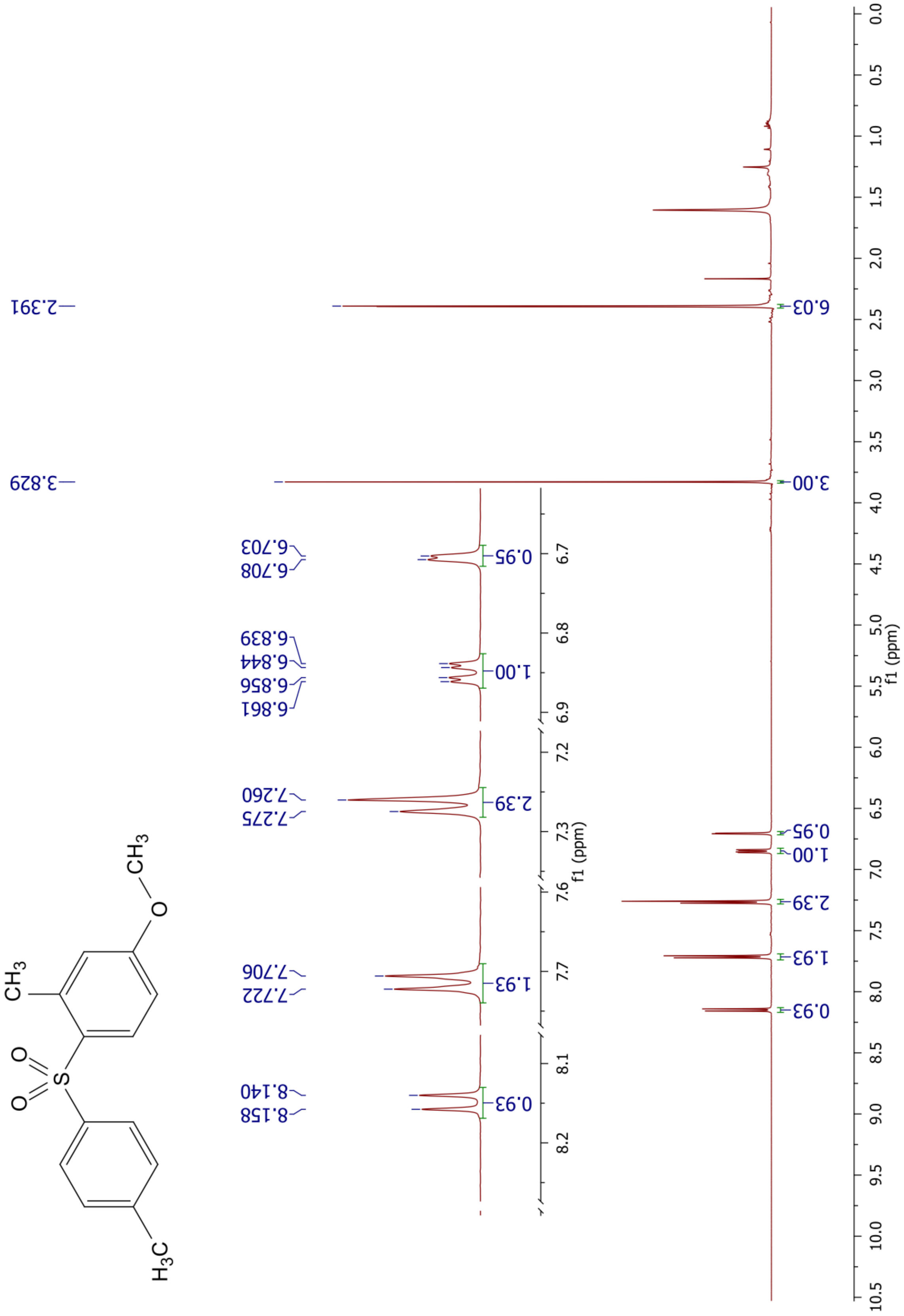

Figure S56. ^{1}H-NMR of 4-methoxy-2-methyl-1-tosylbenzene (**3v**)

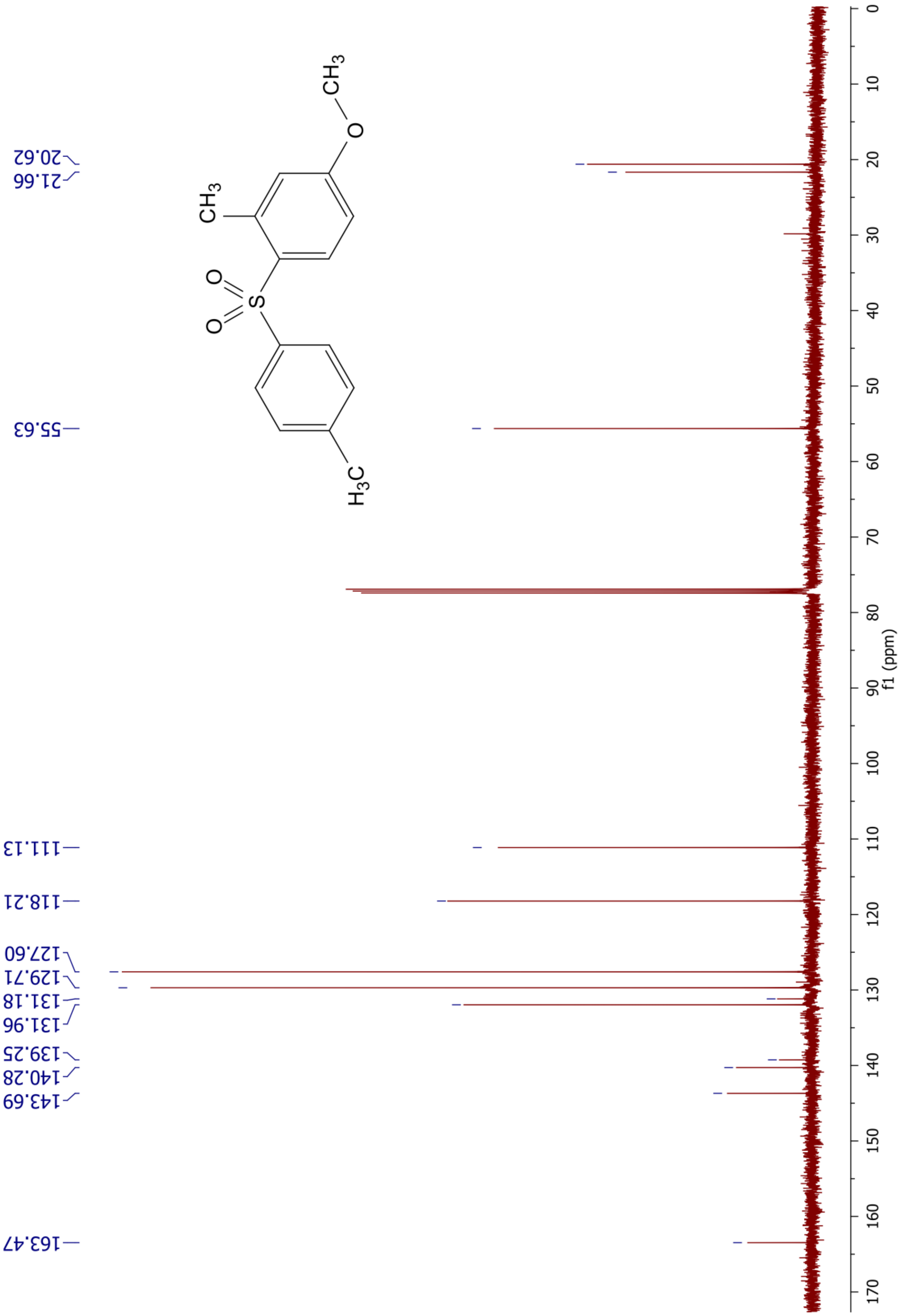

Figure S57. ^{13}C-NMR of 4-methoxy-2-methyl-1-tosylbenzene (**3v**)

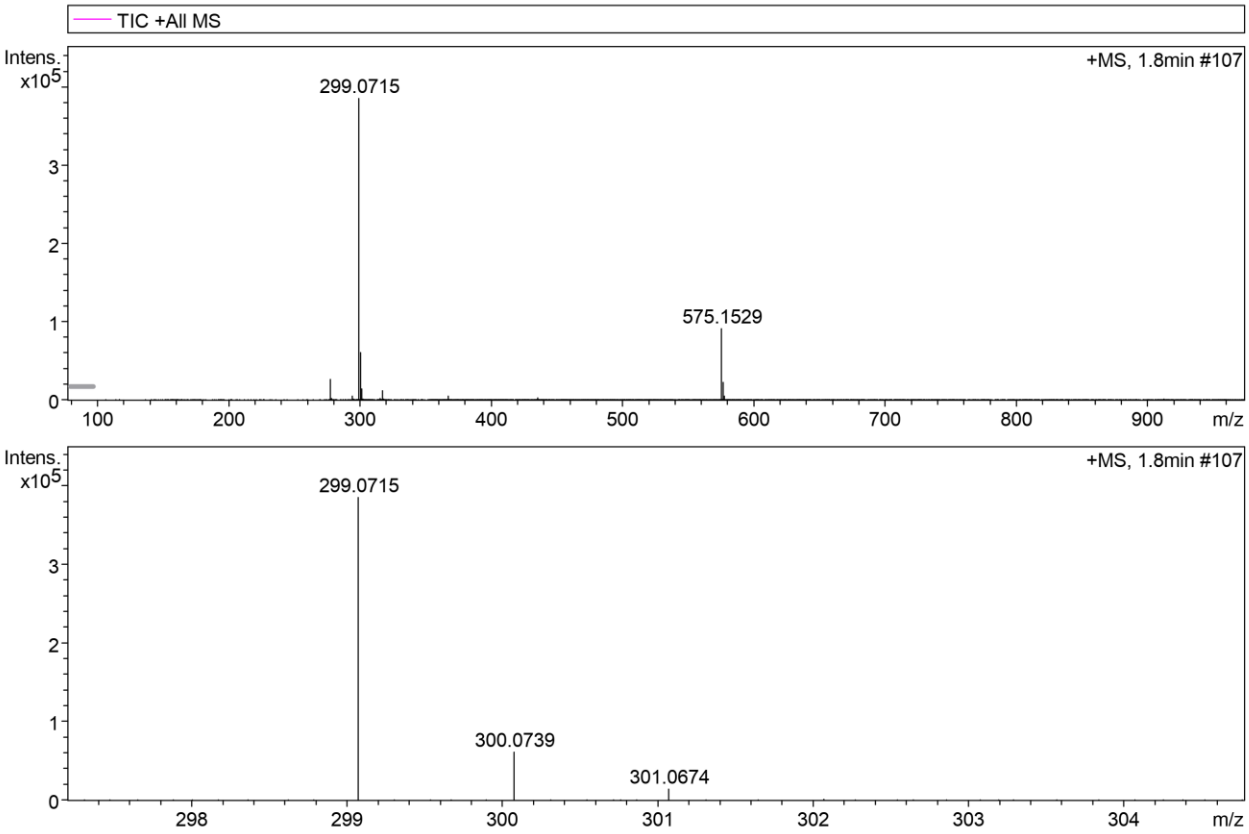

Figure S58. HRMS of 4-methoxy-2-methyl-1-tosylbenzene (**3v**)

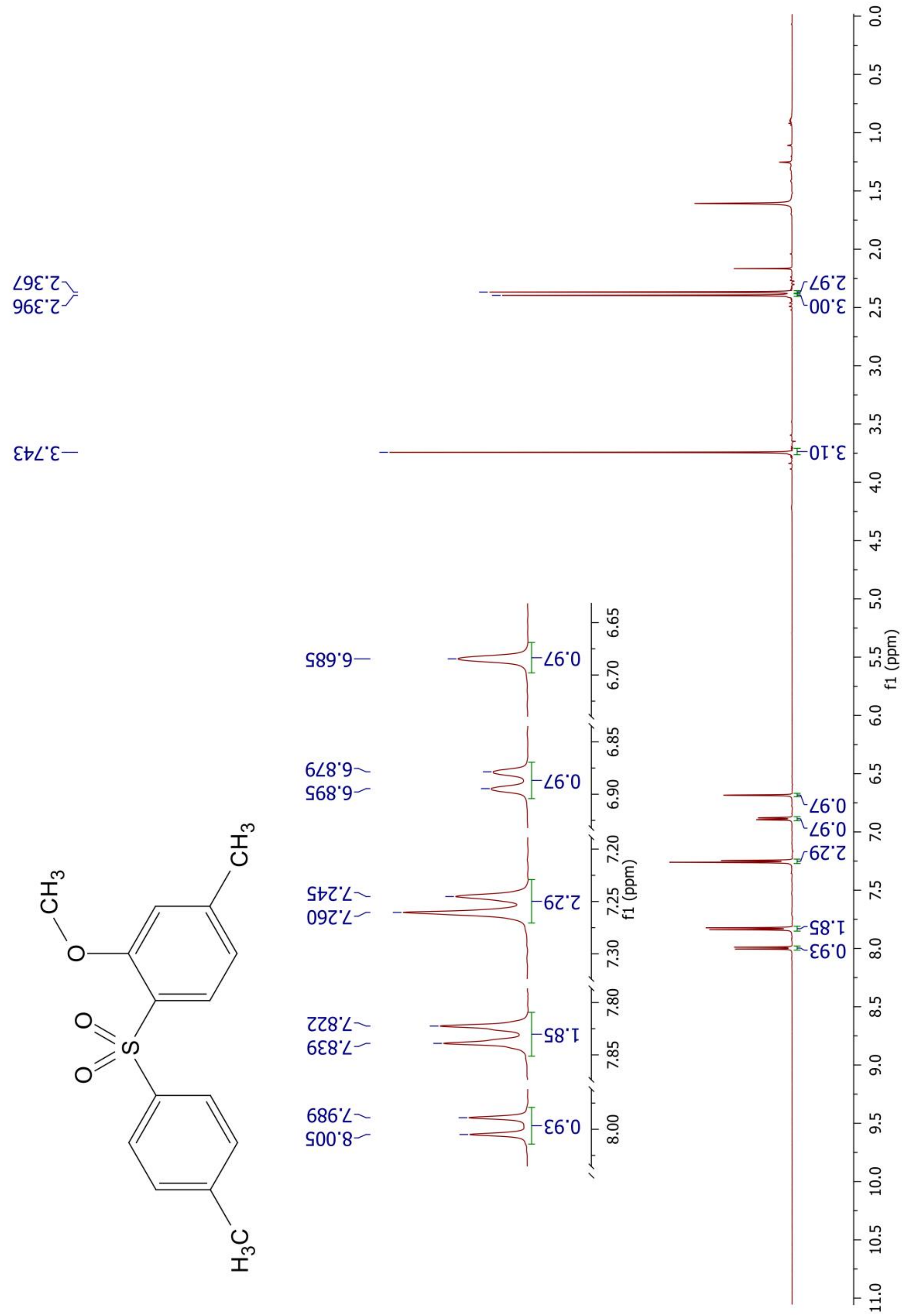

Figure S59. ^{1}H-NMR of 2-methoxy-4-methyl-1-tosylbenzene (**3v'**)

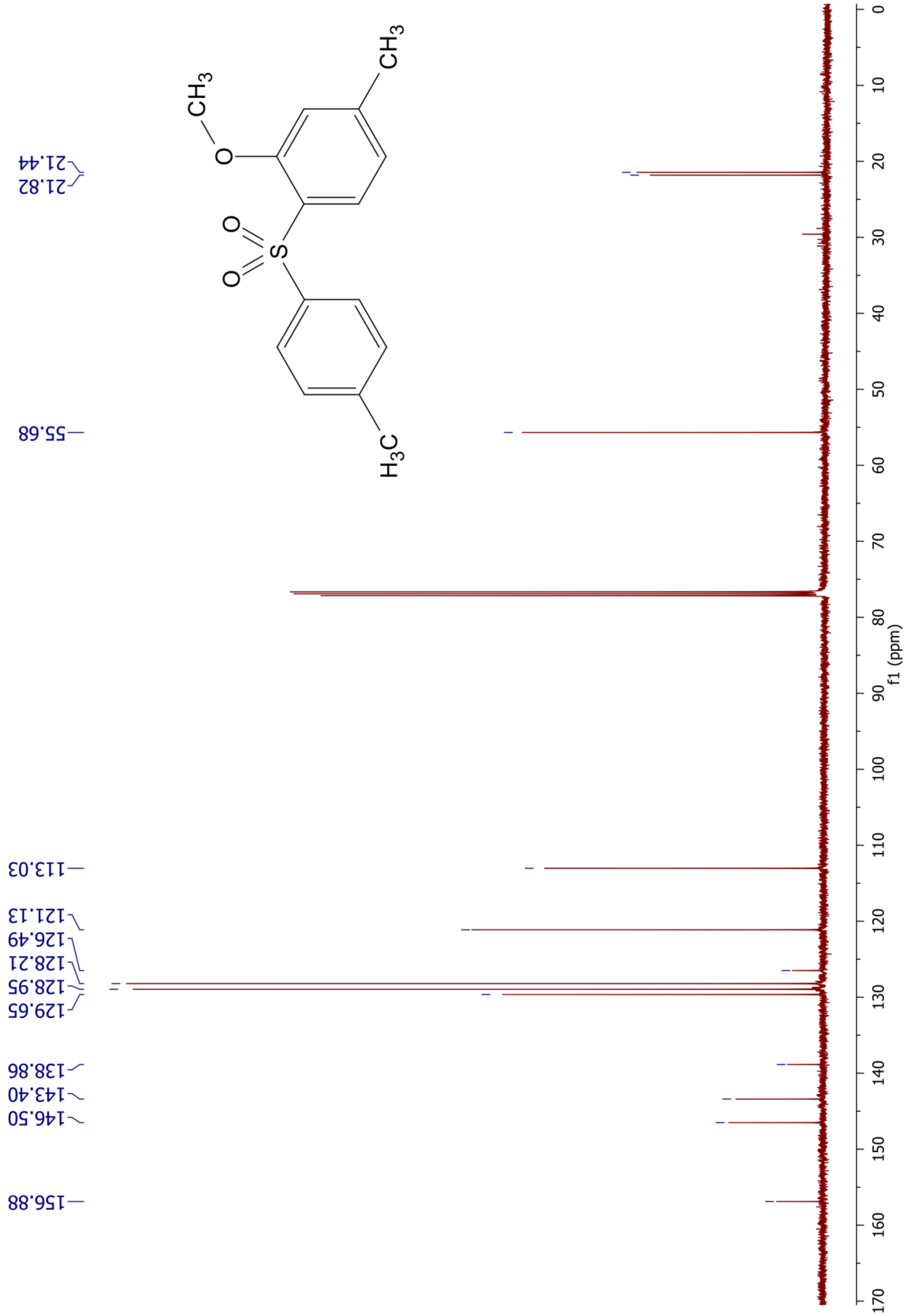

Figure S60. ^{13}C-NMR of 2-methoxy-4-methyl-1-tosylbenzene (**3v'**)

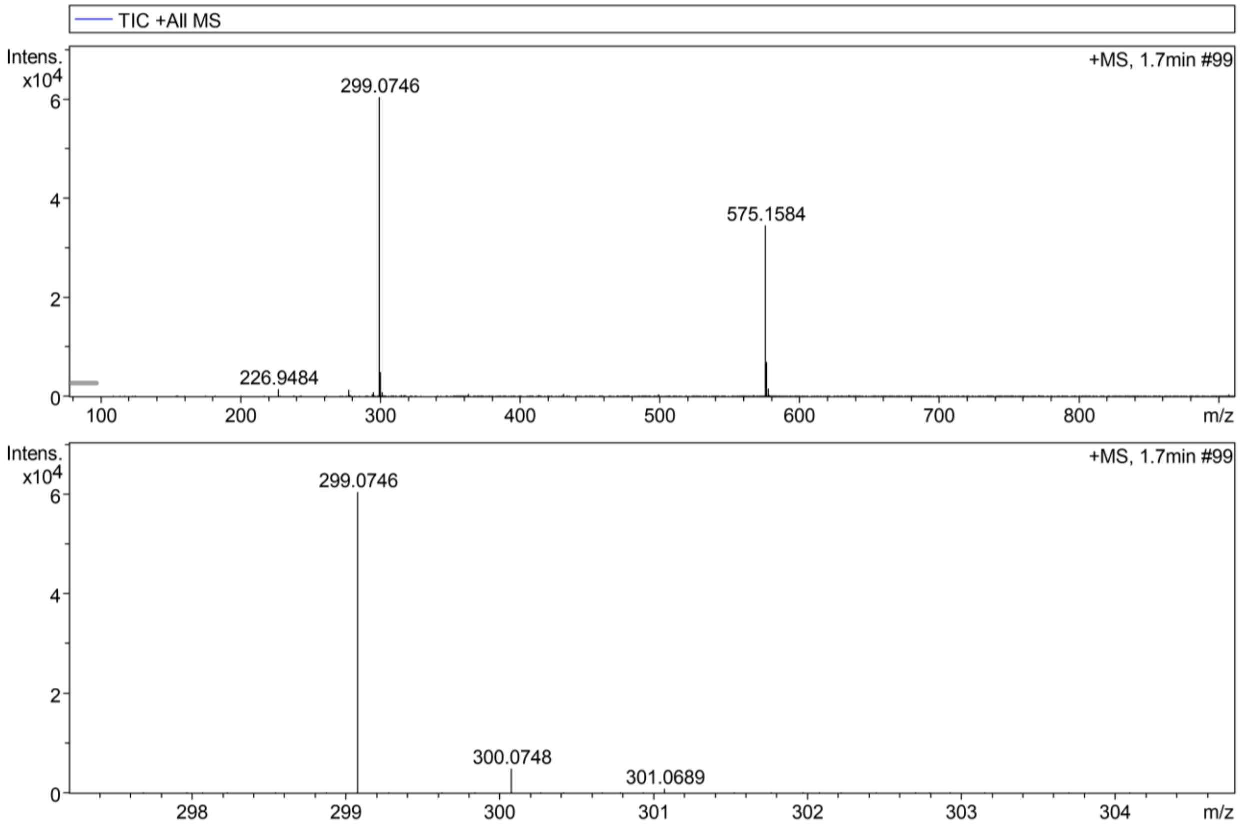

Figure S61. HRMS of 2-methoxy-4-methyl-1-tosylbenzene (**3v'**)

molecules MDPI

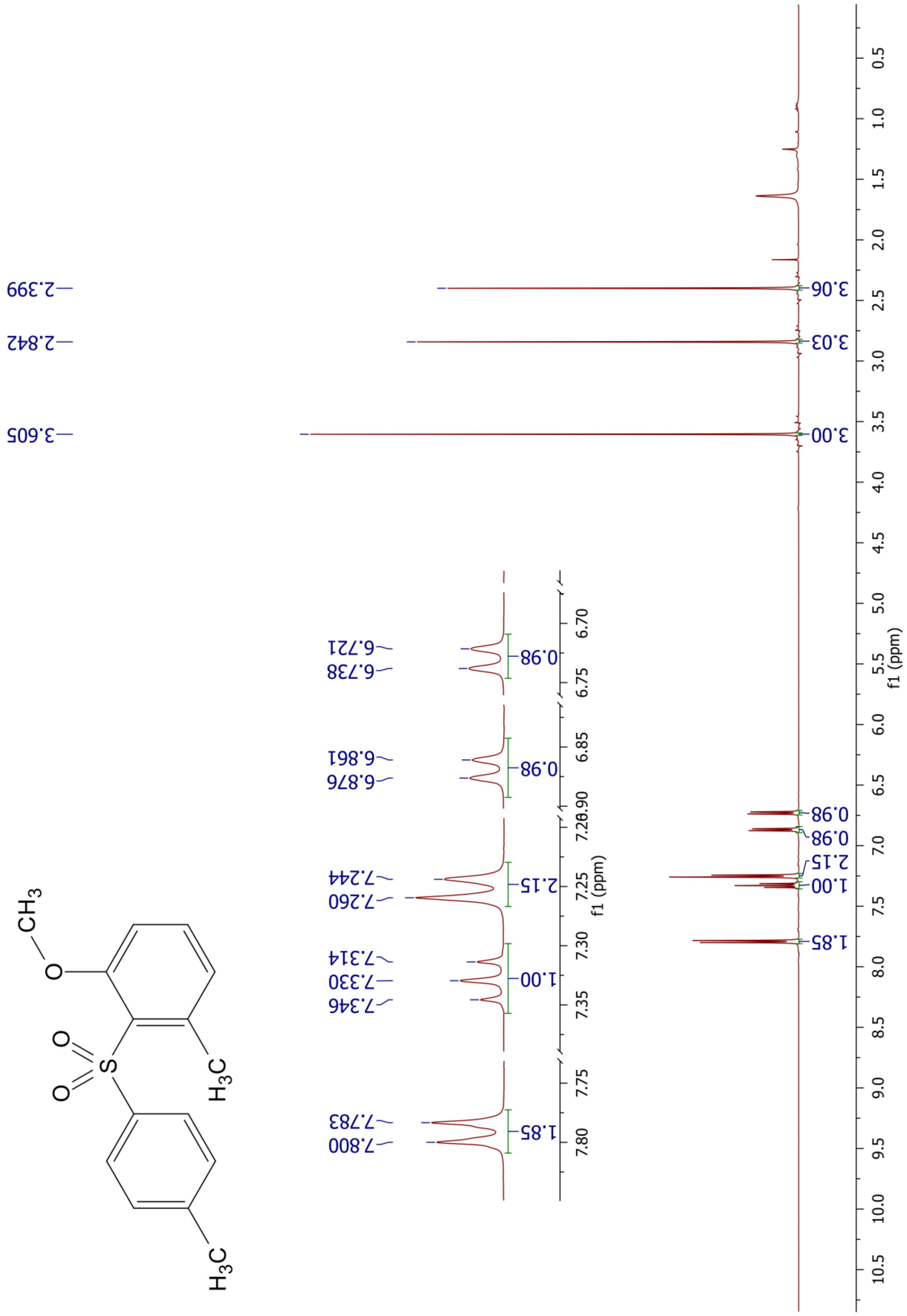

Figure S62. ^{1}H-NMR of 1-methoxy-3-methyl-1-tosylbenzene (**3v’’**)

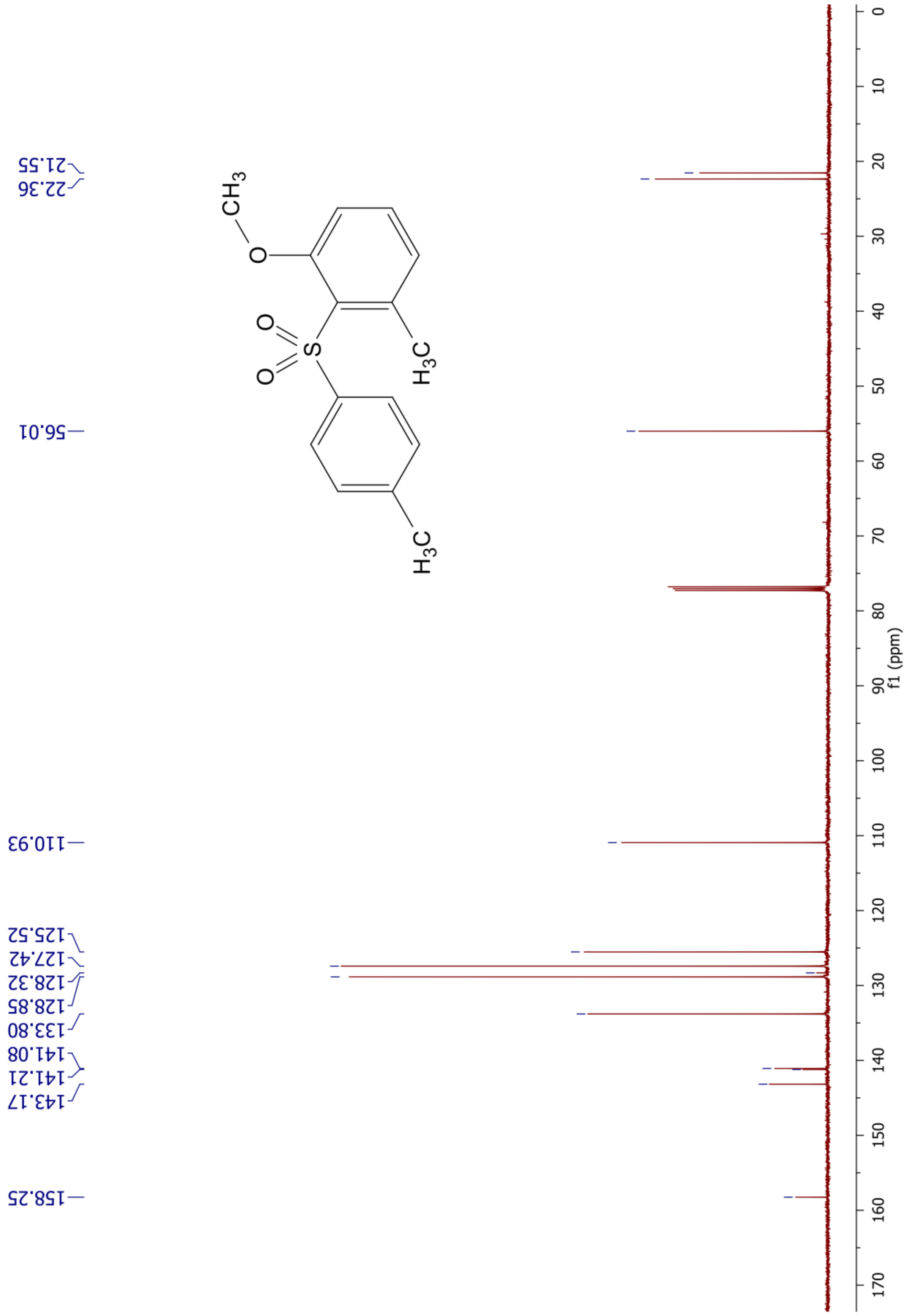

Figure S63. ^{13}C-NMR of 1-methoxy-3-methyl-1-tosylbenzene (**3v''**)

molecules

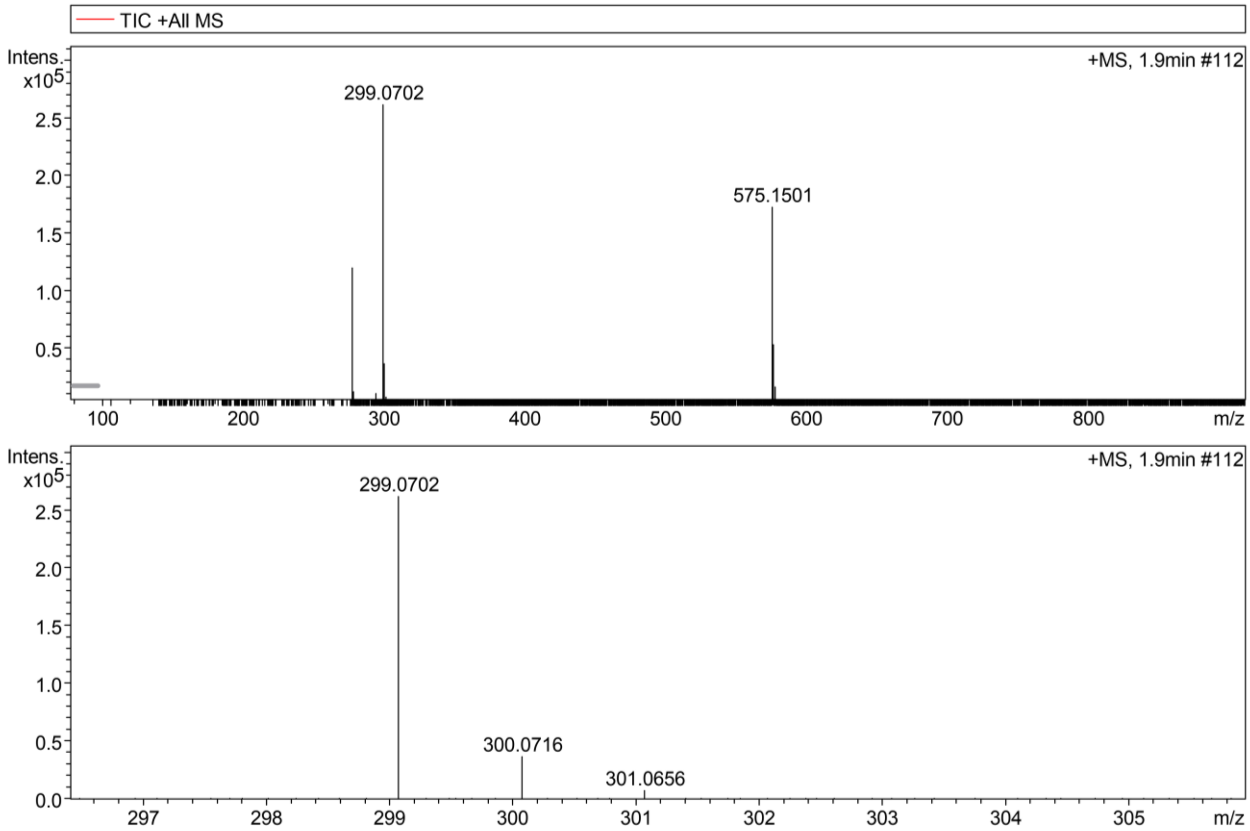

Figure S64. HRMS of 1-methoxy-3-methyl-1-tosylbenzene (**3v''**)

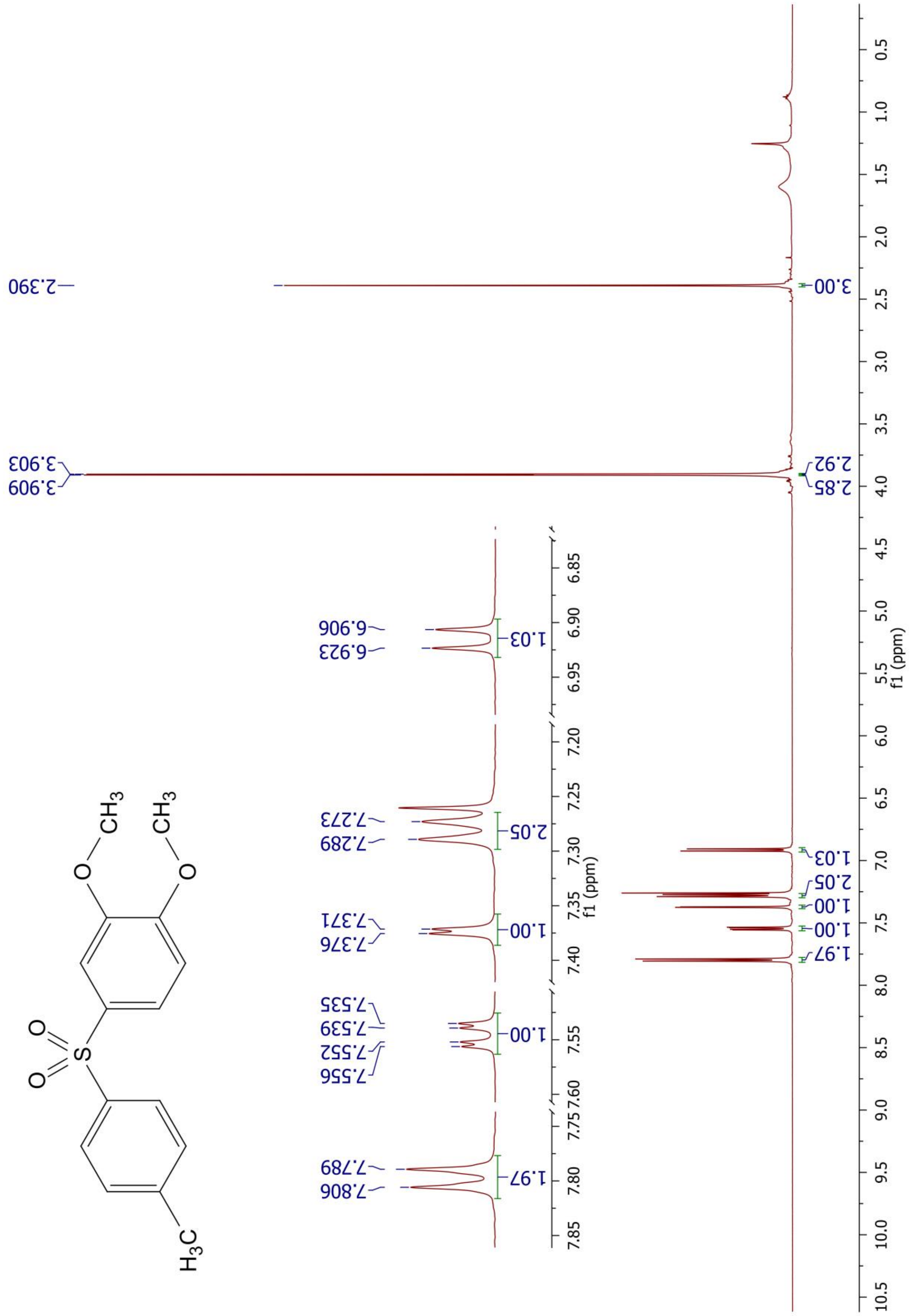

Figure S65. ^{1}H-NMR of 1,2-dimethoxy-4-tosylbenzene (**3w**)

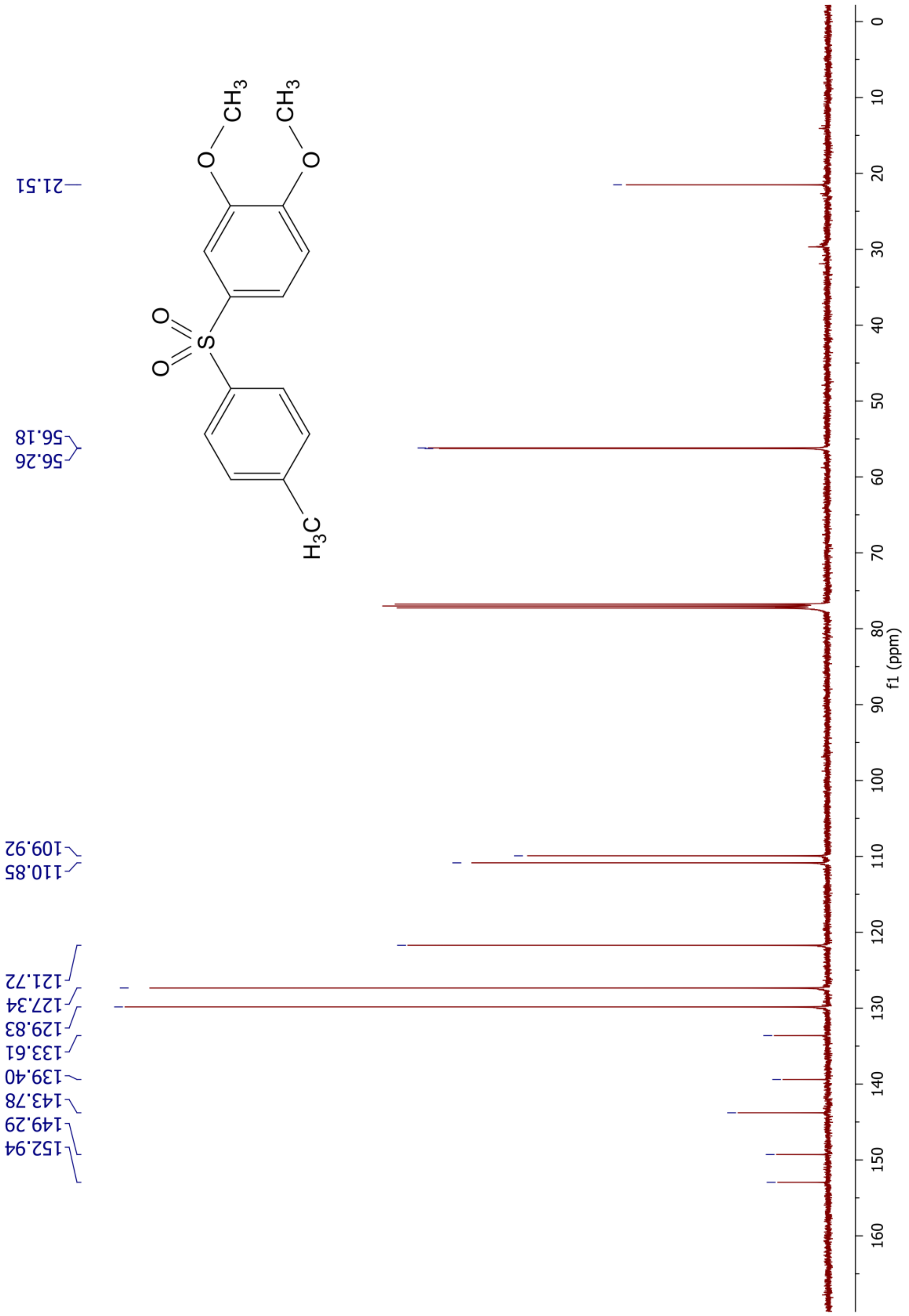

Figure S66. ^{13}C-NMR of 1,2-dimethoxy-4-tosylbenzene (**3w**)

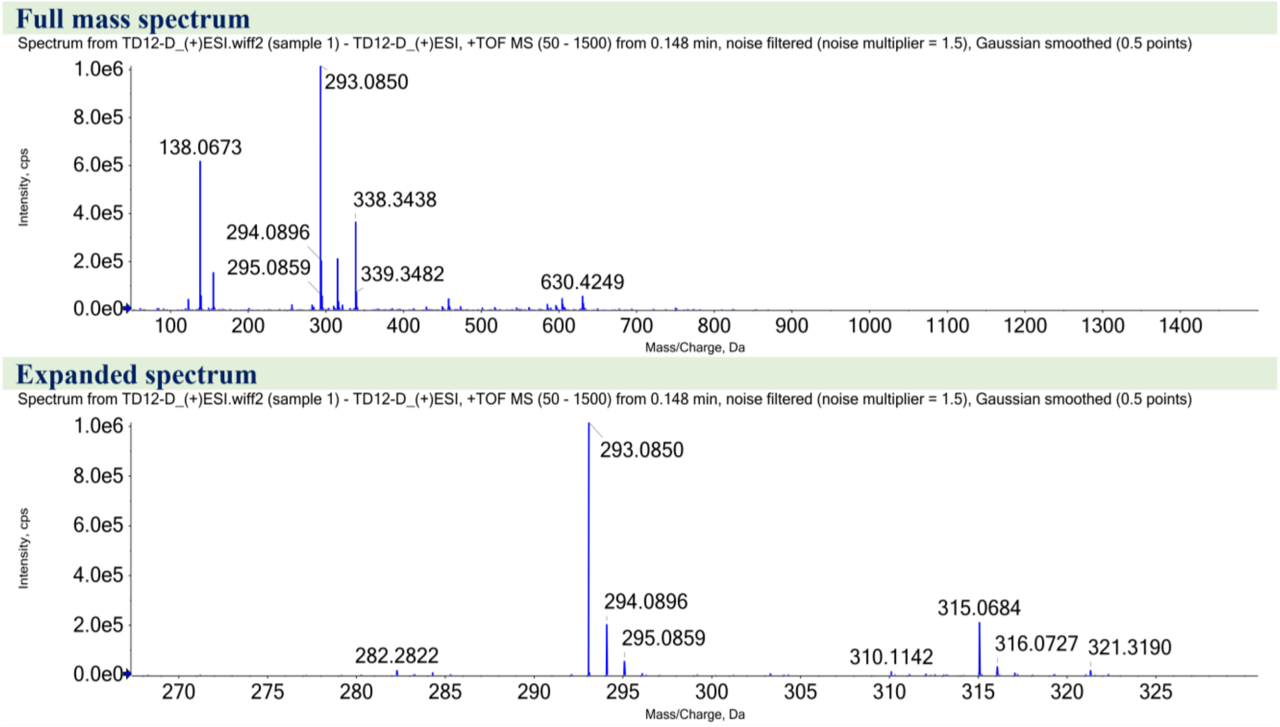

Figure S67. HRMS of 1,2-dimethoxy-4-tosylbenzene (**3w**)

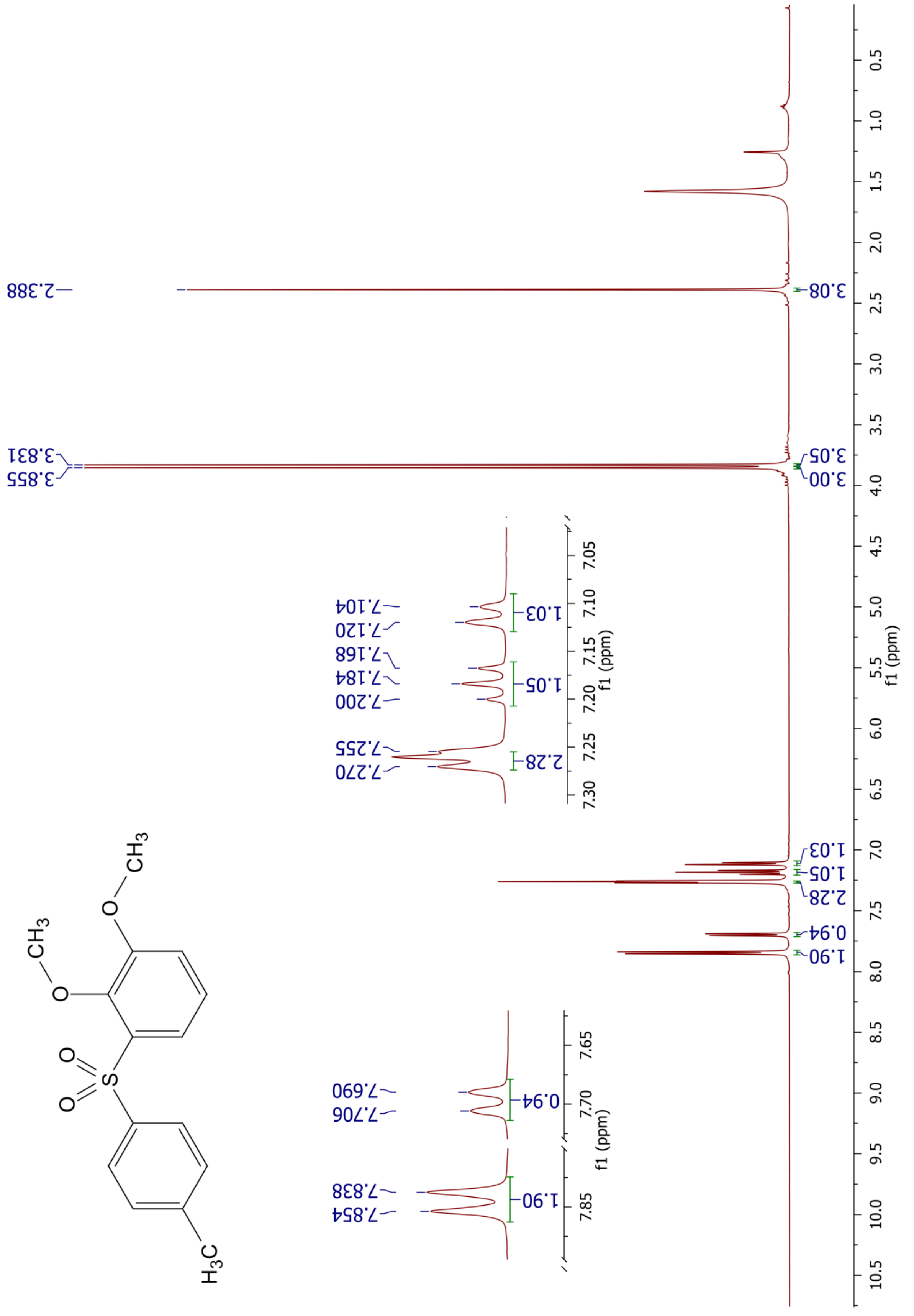

Figure S68. ^{1}H-NMR of 1,2-dimethoxy-3-tosylbenzene (**3w'**)

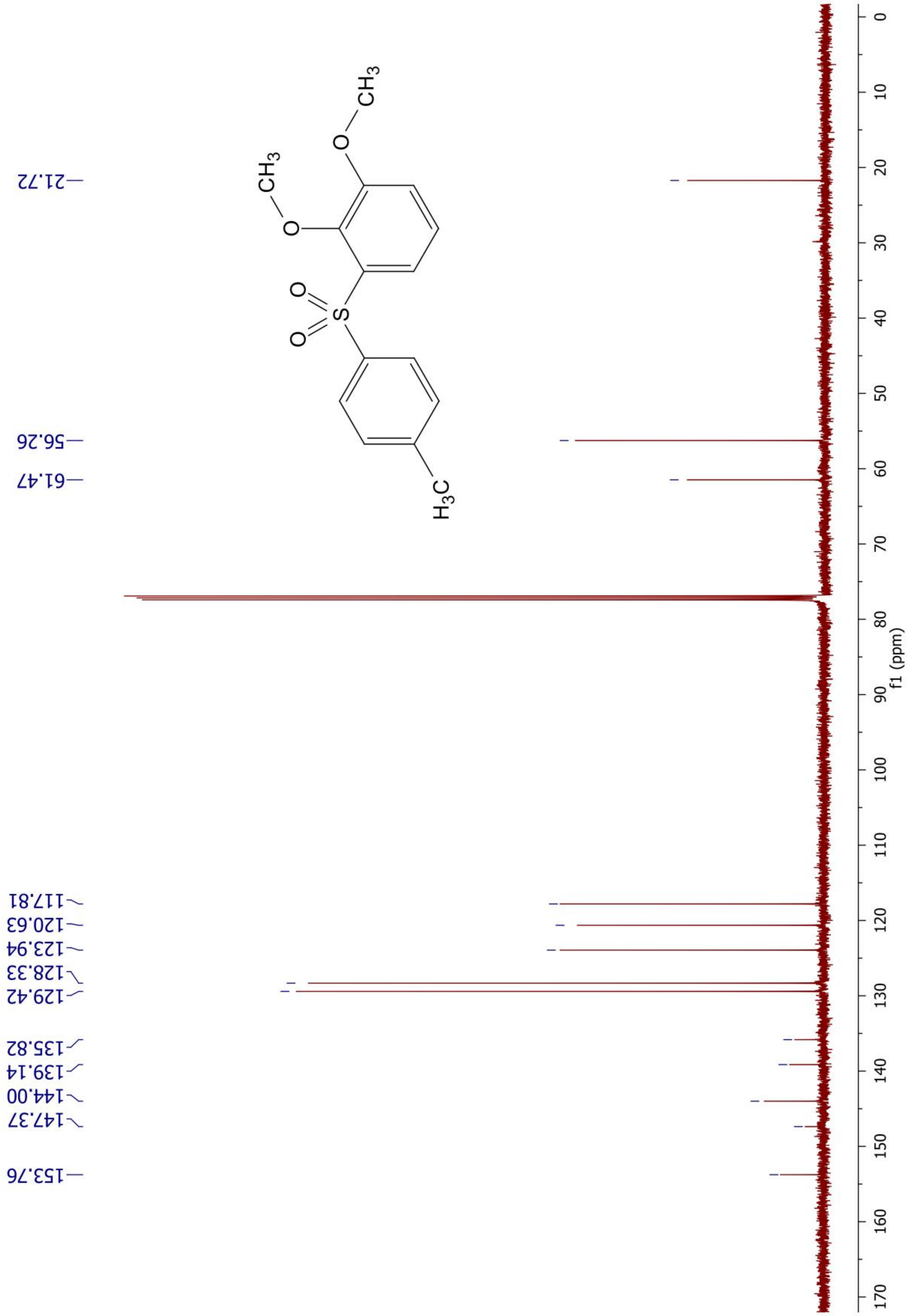

Figure S69. ^{13}C-NMR of 1,2-dimethoxy-3-tosylbenzene (**3w'**)

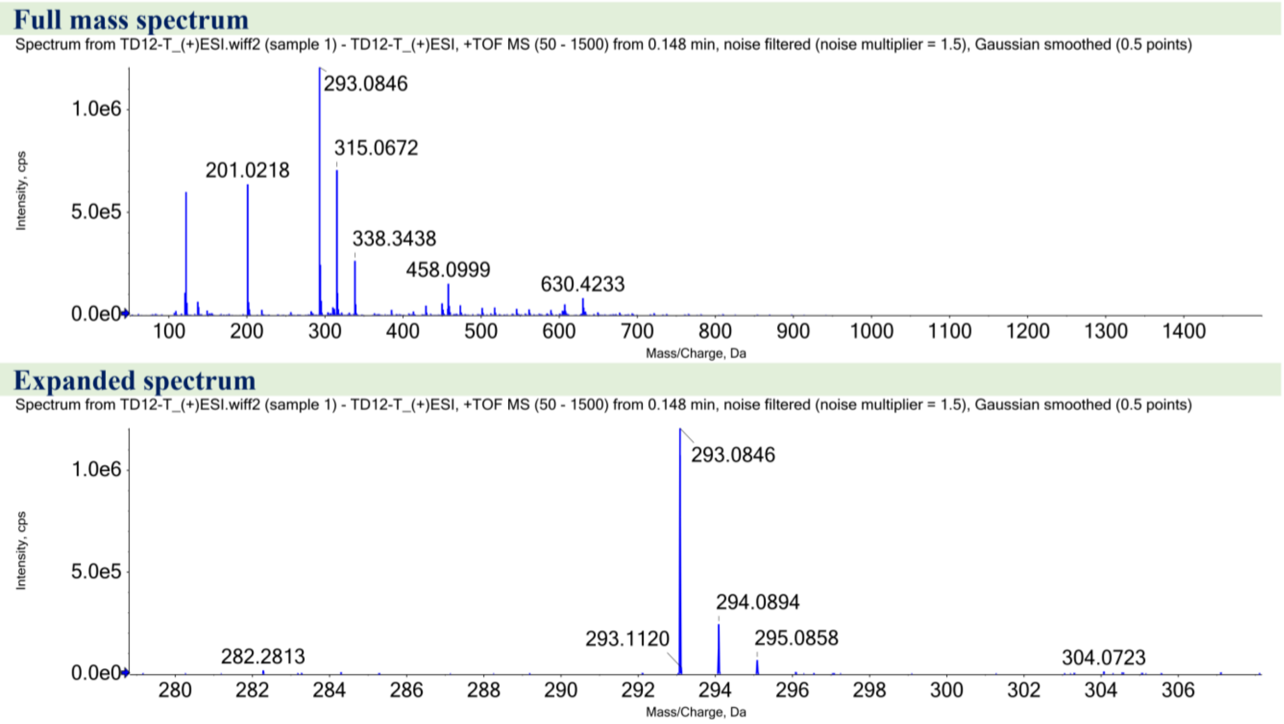

Figure S70. HRMS of 1,2-dimethoxy-3-tosylbenzene (**3w’**)
